# Deep learning-based fully automated grading system for dry eye disease severity

**DOI:** 10.1371/journal.pone.0299776

**Published:** 2024-03-14

**Authors:** Seonghwan Kim, Daseul Park, Youmin Shin, Mee Kum Kim, Hyun Sun Jeon, Young-Gon Kim, Chang Ho Yoon

**Affiliations:** 1 Department of Ophthalmology, Seoul National University College of Medicine, Seoul, Korea; 2 Department of Ophthalmology, Seoul Metropolitan Government Seoul National University Boramae Medical Center, Seoul, Korea; 3 Laboratory of Ocular Regenerative Medicine and Immunology, Biomedical Research Institute, Seoul National University Hospital, Seoul, Korea; 4 Department of Transdisciplinary Medicine, Seoul National University Hospital, Seoul, Korea; 5 Interdisciplinary Program in Bioengineering, Graduate School, Seoul National University, Seoul, Korea; 6 Department of Ophthalmology, Seoul National University Hospital, Seoul, Korea; 7 Department of Ophthalmology, Seoul National University Bundang Hospital, Seongnam-si, Gyeonggi-do, Korea; Hangil Eye Hospital / Catholic Kwandong University College of Medicine, REPUBLIC OF KOREA

## Abstract

There is an increasing need for an objective grading system to evaluate the severity of dry eye disease (DED). In this study, a fully automated deep learning-based system for the assessment of DED severity was developed. Corneal fluorescein staining (CFS) images of DED patients from one hospital for system development (n = 1400) and from another hospital for external validation (n = 94) were collected. Three experts graded the CFS images using NEI scale, and the median value was used as ground truth. The system was developed in three steps: (1) corneal segmentation, (2) CFS candidate region classification, and (3) estimation of NEI grades by CFS density map generation. Also, two images taken on different days in 50 eyes (100 images) were compared to evaluate the probability of improvement or deterioration. The Dice coefficient of the segmentation model was 0.962. The correlation between the system and the ground truth data was 0.868 (p<0.001) and 0.863 (p<0.001) for the internal and external validation datasets, respectively. The agreement rate for improvement or deterioration was 88% (44/50). The fully automated deep learning-based grading system for DED severity can evaluate the CFS score with high accuracy and thus may have potential for clinical application.

## Introduction

Machine learning has enormously impacted medicine in recent years. The technology has great potential for improving medical diagnosis by providing a means to increasing the accuracy, speed, and reproducibility of diagnosis and to reducing clinician workload [1, 2]. Deep learning is a sub-branch of machine learning that uses neural networks with multilayers to learn a function between a set of inputs and outputs [3]. In ophthalmology, deep learning has been applied to various types of imaging, from color fundus photography to optical coherence tomography and anterior segment photography [4–6].

Dry eye disease (DED) is a multifactorial disorder characterized by loss of the tear film homeostasis accompanied by several ocular symptoms [7, 8]. DED is prevalent in 5.3–34.5% of the population, and its incidence seems to have increased over time [9, 10]. Punctate epithelial erosion (PEE) as evaluated according to the corneal fluorescein staining (CFS) score is one of the critical diagnostic features of DED, in addition to tear break-up time, ocular surface disease index score, and Schirmer test score [11, 12]. Quantifying the degree of CFS is important in grading DED severity. Among various grading methods, the grading system recommended by the National Eye Institute (NEI) is one of the commonly used scales in clinical trials owing to its refined methodology [13–15].

However, most CFS scales, including the NEI scale, are still subjective and observer dependent, showing inter- and intra-observer variabilities [16]. Therefore, a reproducible and reliable objective method to minimize subjective bias from human observers is warranted. However, few studies have applied digitalized automated methods including deep learning technology for objectifying the NEI scale and interpreting CFS [16–20]. Previously reported systems have limitations in that a system developed by Amparo et al. [16] is not fully automated, and another system reported by Qu et al. [19] is not clear what process the system goes through to evaluate the CFS score. Thus, this study aimed to develop a clinically applicable fully automated deep learning-based system for the assessment of dry eye severity according to the NEI scale. Herein, we developed a system that can automatically segment the cornea and evaluate the severity of DED by directly inputting an original image file captured by a digital camera attached to a slit lamp biomicroscope.

## Materials and methods

### Study design and patients

Institutional Review Board (IRB) approval was obtained from Seoul National University Hospital (IRB No. 2205-162-1328). The study was conducted according to the tenets of the Declaration of Helsinki. Informed consent was waived by the IRB because the study was based on the retrospective review of data. The authors had access to information that could identify individual participants (i.e., full names) during or after data collection. The data were accessed for research purposes from August 2022 and February 2023 (S1–S3 Datasets).

A total of 1400 anterior segment images of DED patients, including Sjögren syndrome and ocular graft-versus-host disease (GVHD), at Seoul National University Hospital (hospital 1) between January 2019 and December 2021 were retrospectively collected. In addition, 94 anterior segment images of DED patients at Seoul National University Bundang Hospital (hospital 2) were collected for external validation. The exclusion criteria were (1) a history of ocular surgery and (2) presence of corneal diseases such as corneal opacity, corneal edema, and keratitis.

### Anterior segment image capture technique

A fluorescein strip (FLUO 900 Strip ®, Haag-Streit AG, Bern, Switzerland) was moistened with normal saline, shaken off and applied to the inferior fornix to stain the cornea. After 3–5 blinks, anterior segment images were obtained using the digital unit of a 5-megapixel camera (DC-4, Topcon, Tokyo, Japan) attached to a slit lamp biomicroscope (Topcon, SL-D701, Tokyo, Japan) under a cobalt filter. All image files were saved in JPEG format (2576 × 1934 pixels, 24-bit).

### NEI scoring method

**Fig 1:**
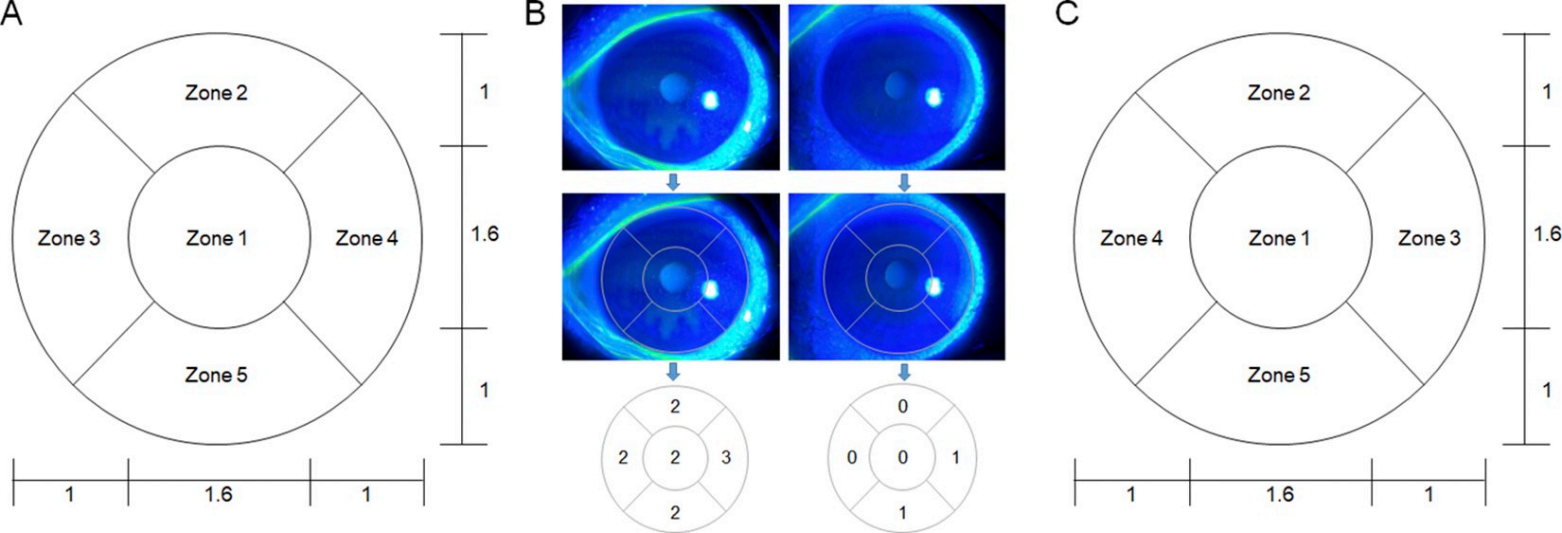
Corneal segmentation and scoring method. (A) Corneal segmentation grid and proportion (right eye). The horizontal and vertical ratios of each zone of the grid are 1:1.6:1. (B) Two examples of NEI scale evaluation. PEE of the five zones is assessed and scored using the NEI scale. (C) Corneal segmentation grid and proportion (left eye). NEI, National Eye Institute; PEE, punctate epithelial erosion.

The NEI scale was used to evaluate the CFS score [13]. Considering the arbitrary grid of the NEI scale in the 1995 NEI workshop, the grid proportion of the NEI scale was used according to Amparo et al. [16] (Fig 1). A grid was added to each anterior segment photograph. Using a grid, the corneal area was divided into five zones, and the score at each zone was evaluated by three ophthalmologists (SK, MKK, and CHY) who specialized in DED. The median score of each zone was chosen as the ground truth. If the scores of all three ophthalmologists were different or if the score difference between one and the other ophthalmologists was more than 1, the ground truth NEI score was determined through a consensus meeting. A total of 1294 anterior segment photograph images (1100 for grading system establishment, 94 images for external validation, 100 images for serial data analysis) were reviewed.

### Development of the fully automated grading system

**Fig 2:**
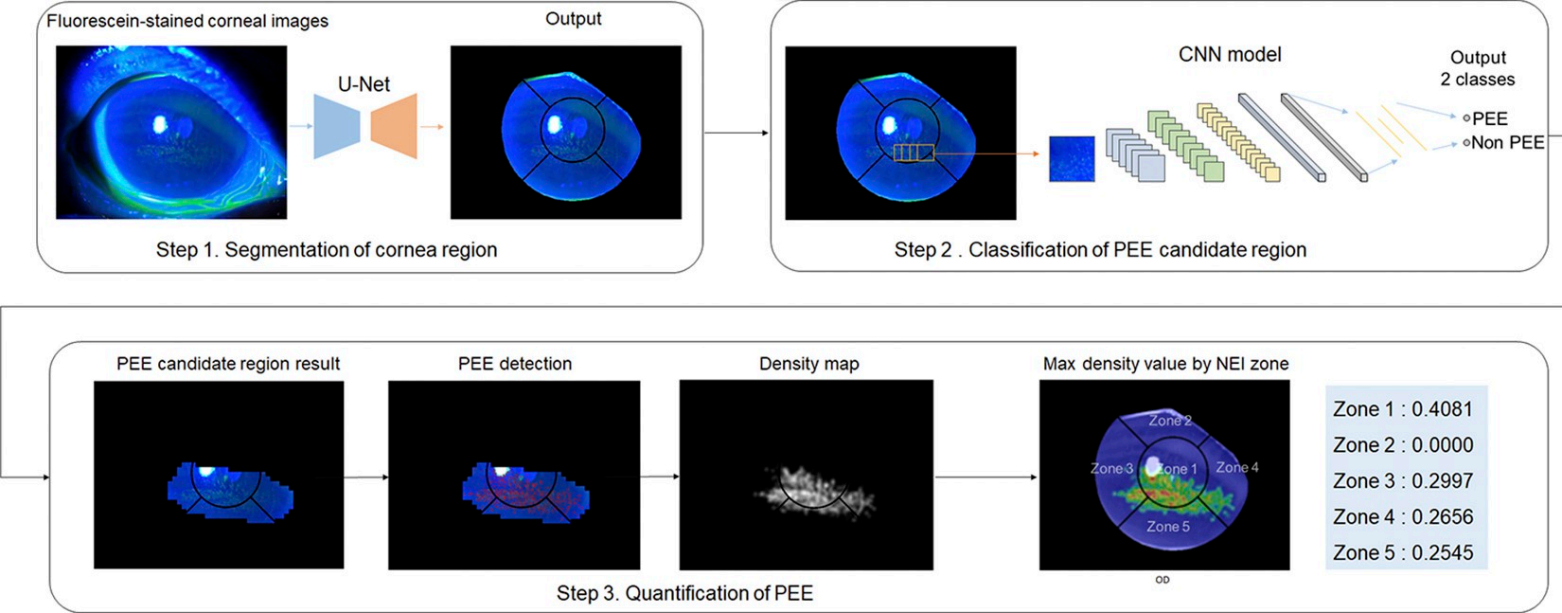
Diagram of the proposed deep learning system. In step 1, corneal region in fluorescein-stained slit lamp image is segmented using U-Net architecture with 1100 images and their corneal region labeled masks. In step 2, CNN-based classification model was trained with 200 images and their PEE and non-PEE labeled data to find the PEE candidate regions within the corneal region. In step 3, PEE quantification is performed using PEE density map and presented as MDV. PEE, punctate epithelial erosion; MDV, maximum density value.

The fully automated grading system for DED severity was developed using three steps: (1) corneal segmentation, (2) classification of CFS candidate regions, and (3) estimation of NEI grades within the CFS candidate regions (Fig 2).

**Fig 3:**
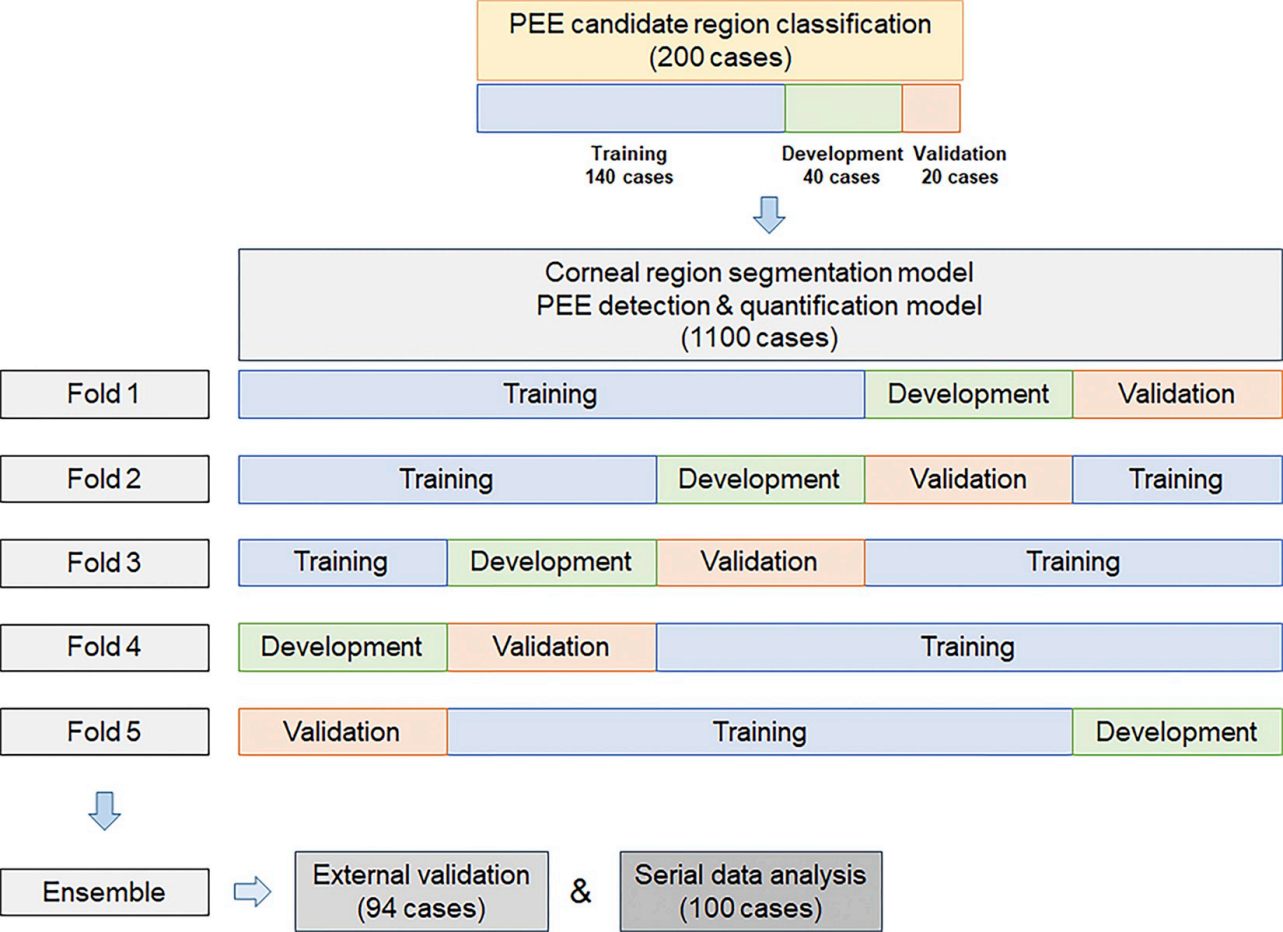
Schematic diagram of dataset splitting for deep learning analysis. For the PEE candidate regional model, 200 cases were divided into the training, development, and validation sets in a 7:2:1 ratio. For the corneal region segmentation model, PEE detection, and quantification model, 1100 cases were divided 5-folds. Also, data from 94 cases were used for external validation of the entire system, and data from another 100 cases were used for serial data analysis. PEE, punctate epithelial erosion.

Corneal segmentation model established as first step in fully automated grading system. The outer circles of the grid used to evaluate the NEI score were used as the ground truth for the corneal contour. In cases where the margin of the grid was partially covered with the eyelid, The outer contour of the cornea was manually drawn and used as the ground truth. For cross-validation, 1100 fluorescein-stained corneal images from 1100 patients were divided into the training, development, and validation sets as stratified five folds (Fig 3). Before training, all images were pre-processed using two steps: 1) normalization was performed to change the range of image intensities from 0–255 to 0–1 and 2) contrast-limited adaptive histogram equalization was used to improve the contrast of the images [21]. All input images were resized to 512 x 512 pixels using zero-padding. The U-Net architecture demonstrates outstanding performance in medical image segmentation and is used to establish a segmentation model [22]. Corneal regions in the 1100 anterior segment images were segmented using U-Net architecture with corneal region labeled masks. In this study, sigmoid function was employed as the activation function and Dice coefficient was adopted with stochastic gradient descent (SGD) optimizer to optimize the model’s parameters [23–25]. Dice coefficient is an index used to evaluate the similarity between two areas. Dice coefficient is calculated by doubling the size of the shared area and dividing by the sum of the sizes of the two areas. The performance of the model was evaluated using the average Dice coefficient of each fold. The predicted image was post processed, and a grid was drawn to obtain a mask. A circle is detected in the predicted image using the Hough circle transform algorithm [26]. It removes the predicted image corresponding to the outer space of the corresponding circle and then complements the shape with the convex hull algorithm. An inner grid was drawn to divide the five zones of the corneal region. In the previous process, the ratio of the inner grid circle was 1:1.6:1. The central ratio was the diameter of Zone 1. This value was used to separate Zone 1 by drawing a circle in the center. Lines were drawn at 45° and 135° to distinguish the rest of the zone based on the horizontal line passing through the origin. The majority-voting ensemble method was used to integrate the results of the entire system.

**Fig 4:**
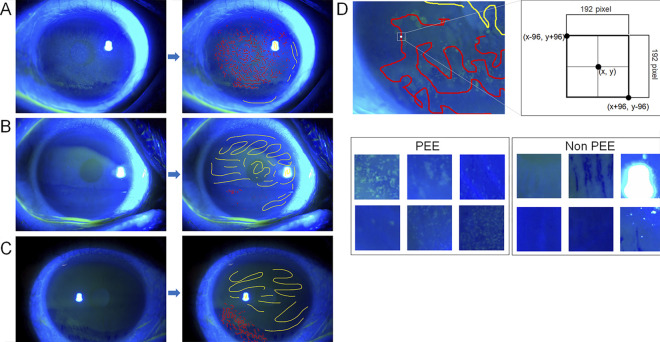
Examples of PEE and non-PEE labeling generated using Microsoft paint software. (A–C). Labeling of definite PEE (red color) and definite non-PEE (yellow color). (D). Extraction of patches sized 192 × 192 pixels for learning. PEE, punctate epithelial erosion.

In the second step, to reduce false-positive regions that can mimic PEE, such as flashlights or filaments, the CNN-based classification model was used to find the areas where PEE may exist within the corneal region segmented in step 1. To train the classification model, 200 images from 200 patients were divided in a 7:2:1 ratio into the training, development, and validation sets. The 200 images were labeled with red (definite PEE region) and yellow (definite non-PEE region) (Fig 4A–4C). Then, after extracting the coordinates of red and yellow labeling from the images, 100–150 coordinates were randomly selected to set the center. Calculation of extracted patches measuring 192×192 pixels [(x, y), (x-96, y-96), (x+96, y+96)] was conducted (Fig 4D). A total of 24559 patches were used for the learning. Among several classification models (VGG16 [27], VGG19 [27], and InceptionV3 [28]), the ImageNet [29] pre-trained VGG16 showed the best performance and was thus adopted in this model. While U-Net can identify PEE candidate regions, the fully convolutional network (FCN) model was chosen due to the need for more detailed labeling of the PEE region and considering the resolution of the images. FCN model was trained with 200 image and PEE and non-PEE label data to find the PEE candidate areas within the corneal region. During training, an SGD optimizer with a weighted decay of 1e-6 and a batch size of 64 was used. For inferences, 192 × 192 pixel-sized patches were overlapped by 25% using a sliding window to extract the results. Additionally, we verified the model using the Class Activation Map (CAM) to evaluate whether the model effectively classifies PEE regions. In CAM, the output of the final convolutional layer was utilized as feature maps. Global average pooling was then employed to calculate a weighted average for each class, and softmax was applied to obtain the pixel probabilities of specific classes [30].

In step 3, because PEE showed a blob in the image, the blob detection algorithm was used to detect PEE in the CFS candidate region extracted in step 2. Blob algorithm detected the areas of digital images with different characteristics such as brightness or color from surrounding areas. Given that PEE appeared green in the standard image, the algorithms were run using exclusive green channels. After PEE detection, the density was calculated by sliding and overlapping the 32 × 32 window by 50%, with consideration of the PEE size. After calculation, the density map was resized to match the original image size. The largest density value, named the maximum density value (MDV), was extracted for each zone, and the representative characteristics of each zone were determined. CFS score by NEI scale is evaluated based on the highest density part of PEEs of each zone. Therefore, we evaluated the MDV of each zone to estimate PEE grading. As in the corneal segmentation model, 1100 fluorescein-stained corneal images were divided into the training, development, and validation sets as stratified five folds (Fig 3).

### External validation

External validation was performed using 94 images from Hospital 2. All images were graded on the NEI scale by three ophthalmologists, as in the hospital 1 data, and the correlation between the prediction value and ground truth was evaluated.

### Serial data analysis

Two images of the same eye taken on different days were compared in every 50 patients (100 images in total). To evaluate the accuracy in predicting aggravation or improvement of DED, changes in MDV on different days in the same eye were compared with those in the ground truth. Aggravation of DED was defined as an increase in NEI score, and improvement of DED was defined as a decrease in NEI score.

### Statistical analysis

Model performance was validated according to Spearman correlation (*r*), which was used to evaluate the correlation between the model output and the ground truth. All statistical analyses were conducted using the GraphPad Prism 9.4.1 software (GraphPad Inc., San Diego, CA, USA).

## Results

### Patient and image characteristics

**Fig 5:**
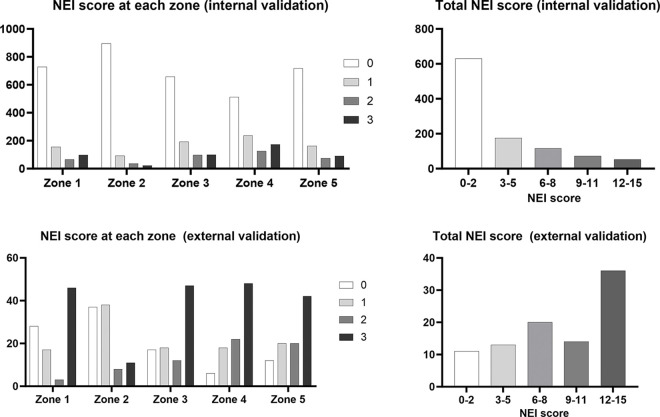
Ground truth NEI score of the development dataset (hospital 1 data) at each zone (A) and total NEI score (B), and ground truth NEI score of the external validation dataset (hospital 2 data) at each zone (C) and total NEI score (D). NEI, National Eye Institute.

Among the 1100 patients in the development set (1400 images from hospital 1), 113 (10.3%) and 15 (1.4%) patients had Sjögren syndrome and ocular GVHD, respectively (Table 1). Meanwhile, in the external validation set (94 images from hospital 2), 65 patients (69.1%) were previously diagnosed with Sjögren syndrome. The distribution of the ground truth NEI score in each zone and the ground truth total NEI score in hospitals 1 and 2 are shown in Fig 5.

**Table 1:** Diagnosis of the patients in the development (hospital 1) and external validation (hospital 2) datasets.

Diagnosis	Hospital 1 (N = 1100)	Hospital 2 (N = 94)
Sjögren syndrome, n (%)	113 (10.3%)	65 (69.1%)
Ocular GVHD, n (%)	15 (1.4%)	0 (0%)
Others, n (%)	972 (88.4%)	29 (30.9%)

GVHD, graft-versus-host disease

**Fig 6:**
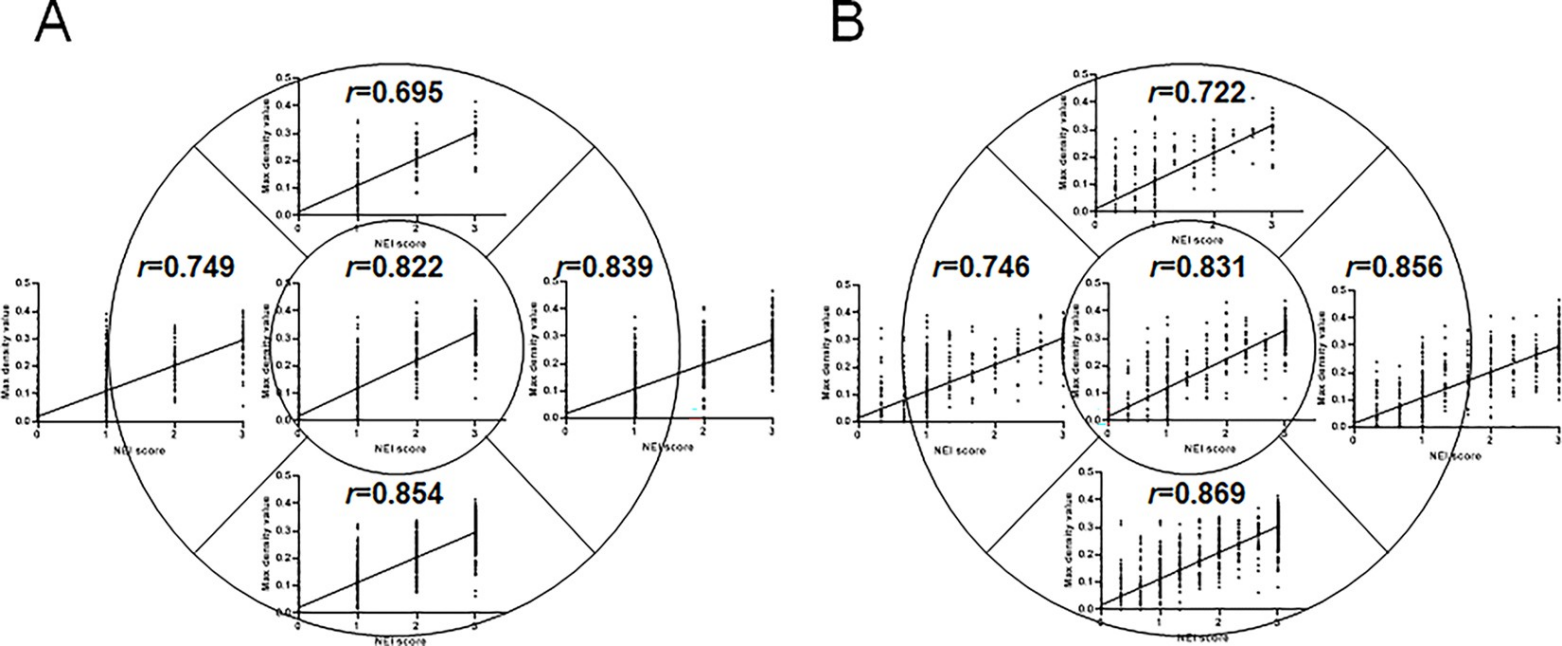
(A) Correlation between median NEI score and MDV, and (B) correlation between mean NEI score and MDV by zone. NEI, National Eye Institute; MDV, maximum density value.

Table 2 shows the clinical score agreement among the three investigators before consensus meeting for ground truth. The Spearman correlation coefficients were 0.878, 0.885, and 0.920. After the consensus meeting, Spearman correlation coefficients among the investigators were raised to 0.905, 0.903, and 0.934 (S1 Table). The correlation between maximum density value (MDV) and median/mean NEI score determined by the three investigators is shown in Fig 6. Compared with the median NEI score, the mean NEI score showed higher correlation with MDV in most zones (Fig 6). The correlation between MDV and both median (*r* = 0.854) and mean (*r =* 0.869) NEI scores was the highest in zone 5. Meanwhile, the correlation between MDV and NEI score was the lowest in zone 2.

**Table 2:** Agreement in corneal fluorescein staining (CFS) scores based on the National Eye Institute (NEI) scale.

CFS score by NEI scale	Investigator 1	Investigator 1	Investigator 2
	vs. Investigator 2	vs. Investigator 3	vs. Investigator 3
Spearman correlation coefficient	0.878	0.885	0.920
(95% CI)	(0.864–0.892)	(0.871–0.897)	(0.910–0.929)
Mean difference in Bland-Altman	-0.310	-0.385	-0.075
(P value)	(<0.001)*	(<0.001)*	(0.424)
SD Difference	0.746	0.688	0.598

* Differences between investigators are significantly greater than zero (P < 0.05).

CI, confidence interval; SD, standard deviation

### Results of segmentation and classification model

**Fig 7:**
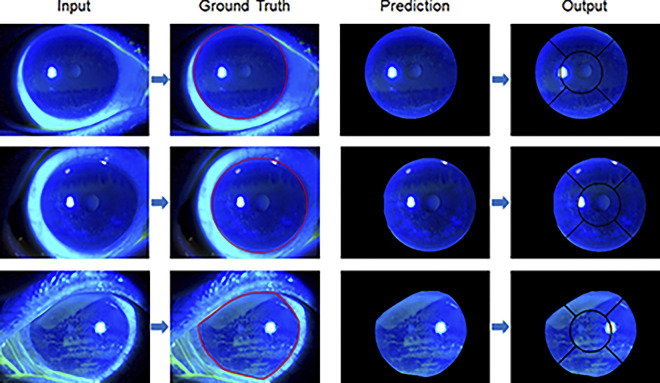
Examples of the training dataset, input, and final output used in the corneal segmentation step. The input is a fluorescein-stained corneal image (first panel). The ground truth (second panel) is used to train the cornea segmentation model. The red lines in the second panel indicate corneal regions. The third panel displays the predicted image of corneal segmentation. The final output (last panel) is a grid mask on a predictive image obtained using computer vision algorithms.

The examples of corneal segmentation inputs, prediction results, and outputs are shown in Fig 7. The model generates an output of 512 × 512 pixels, and after the removal of padding, this output was resized to the original image of 2576 × 1934 pixels. The Dice coefficients for corneal segmentation were higher than 0.96 in all 5-fold data, and the average Dice coefficient was 0.962 ± 0.001.

To reduce false positives mimicking PEE, threshold was set at 0.98 for highly specific model tuning. At a threshold of 0.98, the classification model achieved an accuracy of 0.89, a sensitivity of 0.82, a specificity of 0.96, and AUC of 0.97, indicating its robust performance.

**Fig 8:**
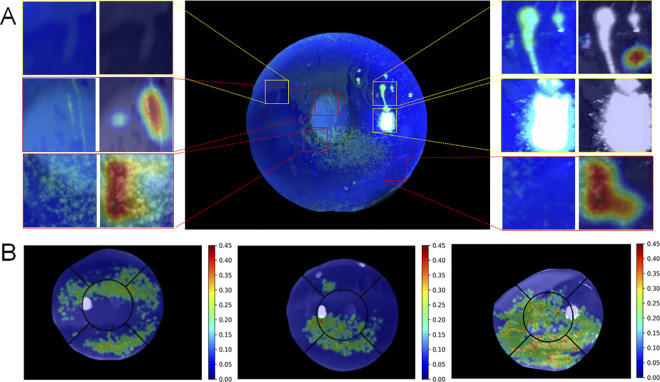
Illustration of classification model and density map results. (A) CAM of PEE candidate region classification. The red and yellow boxes represent true positive and true negative of the PEE classification model, respectively. (B) Density map results. Blue indicates low PEE density, and red indicates high PEE density. CAM, class activation map; PEE, punctate epithelial erosion.

CAM is shown in Fig 8A. The red boxes indicate the patches containing PEE (true positive). The yellow boxes indicate non-PEE patches (true negative), such as flashlights, filaments, and reflection light due to tear film. As shown in the yellow box in Fig 8A, the CAM focused on a portion of the region but correctly classified it as true negative. Fig 8B shows the result of the overlapped density map on the original images; low severity is shown in green and high severity is shown in red.

### Internal and external validations

**Fig 9:**
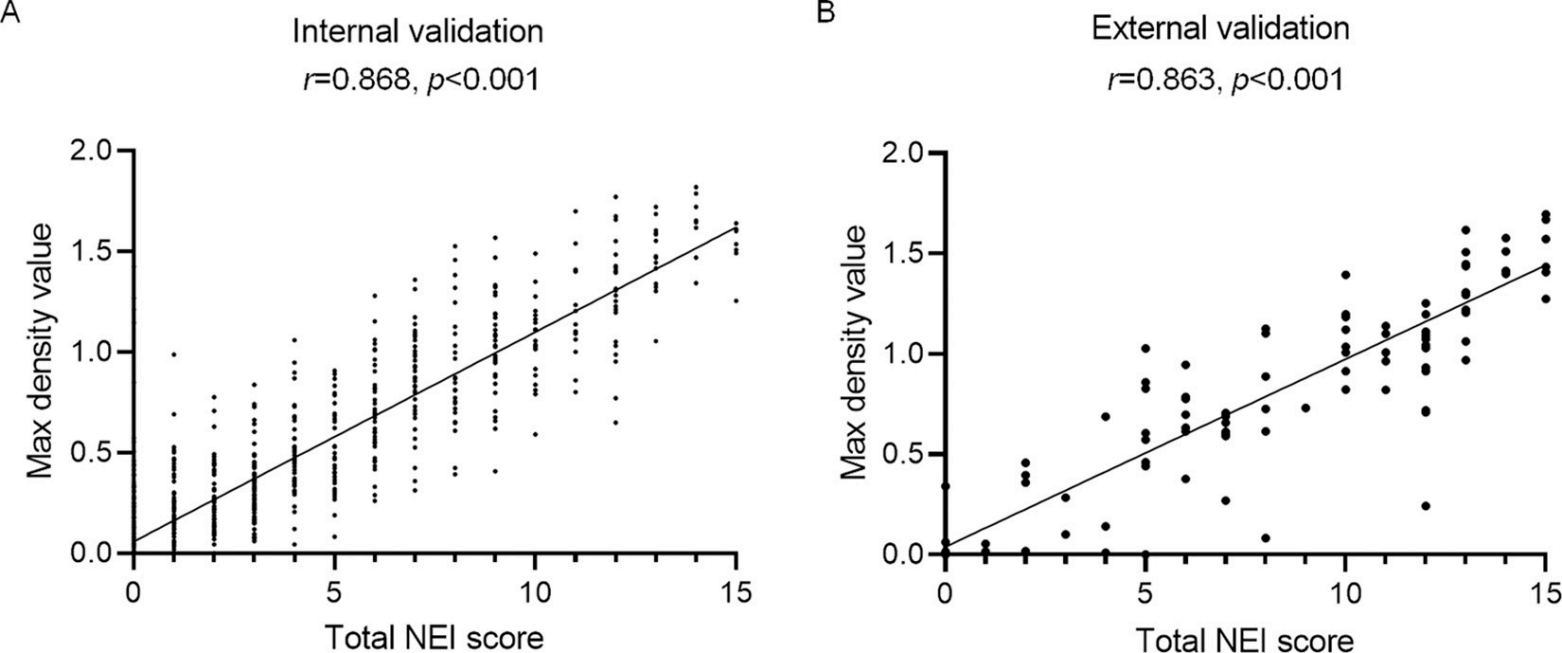
Correlation between total NEI score and MDV. (A) Spearman correlation result between total NEI score and MDV of the development dataset (hospital 1). The Spearman correlation is 0.868 (p<0.001). (B) Spearman correlation results between the total NEI score and MDV of external validation dataset (hospital 2). The Spearman correlation coefficient is 0.863, and the p-value is <0.001. NEI, National Eye Institute; MDV, maximum density value.

The Spearman correlation between MDV and ground truth NEI score was 0.868 in the internal validation datasets (Fig 9A). The Spearman correlation between MDV and ground truth NEI score was 0.863 in the external validation dataset (Fig 9B).

### Serial data analysis

The agreement between the proposed model and ground truth measures for improvement or deterioration was consistent in 44 of 50 patients. The six patients showed different directions in the prediction of the model and ground truth (Table 3). Of the 6 patients with discrepancy between model’s predictions and ground truth, the degree of improvement or deterioration in ground truth NEI score was 2 in four patients and 1 in two patients.

**Table 3:** Agreement between the entire model and ground truth data for the assessment of improvement or deterioration in 50 eyes (n = 100 images).

		Model	
		Improvement	Deterioration
Ground truth	Improvement	19	2
	Deterioration	4	25

## Discussion

In this study, a fully automated artificial intelligence system calculating NEI scores by fluorescein-stained corneal images was developed. The system automatically segmented the corneal region and identified the PEE candidate region. The score of the densest region of each area was calculated according to the NEI scoring system. The total corneal area score calculated by the automated AI system showed a high correlation with the ground-truth NEI score (*r* = 0.868), and this was comparable to the correlation with the NEI scale score determined by ophthalmologists (*r* = 0.878–0.920). This system successfully predicted 88% of disease improvement or deterioration.

Unlike the Oxford scale or Sjögren’s International Collaborative Clinical Alliance ocular staining score which evaluates the entire cornea without segmentation, the NEI scoring system divides the cornea into five regions using a grid [13, 31, 32]. The corneal segmenting grid from the 1995 NEI workshop is arbitrary [13], and subsequent studies using the NEI scoring system did not mention the setting of the ratio of the circle [18, 19]. Therefore, the grid was used as in the study of Amparo et al. [16] because they reported that the NEI score may vary depending on the proportion of the inner grid and suggested that a certain ratio of circles should be set.

Two studies previously applied deep learning models to predict the Oxford scale score [17, 33]. In one study, the region with PEE was extracted, and the ratio in the entire cornea was calculated. The correlation with ground truth was 0.85 [33]. In the other study, the Oxford scale score was calculated using a formula after measuring the number of PEEs, and the correlation with the ground truth was 0.981 [17]. Feng et al. reported an automated dry eye grading system based on topological features to predict the ocular staining score recommended by Sjögren’s International Collaborative Clinical Alliance [34]. The authors utilized image processing techniques to extract topological and morphological features from corneal images, and subsequently analyzed and classified them using machine learning models [34]. However, these previous methods could not be used for calculating the NEI score because of the complexity of the NEI scale. Therefore, the corneal regions were divided as in the method presented for the NEI scoring system, scored the densest PEE region in each region, and combined the scores for each region.

Qu et al. reported an automated NEI grading system using deep learning [19]. A staining grading model predicting a score from 0 to 3 for each region was trained with images also scored from 0 to 3 [19]. However, our system adopted a method to quantify PEEs and obtain the MDV. This is more similar to the method of the NEI scoring system and may thus be used as an objective value. Additionally, our proposed method has an advantage that closely aligns with the actual clinical practice of step-by-step review as in the same process of CFS scoring in a clinical setting. This process includes segmenting the corneal region into 5 zones, evaluating the PEE density in each zone, and then summing the evaluations to calculate a total score, which distinguishing it from existing end-to-end deep learning methods. PEE quantification involves two steps of false-positive reduction: cornea segmentation and PEE candidate region classification. Corneal segmentation involves segmentation of corneal regions and using these regions for scoring. This may reduce false positives by eliminating the possibility of detecting in areas outside the target scoring region. The NEI score can then be further explained according to the final density map of PEE.

Although the external dataset included a higher proportion of patients with Sjögren syndrome and patients with more severe disease, external validation in this study showed a Spearman correlation of 0.863, similar with the 0.868 in the internal validation. This indicates that the reliability and the performance of the developed system can be universally applied in settings with different DED severities.

This study has some limitations. First, there were some images in which the peripheral cornea was partially occluded by the upper eyelid. This was because the Korean patients frequently showed low palpebral fissure height. This might have caused the lowest correlation between MDV and NEI score in zone 2.

Second, although one eye was included only once, both eyes of some patients were included for grading system establishment in this study. Third, several outlier cases showed a low correlation between the prediction value and the ground truth. There may have been misdetections of tear break up, low contrast, dense PEE, segmentation grid edge, and light reflex. The internal dataset included 16 cases of misdetection: 6 cases of tear break-up, 4 cases of low contrast, 4 cases of dense PEE, 1 case of segmentation grid edge, and 1 case of light reflex. Meanwhile, the external dataset included 7 cases of misdetection: low contrast (2), tear break-up (2), dense PEE (1), segmentation grid edge (1), and light reflex misdetection (1). Third, the images used in model training were obtained from a single institution. The dataset used for model training should include images from multiple institutions to ensure model reproducibility and consistency in results. In addition, previous study [35] indicated that the use of yellow cut-off filters to remove external blue light is critical for the optimal visualization of ocular surface staining and improve the sensitivity and specificity of diagnosis. Therefore, to improve the performance of the proposed system, training the corneal segmentation model and PEE candidate region classifier using a yellow cutoff filter should be further studied. Also, this study was conducted in a single institution with same camera. Multicenter study is needed to further validate the grading system. Furthermore, extracting PEE candidate region can exclude some area where PEE might exist. Previous studies [19, 20] regarding the evaluation of PEE also have the same limitations. In clinical practice, it is difficult to calculate the NEI score by applying a constant grid using only slit-lamp examination. Thus, despite these limitations, the current system has clinical value because it can automatically apply a grid to the cornea and calculate scores by simply inputting fluorescein-stained corneal images into the system. Particularly, this system can be applied to dry eye clinical trials where objective measurement is important or to multicenter studies that need to reduce interobserver variation.

In conclusion, a fully automated deep learning-based grading system for dry eye severity was developed and was able to evaluate the CFS score with accuracy as high as that of expert ophthalmologists. With this system, a more reliable and reproducible method for DED severity grading can be achieved. In addition, this system can be used in the future to reduce human error and in clinical trials or multicenter studies.

## Supporting information

S1 Table Agreement of CFS score based on NEI scale among investigators after consensus meeting. (DOCX)

S1 Dataset (ZIP)

S2 Dataset (ZIP)

S3 Dataset (ZIP)
