# Supplementary material for: Deep learning-based fully automated grading system for dry eye disease severity
Source: PLoS One. 2024 Mar 14;19(3):e0299776. doi: 10.1371/journal.pone.0299776 (PMC10939279; doi:10.1371/journal.pone.0299776)
Supplement: S1 Table — (DOCX) [file pone.0299776.s001.docx]

**S1 Table. Agreement of corneal fluorescein staining (CFS) score based on National Eye Institute (NEI) scale among investigators after consensus meeting**

| CFS score by NEI scale | Investigator 1  vs. Investigator 2 | Investigator 1  vs. Investigator 3 | Investigator 2  vs. Investigator 3 |
| --- | --- | --- | --- |
| Spearman correlation coefficient  (95% CI) | 0.914  (0.904-0.924) | 0.930  (0.921-0.938) | 0.944  (0.937-0.950) |
| Mean difference in Bland-Altman  (P value) | -0.111  (0.135) | -0.370  (<0.001)* | -0.259  (<0.001)* |
| SD Difference | 0.538 | 0.469 | 0.483 |

* Differences between investigators are significantly greater than zero (P < 0.05).

CI, confidence interval; SD, standard deviation
