# Supplementary figures and images for: Deep learning-based fully automated grading system for dry eye disease severity (part 2 of 6)
Source: PLoS One. 2024 Mar 14;19(3):e0299776. doi: 10.1371/journal.pone.0299776 (PMC10939279; doi:10.1371/journal.pone.0299776)

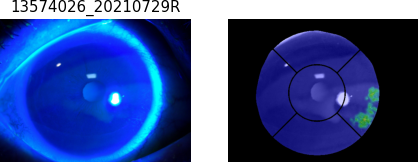

Supplement: S1 Dataset — (ZIP) [file pone.0299776.s002.zip › 13574026_20210729R/13574026_20210729R_whole.png]

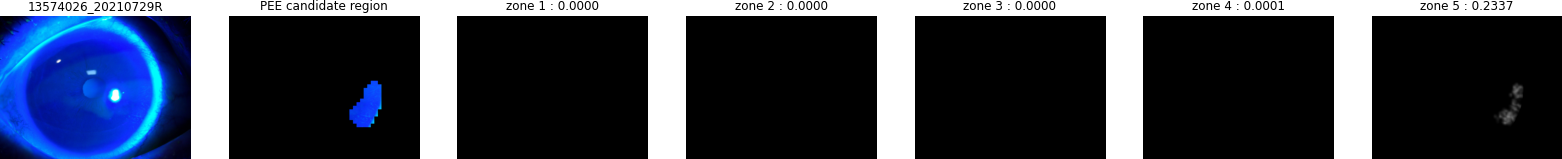

Supplement: S1 Dataset — (ZIP) [file pone.0299776.s002.zip › 13574026_20210729R/13574026_20210729R_zone.png]

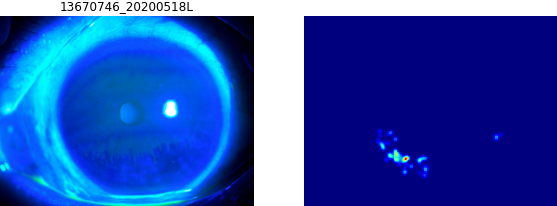

Supplement: S1 Dataset — (ZIP) [file pone.0299776.s002.zip › 13670746_20200518L/13670746_20200518L_densitymap.png]

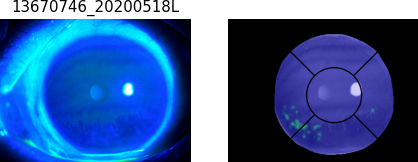

Supplement: S1 Dataset — (ZIP) [file pone.0299776.s002.zip › 13670746_20200518L/13670746_20200518L_whole.png]

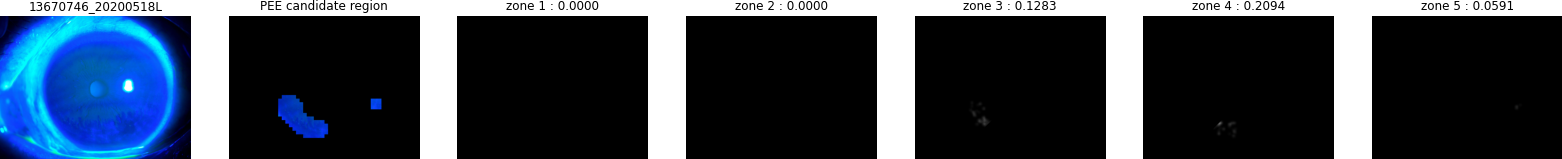

Supplement: S1 Dataset — (ZIP) [file pone.0299776.s002.zip › 13670746_20200518L/13670746_20200518L_zone.png]

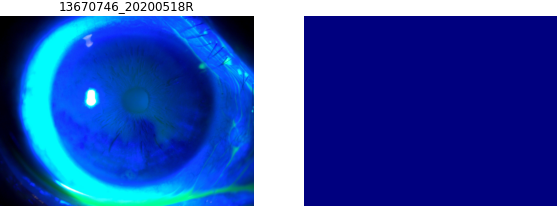

Supplement: S1 Dataset — (ZIP) [file pone.0299776.s002.zip › 13670746_20200518R/13670746_20200518R_densitymap.png]

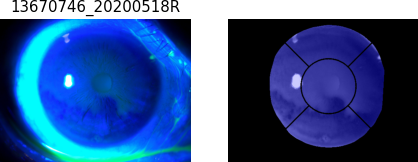

Supplement: S1 Dataset — (ZIP) [file pone.0299776.s002.zip › 13670746_20200518R/13670746_20200518R_whole.png]

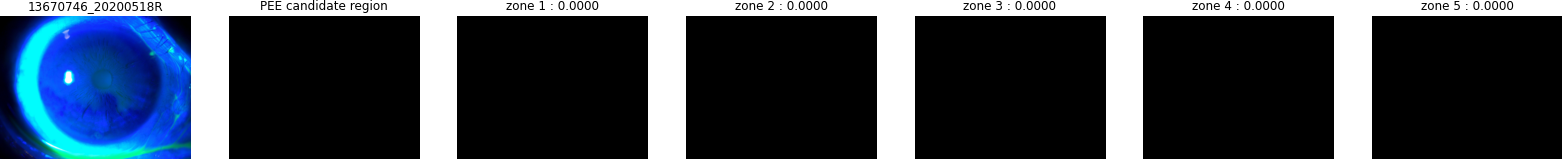

Supplement: S1 Dataset — (ZIP) [file pone.0299776.s002.zip › 13670746_20200518R/13670746_20200518R_zone.png]

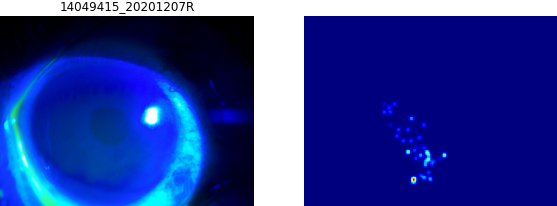

Supplement: S1 Dataset — (ZIP) [file pone.0299776.s002.zip › 14049415_20201207R/14049415_20201207R_densitymap.png]

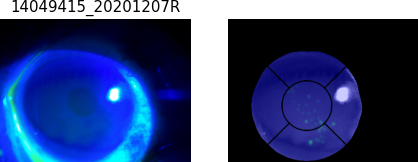

Supplement: S1 Dataset — (ZIP) [file pone.0299776.s002.zip › 14049415_20201207R/14049415_20201207R_whole.png]

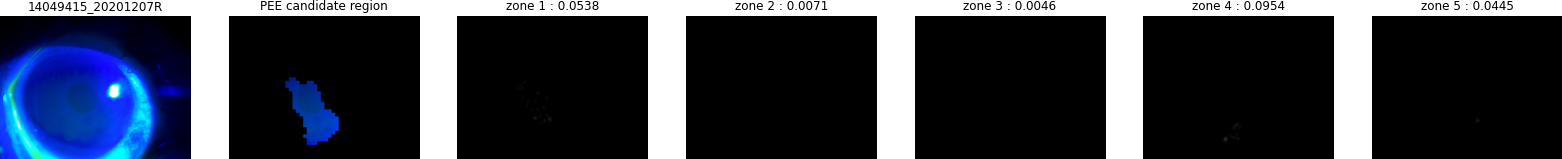

Supplement: S1 Dataset — (ZIP) [file pone.0299776.s002.zip › 14049415_20201207R/14049415_20201207R_zone.png]

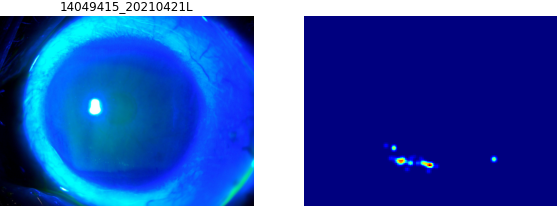

Supplement: S1 Dataset — (ZIP) [file pone.0299776.s002.zip › 14049415_20210421L/14049415_20210421L_densitymap.png]

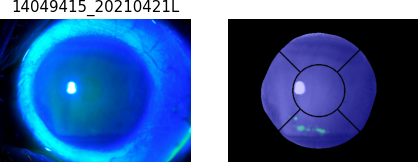

Supplement: S1 Dataset — (ZIP) [file pone.0299776.s002.zip › 14049415_20210421L/14049415_20210421L_whole.png]

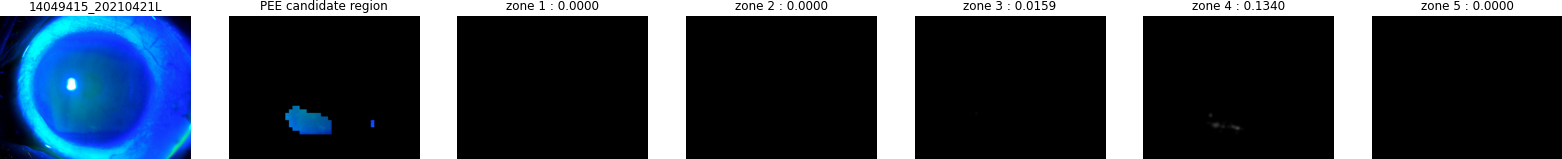

Supplement: S1 Dataset — (ZIP) [file pone.0299776.s002.zip › 14049415_20210421L/14049415_20210421L_zone.png]

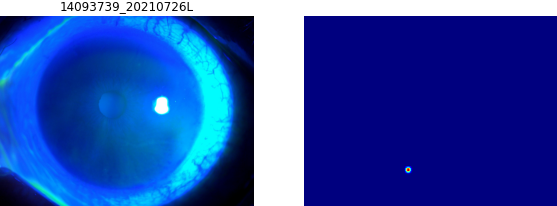

Supplement: S1 Dataset — (ZIP) [file pone.0299776.s002.zip › 14093739_20210726L/14093739_20210726L_densitymap.png]

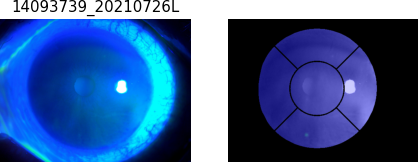

Supplement: S1 Dataset — (ZIP) [file pone.0299776.s002.zip › 14093739_20210726L/14093739_20210726L_whole.png]

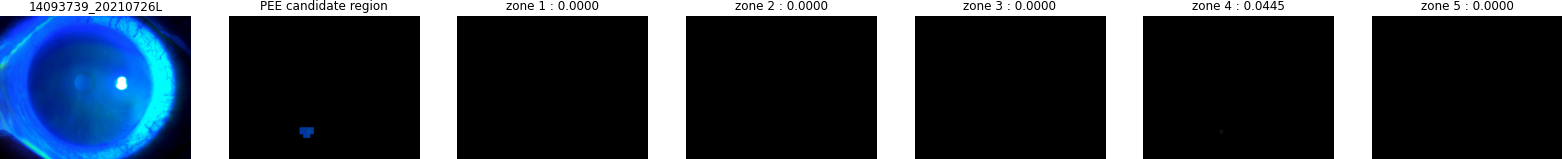

Supplement: S1 Dataset — (ZIP) [file pone.0299776.s002.zip › 14093739_20210726L/14093739_20210726L_zone.png]

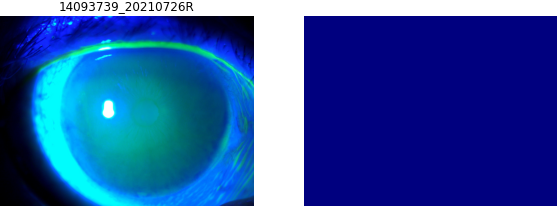

Supplement: S1 Dataset — (ZIP) [file pone.0299776.s002.zip › 14093739_20210726R/14093739_20210726R_densitymap.png]

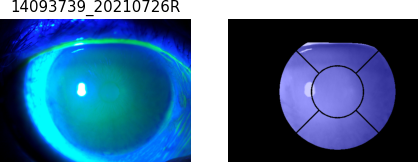

Supplement: S1 Dataset — (ZIP) [file pone.0299776.s002.zip › 14093739_20210726R/14093739_20210726R_whole.png]

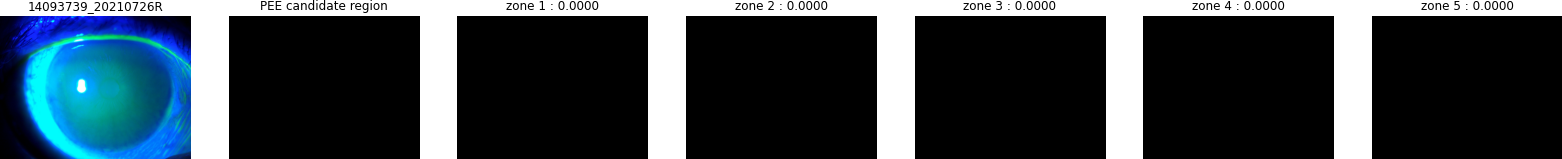

Supplement: S1 Dataset — (ZIP) [file pone.0299776.s002.zip › 14093739_20210726R/14093739_20210726R_zone.png]

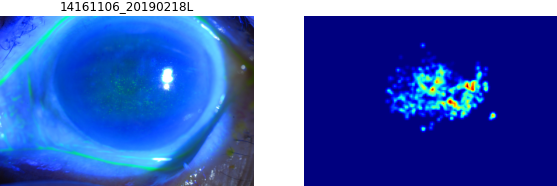

Supplement: S1 Dataset — (ZIP) [file pone.0299776.s002.zip › 14161106_20190218L/14161106_20190218L_densitymap.png]

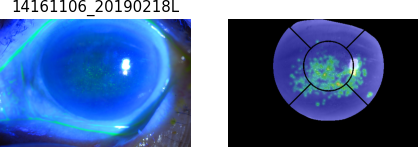

Supplement: S1 Dataset — (ZIP) [file pone.0299776.s002.zip › 14161106_20190218L/14161106_20190218L_whole.png]

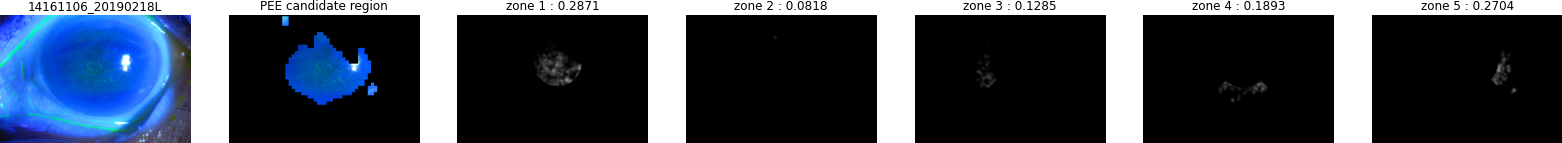

Supplement: S1 Dataset — (ZIP) [file pone.0299776.s002.zip › 14161106_20190218L/14161106_20190218L_zone.png]

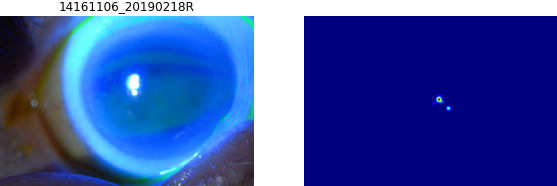

Supplement: S1 Dataset — (ZIP) [file pone.0299776.s002.zip › 14161106_20190218R/14161106_20190218R_densitymap.png]

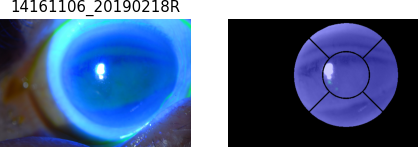

Supplement: S1 Dataset — (ZIP) [file pone.0299776.s002.zip › 14161106_20190218R/14161106_20190218R_whole.png]

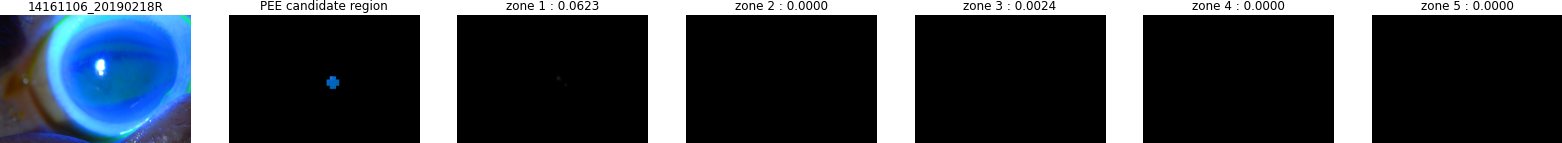

Supplement: S1 Dataset — (ZIP) [file pone.0299776.s002.zip › 14161106_20190218R/14161106_20190218R_zone.png]

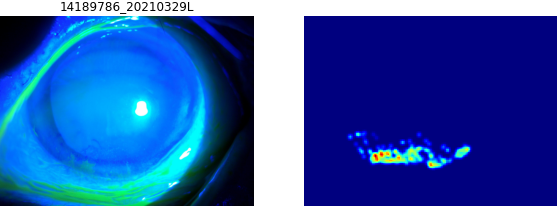

Supplement: S1 Dataset — (ZIP) [file pone.0299776.s002.zip › 14189786_20210329L/14189786_20210329L_densitymap.png]

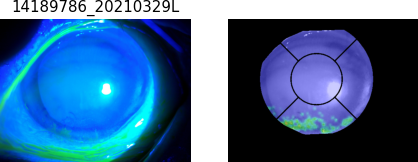

Supplement: S1 Dataset — (ZIP) [file pone.0299776.s002.zip › 14189786_20210329L/14189786_20210329L_whole.png]

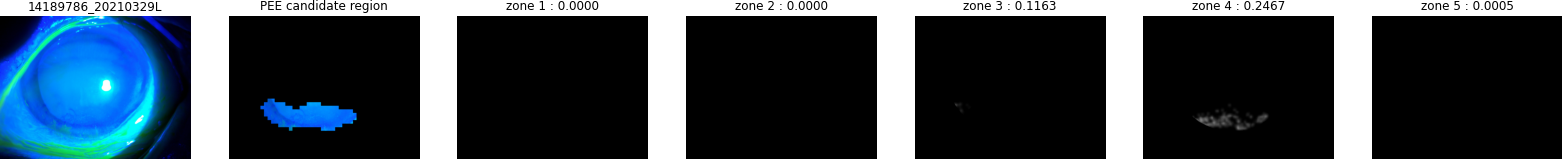

Supplement: S1 Dataset — (ZIP) [file pone.0299776.s002.zip › 14189786_20210329L/14189786_20210329L_zone.png]

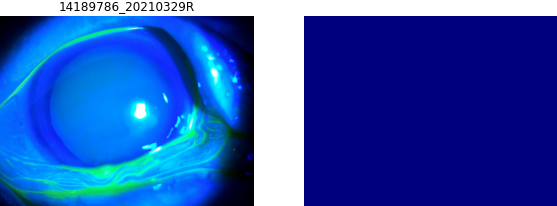

Supplement: S1 Dataset — (ZIP) [file pone.0299776.s002.zip › 14189786_20210329R/14189786_20210329R_densitymap.png]

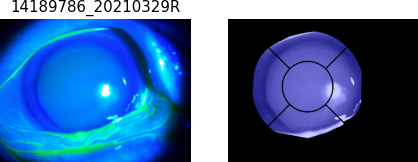

Supplement: S1 Dataset — (ZIP) [file pone.0299776.s002.zip › 14189786_20210329R/14189786_20210329R_whole.png]

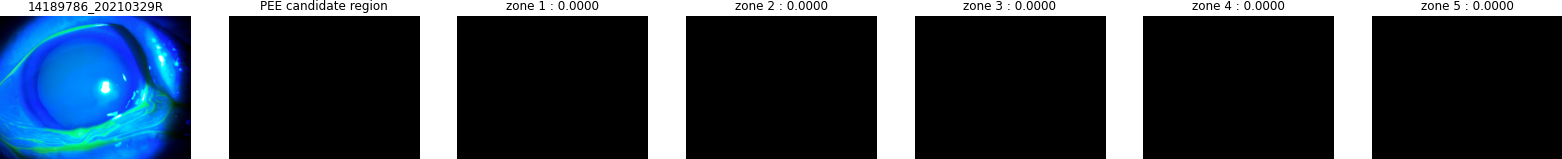

Supplement: S1 Dataset — (ZIP) [file pone.0299776.s002.zip › 14189786_20210329R/14189786_20210329R_zone.png]

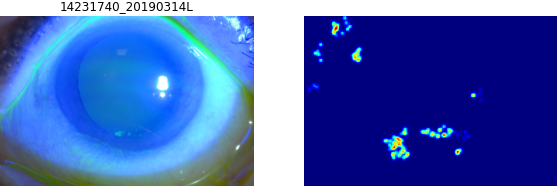

Supplement: S1 Dataset — (ZIP) [file pone.0299776.s002.zip › 14231740_20190314L/14231740_20190314L_densitymap.png]

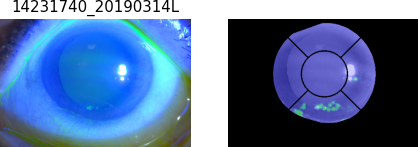

Supplement: S1 Dataset — (ZIP) [file pone.0299776.s002.zip › 14231740_20190314L/14231740_20190314L_whole.png]

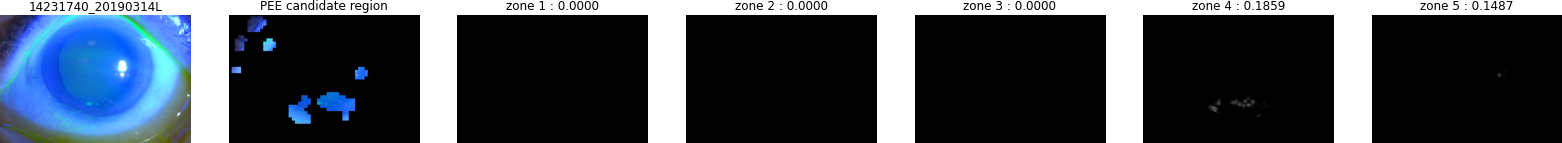

Supplement: S1 Dataset — (ZIP) [file pone.0299776.s002.zip › 14231740_20190314L/14231740_20190314L_zone.png]

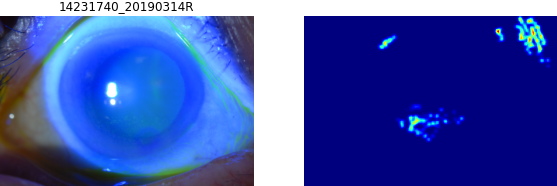

Supplement: S1 Dataset — (ZIP) [file pone.0299776.s002.zip › 14231740_20190314R/14231740_20190314R_densitymap.png]

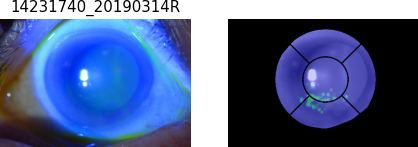

Supplement: S1 Dataset — (ZIP) [file pone.0299776.s002.zip › 14231740_20190314R/14231740_20190314R_whole.png]

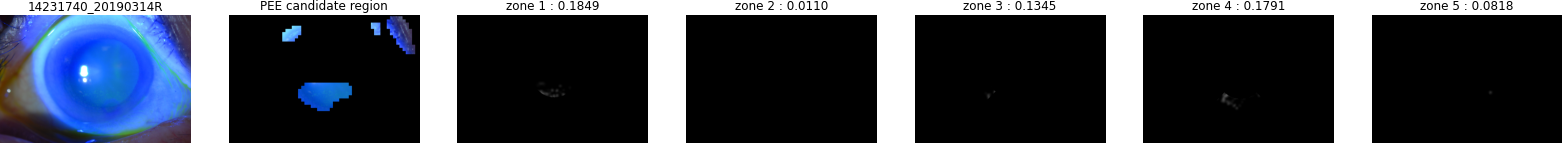

Supplement: S1 Dataset — (ZIP) [file pone.0299776.s002.zip › 14231740_20190314R/14231740_20190314R_zone.png]

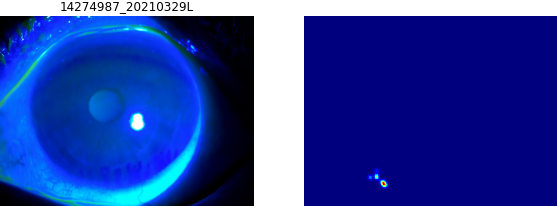

Supplement: S1 Dataset — (ZIP) [file pone.0299776.s002.zip › 14274987_20210329L/14274987_20210329L_densitymap.png]

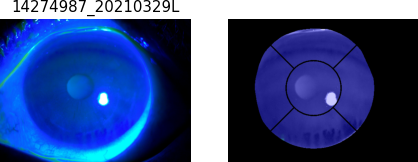

Supplement: S1 Dataset — (ZIP) [file pone.0299776.s002.zip › 14274987_20210329L/14274987_20210329L_whole.png]

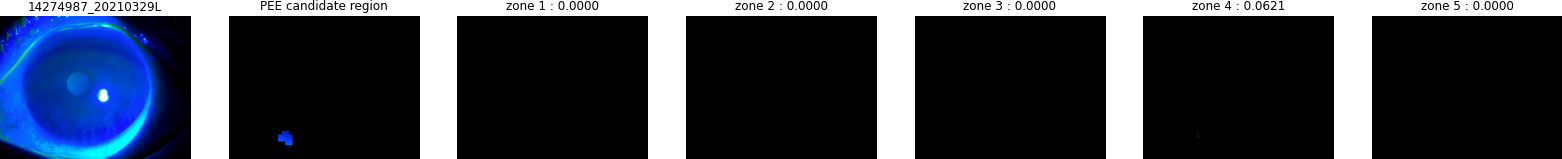

Supplement: S1 Dataset — (ZIP) [file pone.0299776.s002.zip › 14274987_20210329L/14274987_20210329L_zone.png]

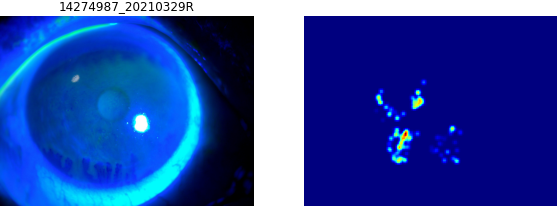

Supplement: S1 Dataset — (ZIP) [file pone.0299776.s002.zip › 14274987_20210329R/14274987_20210329R_densitymap.png]

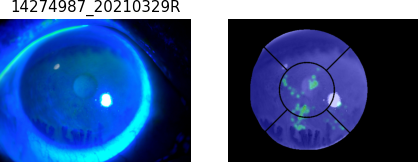

Supplement: S1 Dataset — (ZIP) [file pone.0299776.s002.zip › 14274987_20210329R/14274987_20210329R_whole.png]

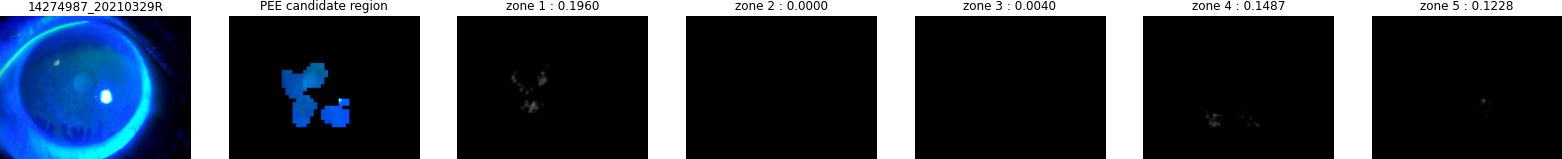

Supplement: S1 Dataset — (ZIP) [file pone.0299776.s002.zip › 14274987_20210329R/14274987_20210329R_zone.png]

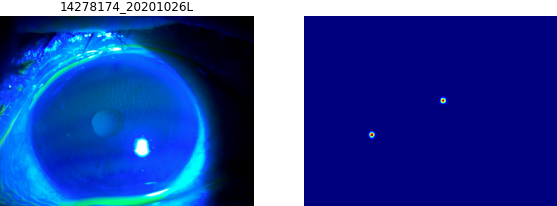

Supplement: S1 Dataset — (ZIP) [file pone.0299776.s002.zip › 14278174_20201026L/14278174_20201026L_densitymap.png]

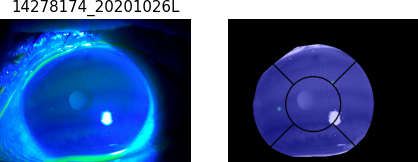

Supplement: S1 Dataset — (ZIP) [file pone.0299776.s002.zip › 14278174_20201026L/14278174_20201026L_whole.png]

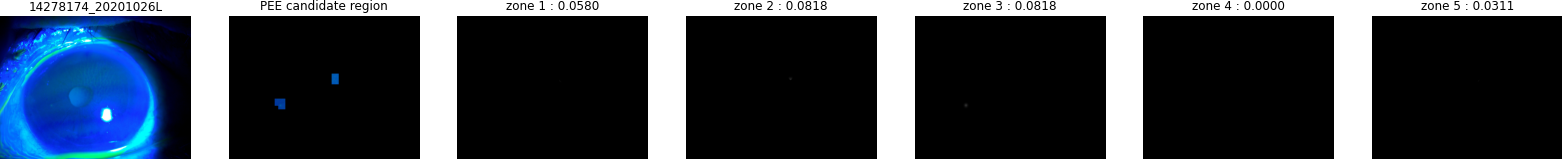

Supplement: S1 Dataset — (ZIP) [file pone.0299776.s002.zip › 14278174_20201026L/14278174_20201026L_zone.png]

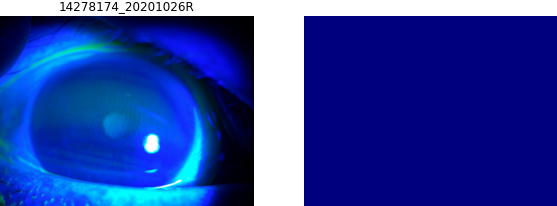

Supplement: S1 Dataset — (ZIP) [file pone.0299776.s002.zip › 14278174_20201026R/14278174_20201026R_densitymap.png]

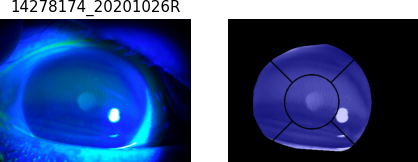

Supplement: S1 Dataset — (ZIP) [file pone.0299776.s002.zip › 14278174_20201026R/14278174_20201026R_whole.png]

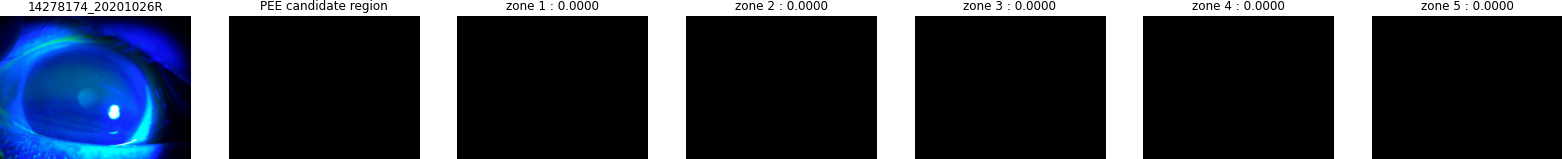

Supplement: S1 Dataset — (ZIP) [file pone.0299776.s002.zip › 14278174_20201026R/14278174_20201026R_zone.png]

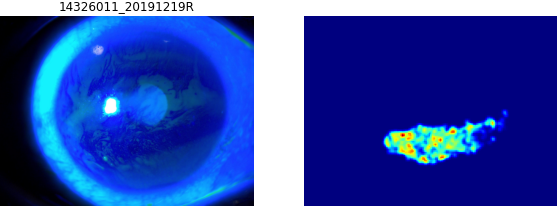

Supplement: S1 Dataset — (ZIP) [file pone.0299776.s002.zip › 14326011_20191219R/14326011_20191219R_densitymap.png]

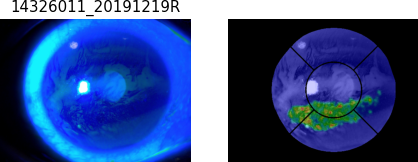

Supplement: S1 Dataset — (ZIP) [file pone.0299776.s002.zip › 14326011_20191219R/14326011_20191219R_whole.png]

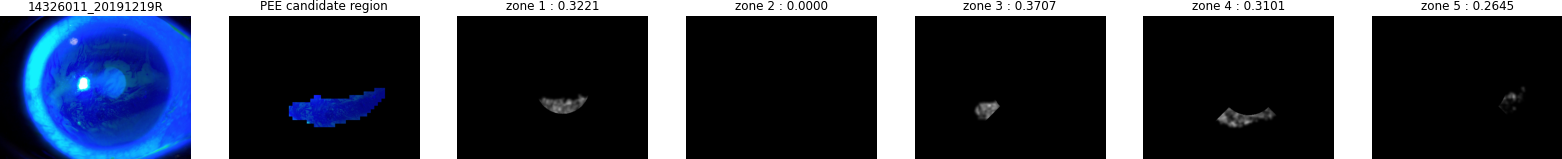

Supplement: S1 Dataset — (ZIP) [file pone.0299776.s002.zip › 14326011_20191219R/14326011_20191219R_zone.png]

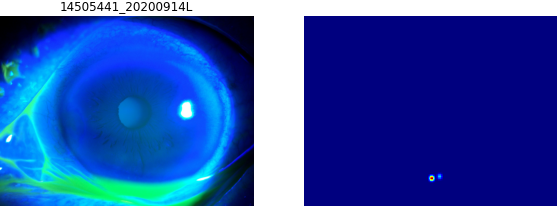

Supplement: S1 Dataset — (ZIP) [file pone.0299776.s002.zip › 14505441_20200914L/14505441_20200914L_densitymap.png]

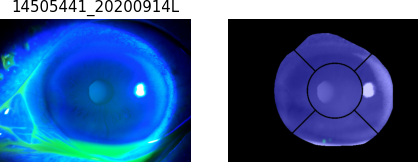

Supplement: S1 Dataset — (ZIP) [file pone.0299776.s002.zip › 14505441_20200914L/14505441_20200914L_whole.png]

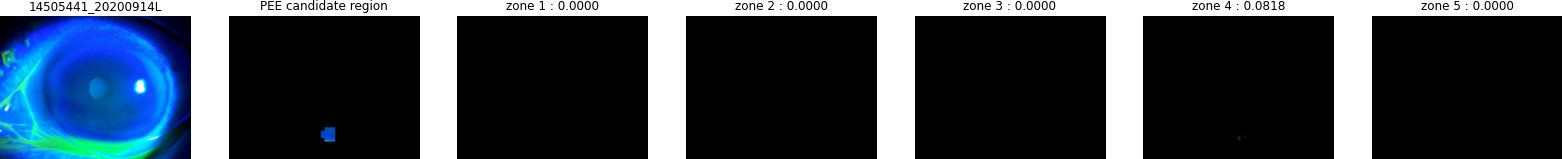

Supplement: S1 Dataset — (ZIP) [file pone.0299776.s002.zip › 14505441_20200914L/14505441_20200914L_zone.png]

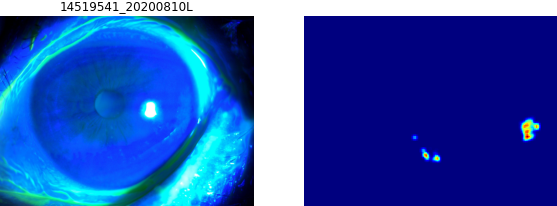

Supplement: S1 Dataset — (ZIP) [file pone.0299776.s002.zip › 14519541_20200810L/14519541_20200810L_densitymap.png]

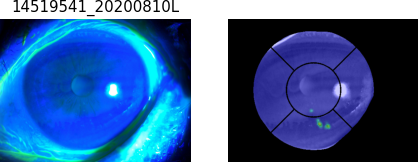

Supplement: S1 Dataset — (ZIP) [file pone.0299776.s002.zip › 14519541_20200810L/14519541_20200810L_whole.png]

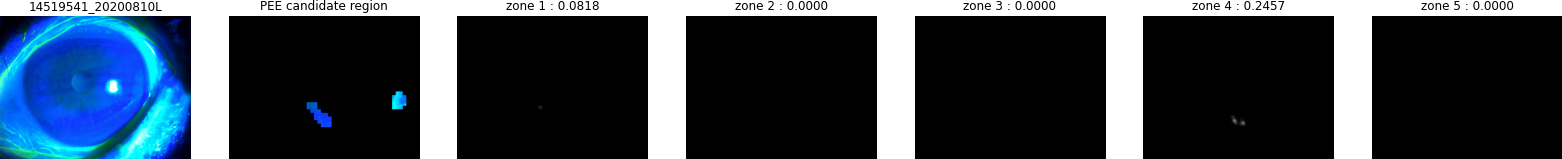

Supplement: S1 Dataset — (ZIP) [file pone.0299776.s002.zip › 14519541_20200810L/14519541_20200810L_zone.png]

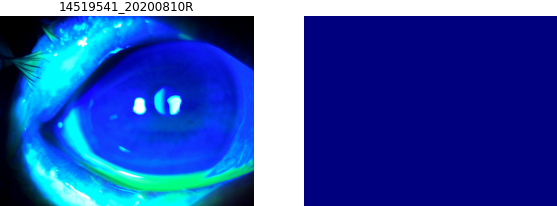

Supplement: S1 Dataset — (ZIP) [file pone.0299776.s002.zip › 14519541_20200810R/14519541_20200810R_densitymap.png]

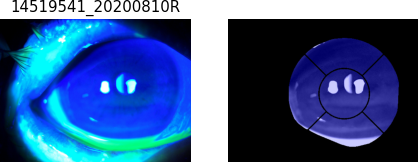

Supplement: S1 Dataset — (ZIP) [file pone.0299776.s002.zip › 14519541_20200810R/14519541_20200810R_whole.png]

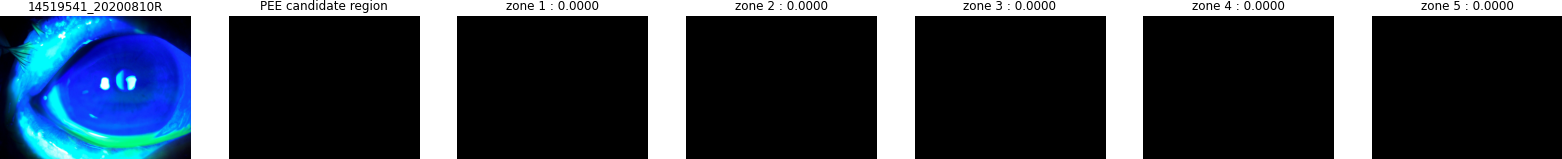

Supplement: S1 Dataset — (ZIP) [file pone.0299776.s002.zip › 14519541_20200810R/14519541_20200810R_zone.png]

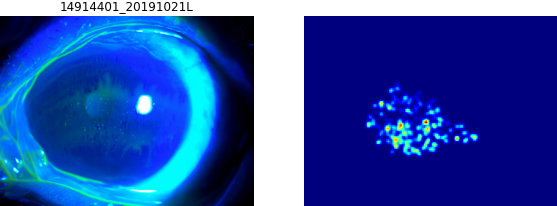

Supplement: S1 Dataset — (ZIP) [file pone.0299776.s002.zip › 14914401_20191021L/14914401_20191021L_densitymap.png]

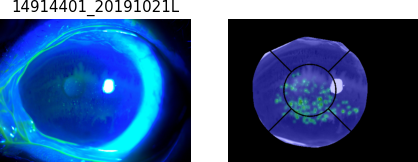

Supplement: S1 Dataset — (ZIP) [file pone.0299776.s002.zip › 14914401_20191021L/14914401_20191021L_whole.png]

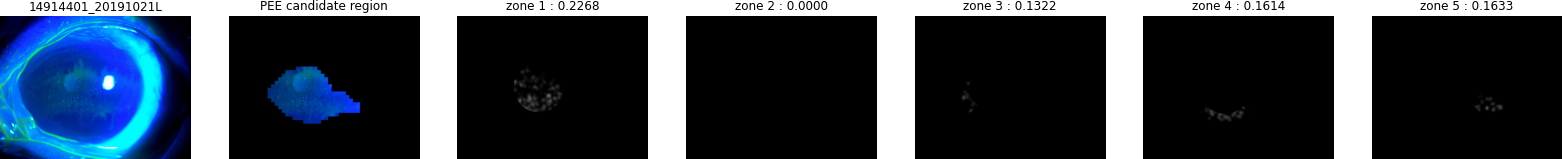

Supplement: S1 Dataset — (ZIP) [file pone.0299776.s002.zip › 14914401_20191021L/14914401_20191021L_zone.png]

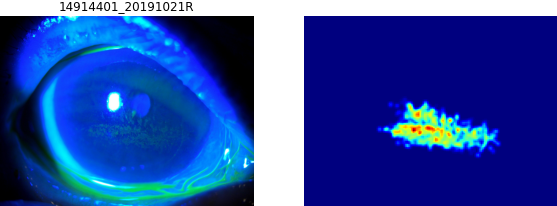

Supplement: S1 Dataset — (ZIP) [file pone.0299776.s002.zip › 14914401_20191021R/14914401_20191021R_densitymap.png]

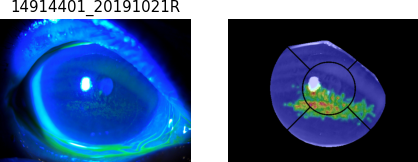

Supplement: S1 Dataset — (ZIP) [file pone.0299776.s002.zip › 14914401_20191021R/14914401_20191021R_whole.png]

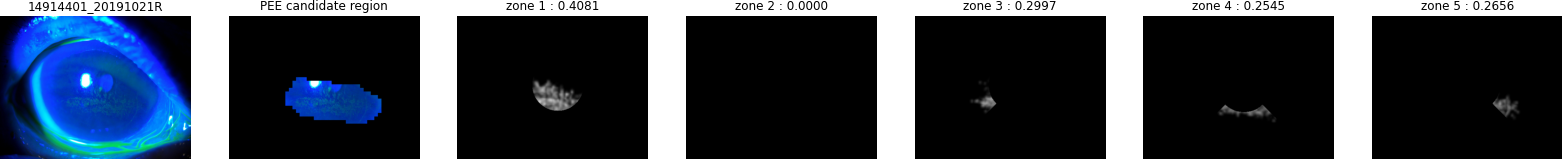

Supplement: S1 Dataset — (ZIP) [file pone.0299776.s002.zip › 14914401_20191021R/14914401_20191021R_zone.png]

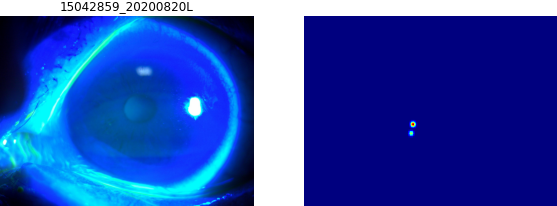

Supplement: S1 Dataset — (ZIP) [file pone.0299776.s002.zip › 15042859_20200820L/15042859_20200820L_densitymap.png]

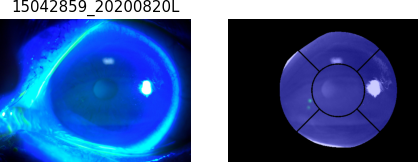

Supplement: S1 Dataset — (ZIP) [file pone.0299776.s002.zip › 15042859_20200820L/15042859_20200820L_whole.png]

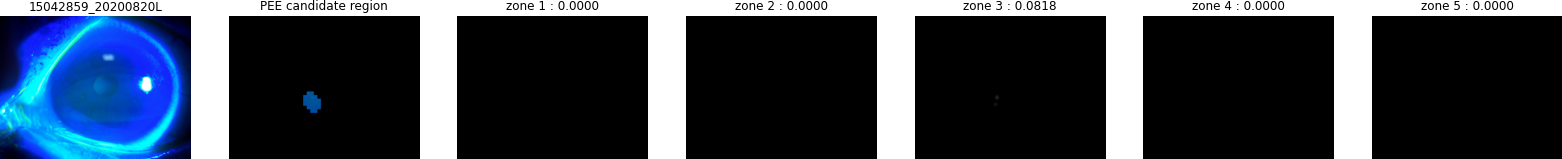

Supplement: S1 Dataset — (ZIP) [file pone.0299776.s002.zip › 15042859_20200820L/15042859_20200820L_zone.png]

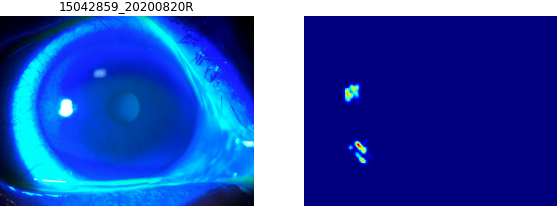

Supplement: S1 Dataset — (ZIP) [file pone.0299776.s002.zip › 15042859_20200820R/15042859_20200820R_densitymap.png]

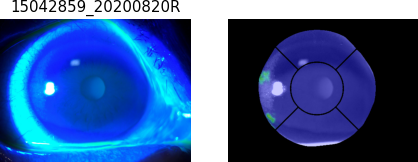

Supplement: S1 Dataset — (ZIP) [file pone.0299776.s002.zip › 15042859_20200820R/15042859_20200820R_whole.png]

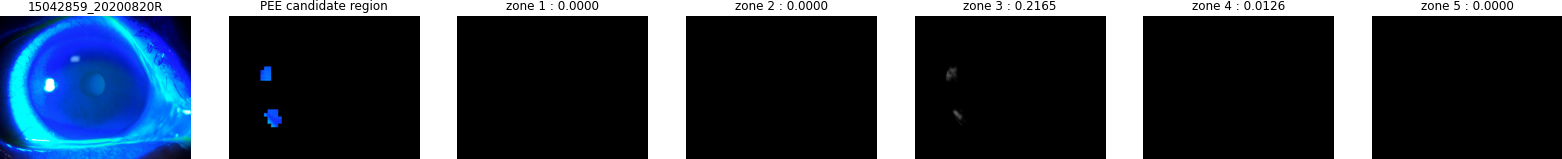

Supplement: S1 Dataset — (ZIP) [file pone.0299776.s002.zip › 15042859_20200820R/15042859_20200820R_zone.png]

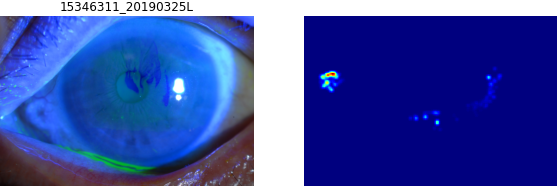

Supplement: S1 Dataset — (ZIP) [file pone.0299776.s002.zip › 15346311_20190325L/15346311_20190325L_densitymap.png]

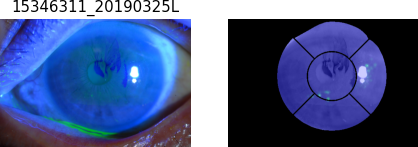

Supplement: S1 Dataset — (ZIP) [file pone.0299776.s002.zip › 15346311_20190325L/15346311_20190325L_whole.png]

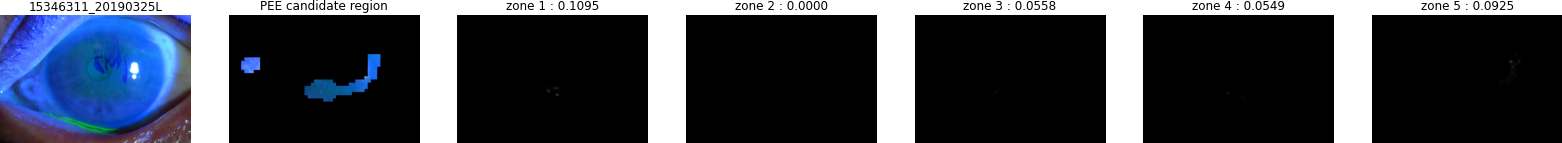

Supplement: S1 Dataset — (ZIP) [file pone.0299776.s002.zip › 15346311_20190325L/15346311_20190325L_zone.png]

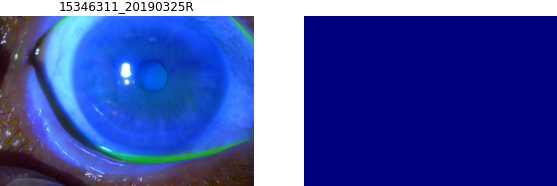

Supplement: S1 Dataset — (ZIP) [file pone.0299776.s002.zip › 15346311_20190325R/15346311_20190325R_densitymap.png]

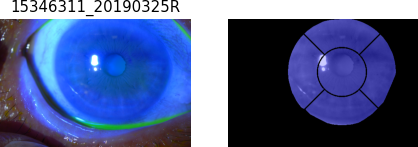

Supplement: S1 Dataset — (ZIP) [file pone.0299776.s002.zip › 15346311_20190325R/15346311_20190325R_whole.png]

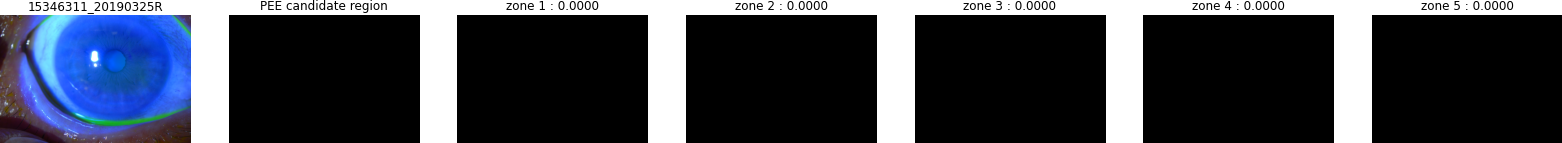

Supplement: S1 Dataset — (ZIP) [file pone.0299776.s002.zip › 15346311_20190325R/15346311_20190325R_zone.png]

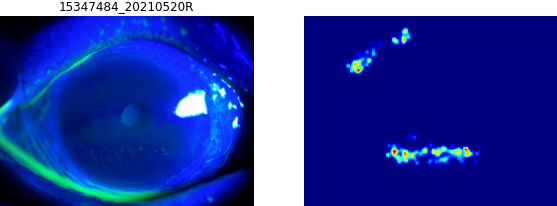

Supplement: S1 Dataset — (ZIP) [file pone.0299776.s002.zip › 15347484_20210520R/15347484_20210520R_densitymap.png]

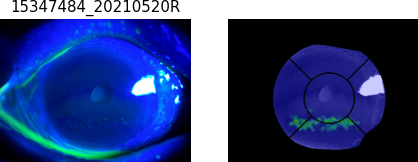

Supplement: S1 Dataset — (ZIP) [file pone.0299776.s002.zip › 15347484_20210520R/15347484_20210520R_whole.png]

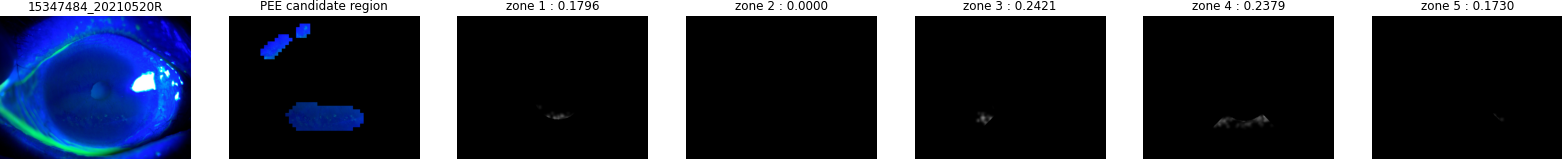

Supplement: S1 Dataset — (ZIP) [file pone.0299776.s002.zip › 15347484_20210520R/15347484_20210520R_zone.png]

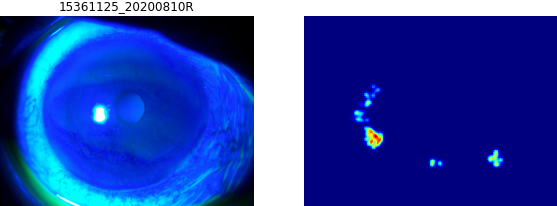

Supplement: S1 Dataset — (ZIP) [file pone.0299776.s002.zip › 15361125_20200810R/15361125_20200810R_densitymap.png]

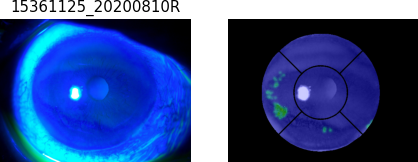

Supplement: S1 Dataset — (ZIP) [file pone.0299776.s002.zip › 15361125_20200810R/15361125_20200810R_whole.png]

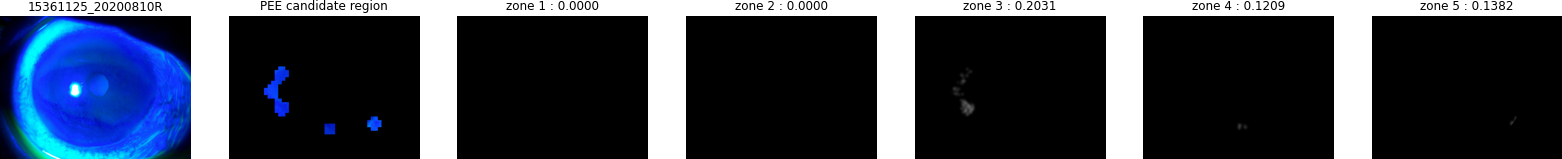

Supplement: S1 Dataset — (ZIP) [file pone.0299776.s002.zip › 15361125_20200810R/15361125_20200810R_zone.png]

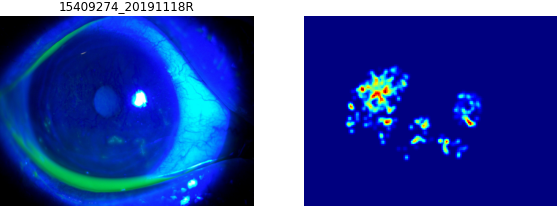

Supplement: S1 Dataset — (ZIP) [file pone.0299776.s002.zip › 15409274_20191118R/15409274_20191118R_densitymap.png]

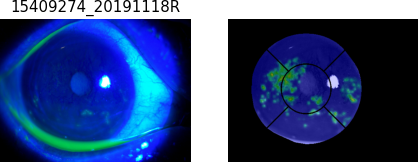

Supplement: S1 Dataset — (ZIP) [file pone.0299776.s002.zip › 15409274_20191118R/15409274_20191118R_whole.png]

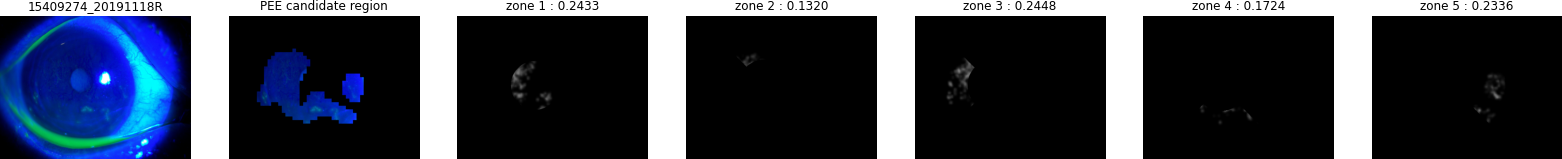

Supplement: S1 Dataset — (ZIP) [file pone.0299776.s002.zip › 15409274_20191118R/15409274_20191118R_zone.png]

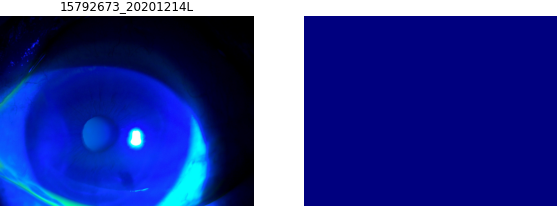

Supplement: S1 Dataset — (ZIP) [file pone.0299776.s002.zip › 15792673_20201214L/15792673_20201214L_densitymap.png]

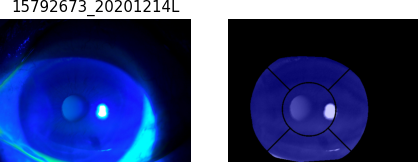

Supplement: S1 Dataset — (ZIP) [file pone.0299776.s002.zip › 15792673_20201214L/15792673_20201214L_whole.png]

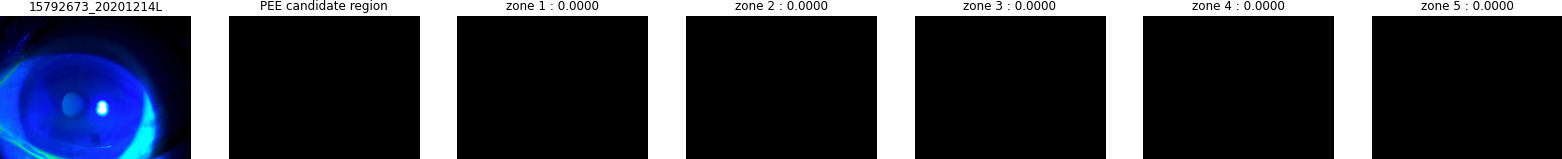

Supplement: S1 Dataset — (ZIP) [file pone.0299776.s002.zip › 15792673_20201214L/15792673_20201214L_zone.png]

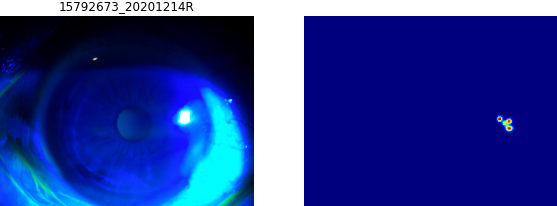

Supplement: S1 Dataset — (ZIP) [file pone.0299776.s002.zip › 15792673_20201214R/15792673_20201214R_densitymap.png]

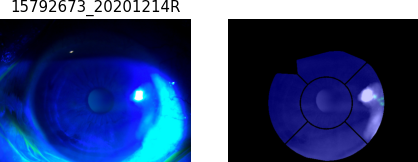

Supplement: S1 Dataset — (ZIP) [file pone.0299776.s002.zip › 15792673_20201214R/15792673_20201214R_whole.png]

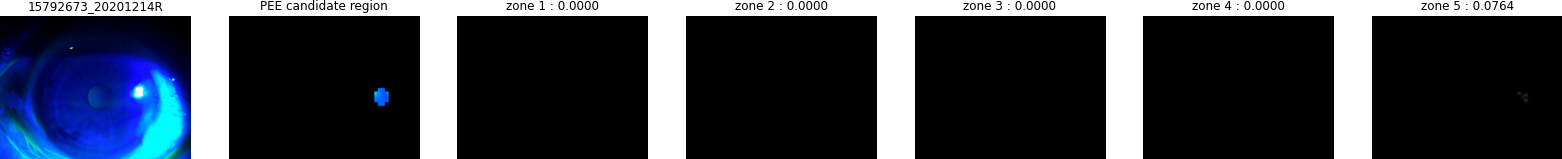

Supplement: S1 Dataset — (ZIP) [file pone.0299776.s002.zip › 15792673_20201214R/15792673_20201214R_zone.png]

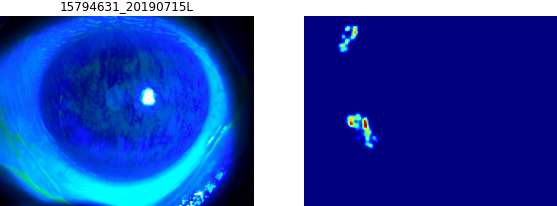

Supplement: S1 Dataset — (ZIP) [file pone.0299776.s002.zip › 15794631_20190715L/15794631_20190715L_densitymap.png]

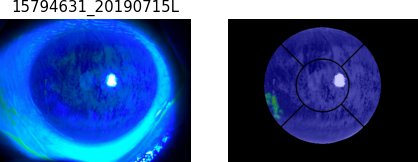

Supplement: S1 Dataset — (ZIP) [file pone.0299776.s002.zip › 15794631_20190715L/15794631_20190715L_whole.png]

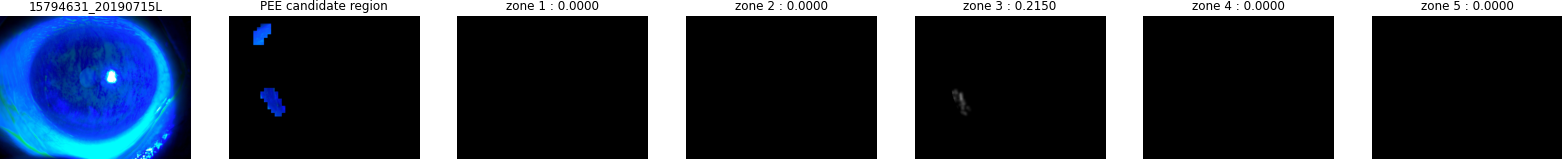

Supplement: S1 Dataset — (ZIP) [file pone.0299776.s002.zip › 15794631_20190715L/15794631_20190715L_zone.png]

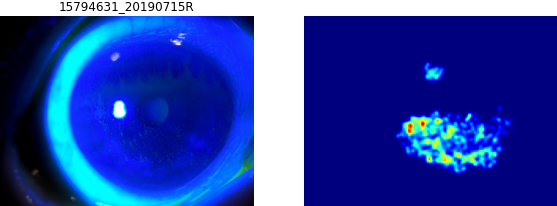

Supplement: S1 Dataset — (ZIP) [file pone.0299776.s002.zip › 15794631_20190715R/15794631_20190715R_densitymap.png]

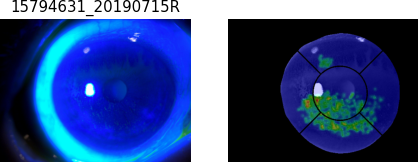

Supplement: S1 Dataset — (ZIP) [file pone.0299776.s002.zip › 15794631_20190715R/15794631_20190715R_whole.png]
