# Supplementary figures and images for: Deep learning-based fully automated grading system for dry eye disease severity (part 1 of 6)
Source: PLoS One. 2024 Mar 14;19(3):e0299776. doi: 10.1371/journal.pone.0299776 (PMC10939279; doi:10.1371/journal.pone.0299776)

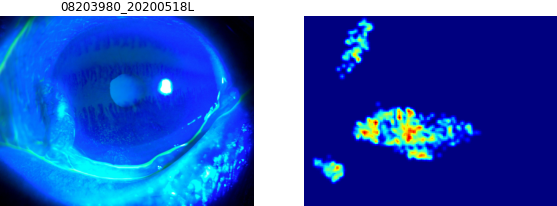

Supplement: S1 Dataset — (ZIP) [file pone.0299776.s002.zip › 08203980_20200518L/08203980_20200518L_densitymap.png]

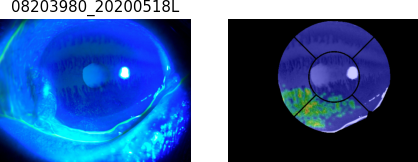

Supplement: S1 Dataset — (ZIP) [file pone.0299776.s002.zip › 08203980_20200518L/08203980_20200518L_whole.png]

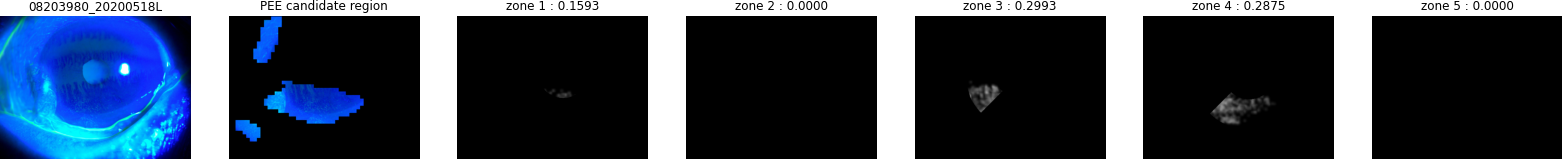

Supplement: S1 Dataset — (ZIP) [file pone.0299776.s002.zip › 08203980_20200518L/08203980_20200518L_zone.png]

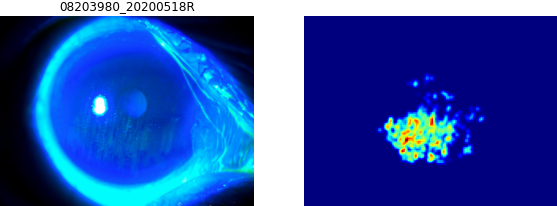

Supplement: S1 Dataset — (ZIP) [file pone.0299776.s002.zip › 08203980_20200518R/08203980_20200518R_densitymap.png]

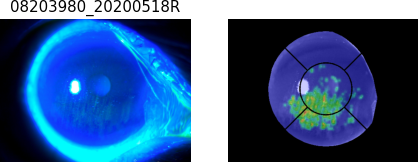

Supplement: S1 Dataset — (ZIP) [file pone.0299776.s002.zip › 08203980_20200518R/08203980_20200518R_whole.png]

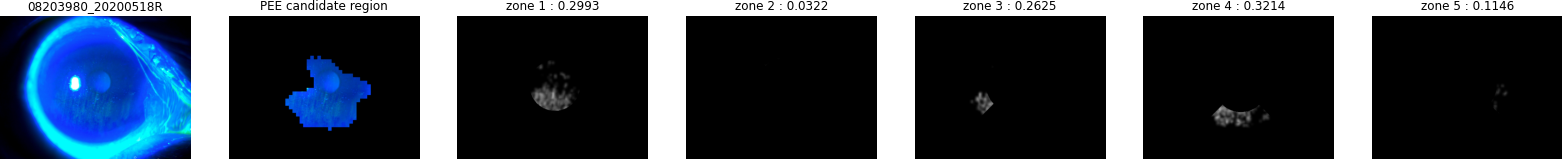

Supplement: S1 Dataset — (ZIP) [file pone.0299776.s002.zip › 08203980_20200518R/08203980_20200518R_zone.png]

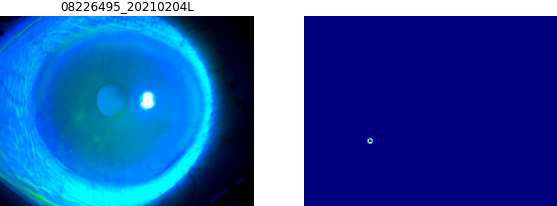

Supplement: S1 Dataset — (ZIP) [file pone.0299776.s002.zip › 08226495_20210204L/08226495_20210204L_densitymap.png]

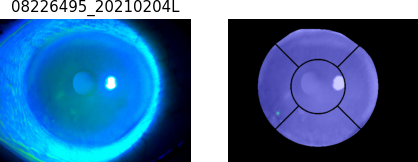

Supplement: S1 Dataset — (ZIP) [file pone.0299776.s002.zip › 08226495_20210204L/08226495_20210204L_whole.png]

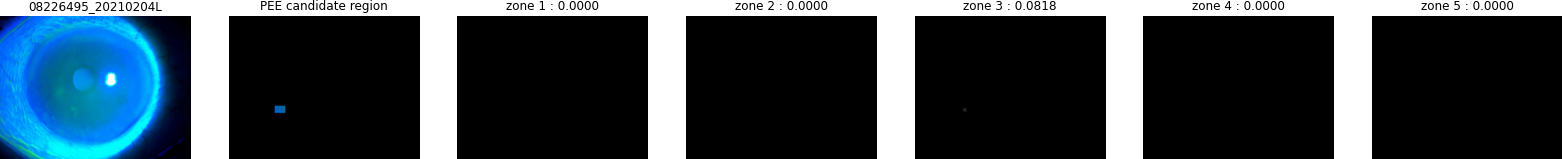

Supplement: S1 Dataset — (ZIP) [file pone.0299776.s002.zip › 08226495_20210204L/08226495_20210204L_zone.png]

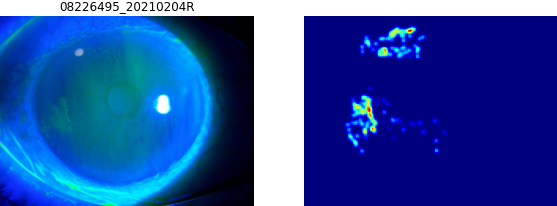

Supplement: S1 Dataset — (ZIP) [file pone.0299776.s002.zip › 08226495_20210204R/08226495_20210204R_densitymap.png]

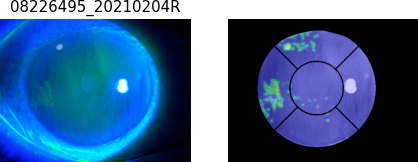

Supplement: S1 Dataset — (ZIP) [file pone.0299776.s002.zip › 08226495_20210204R/08226495_20210204R_whole.png]

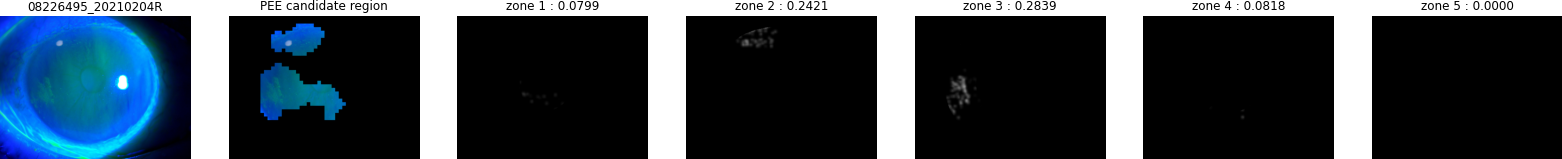

Supplement: S1 Dataset — (ZIP) [file pone.0299776.s002.zip › 08226495_20210204R/08226495_20210204R_zone.png]

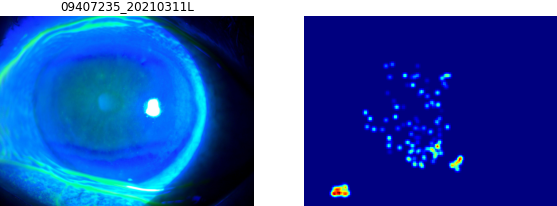

Supplement: S1 Dataset — (ZIP) [file pone.0299776.s002.zip › 09407235_20210311L/09407235_20210311L_densitymap.png]

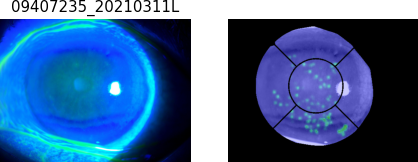

Supplement: S1 Dataset — (ZIP) [file pone.0299776.s002.zip › 09407235_20210311L/09407235_20210311L_whole.png]

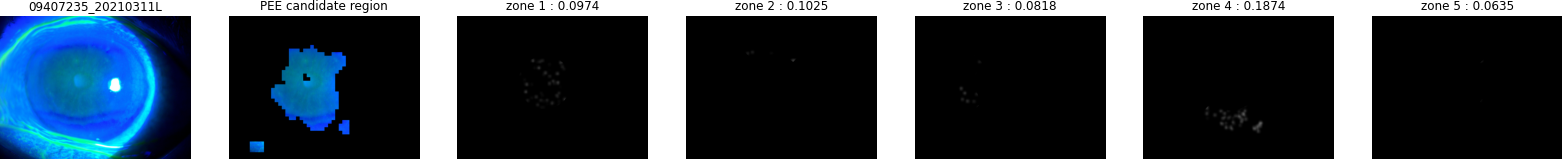

Supplement: S1 Dataset — (ZIP) [file pone.0299776.s002.zip › 09407235_20210311L/09407235_20210311L_zone.png]

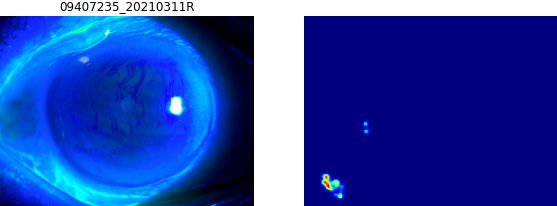

Supplement: S1 Dataset — (ZIP) [file pone.0299776.s002.zip › 09407235_20210311R/09407235_20210311R_densitymap.png]

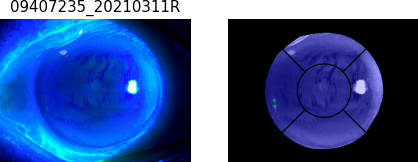

Supplement: S1 Dataset — (ZIP) [file pone.0299776.s002.zip › 09407235_20210311R/09407235_20210311R_whole.png]

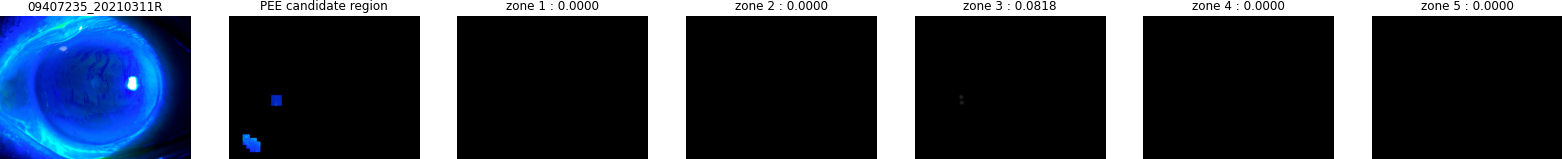

Supplement: S1 Dataset — (ZIP) [file pone.0299776.s002.zip › 09407235_20210311R/09407235_20210311R_zone.png]

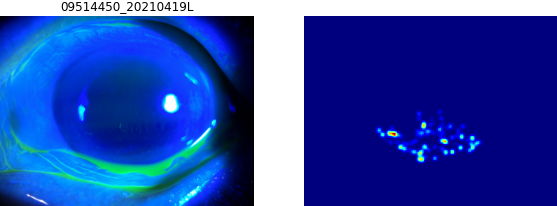

Supplement: S1 Dataset — (ZIP) [file pone.0299776.s002.zip › 09514450_20210419L/09514450_20210419L_densitymap.png]

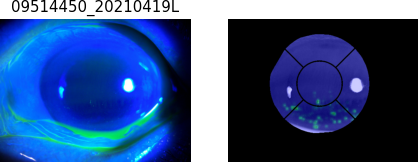

Supplement: S1 Dataset — (ZIP) [file pone.0299776.s002.zip › 09514450_20210419L/09514450_20210419L_whole.png]

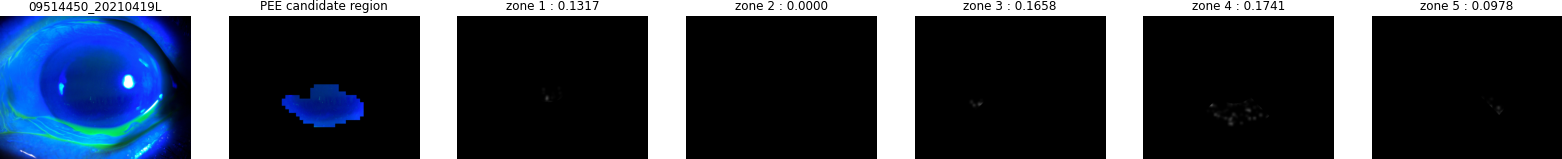

Supplement: S1 Dataset — (ZIP) [file pone.0299776.s002.zip › 09514450_20210419L/09514450_20210419L_zone.png]

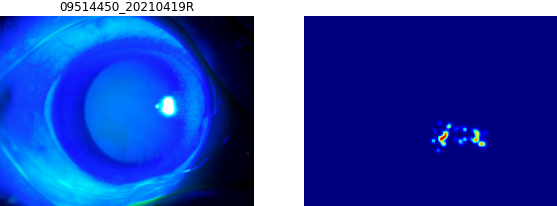

Supplement: S1 Dataset — (ZIP) [file pone.0299776.s002.zip › 09514450_20210419R/09514450_20210419R_densitymap.png]

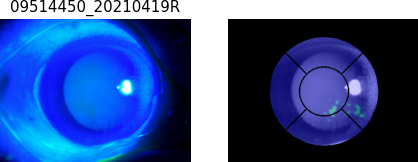

Supplement: S1 Dataset — (ZIP) [file pone.0299776.s002.zip › 09514450_20210419R/09514450_20210419R_whole.png]

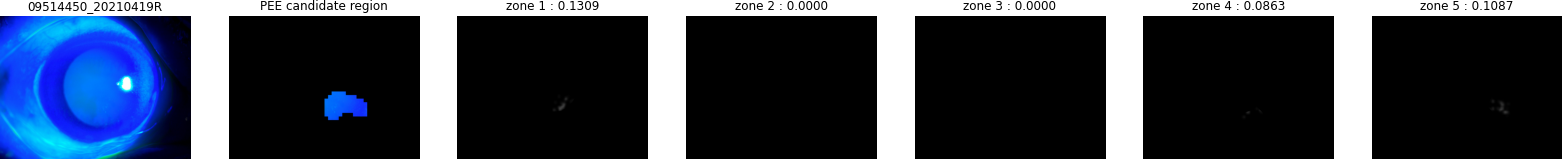

Supplement: S1 Dataset — (ZIP) [file pone.0299776.s002.zip › 09514450_20210419R/09514450_20210419R_zone.png]

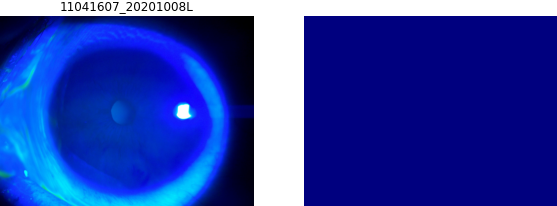

Supplement: S1 Dataset — (ZIP) [file pone.0299776.s002.zip › 11041607_20201008L/11041607_20201008L_densitymap.png]

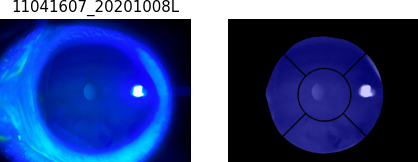

Supplement: S1 Dataset — (ZIP) [file pone.0299776.s002.zip › 11041607_20201008L/11041607_20201008L_whole.png]

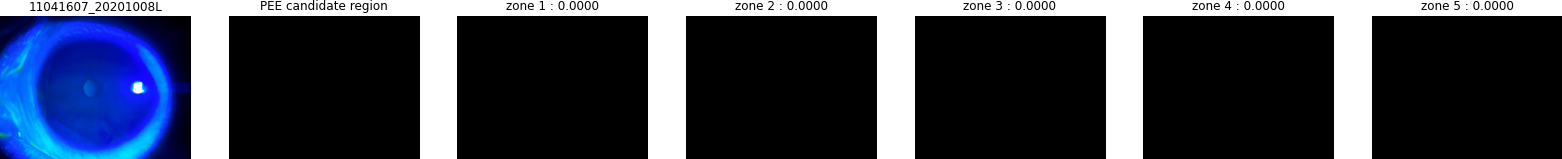

Supplement: S1 Dataset — (ZIP) [file pone.0299776.s002.zip › 11041607_20201008L/11041607_20201008L_zone.png]

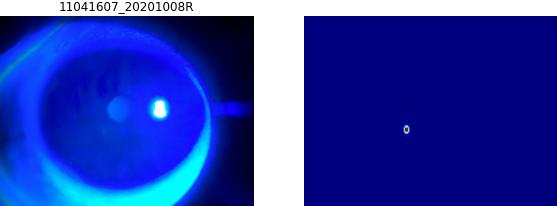

Supplement: S1 Dataset — (ZIP) [file pone.0299776.s002.zip › 11041607_20201008R/11041607_20201008R_densitymap.png]

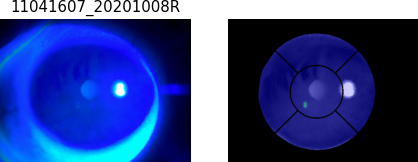

Supplement: S1 Dataset — (ZIP) [file pone.0299776.s002.zip › 11041607_20201008R/11041607_20201008R_whole.png]

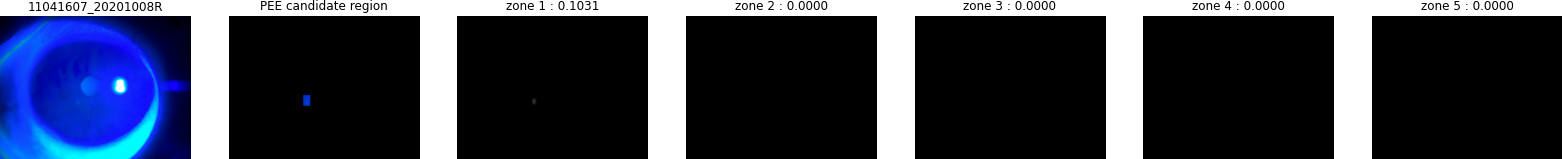

Supplement: S1 Dataset — (ZIP) [file pone.0299776.s002.zip › 11041607_20201008R/11041607_20201008R_zone.png]

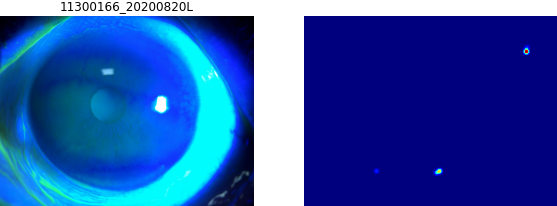

Supplement: S1 Dataset — (ZIP) [file pone.0299776.s002.zip › 11300166_20200820L/11300166_20200820L_densitymap.png]

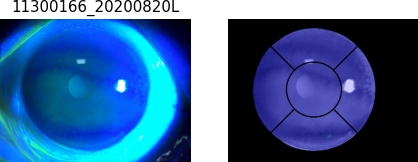

Supplement: S1 Dataset — (ZIP) [file pone.0299776.s002.zip › 11300166_20200820L/11300166_20200820L_whole.png]

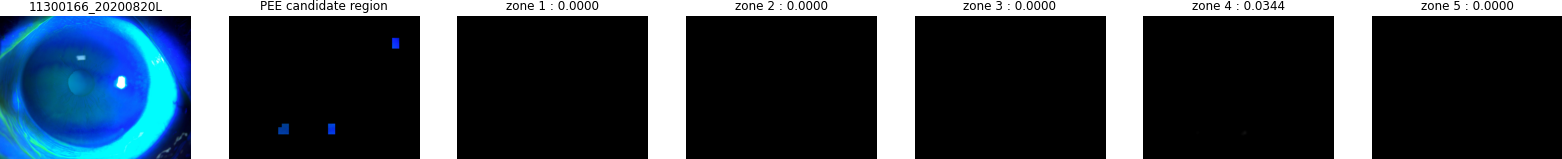

Supplement: S1 Dataset — (ZIP) [file pone.0299776.s002.zip › 11300166_20200820L/11300166_20200820L_zone.png]

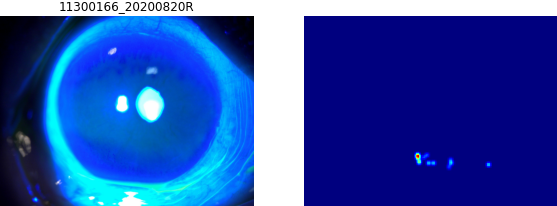

Supplement: S1 Dataset — (ZIP) [file pone.0299776.s002.zip › 11300166_20200820R/11300166_20200820R_densitymap.png]

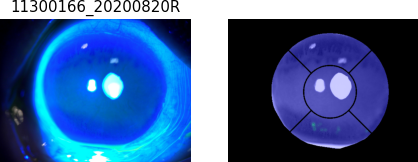

Supplement: S1 Dataset — (ZIP) [file pone.0299776.s002.zip › 11300166_20200820R/11300166_20200820R_whole.png]

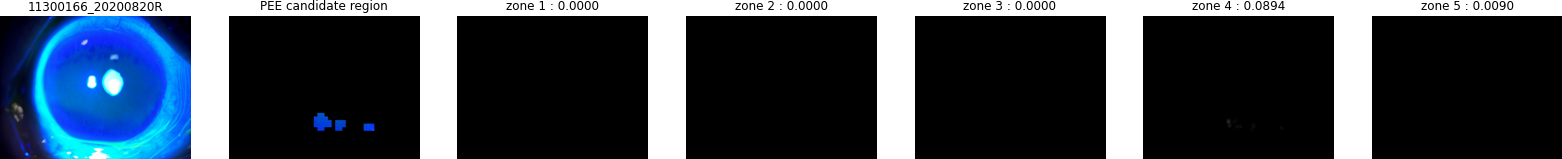

Supplement: S1 Dataset — (ZIP) [file pone.0299776.s002.zip › 11300166_20200820R/11300166_20200820R_zone.png]

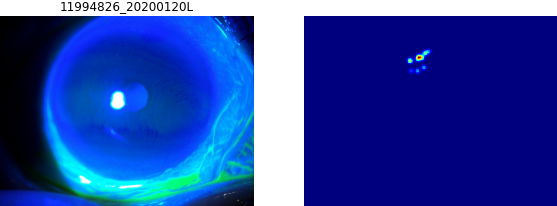

Supplement: S1 Dataset — (ZIP) [file pone.0299776.s002.zip › 11994826_20200120L/11994826_20200120L_densitymap.png]

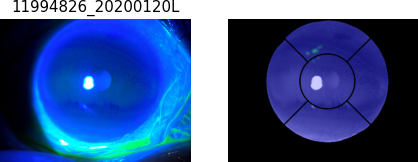

Supplement: S1 Dataset — (ZIP) [file pone.0299776.s002.zip › 11994826_20200120L/11994826_20200120L_whole.png]

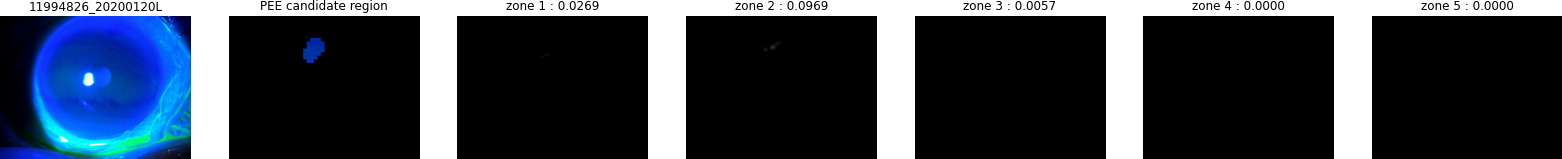

Supplement: S1 Dataset — (ZIP) [file pone.0299776.s002.zip › 11994826_20200120L/11994826_20200120L_zone.png]

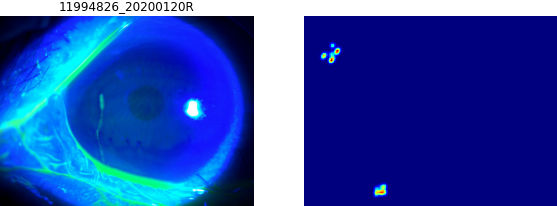

Supplement: S1 Dataset — (ZIP) [file pone.0299776.s002.zip › 11994826_20200120R/11994826_20200120R_densitymap.png]

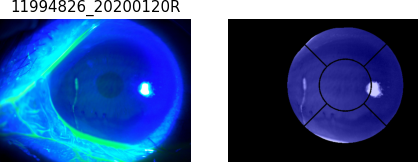

Supplement: S1 Dataset — (ZIP) [file pone.0299776.s002.zip › 11994826_20200120R/11994826_20200120R_whole.png]

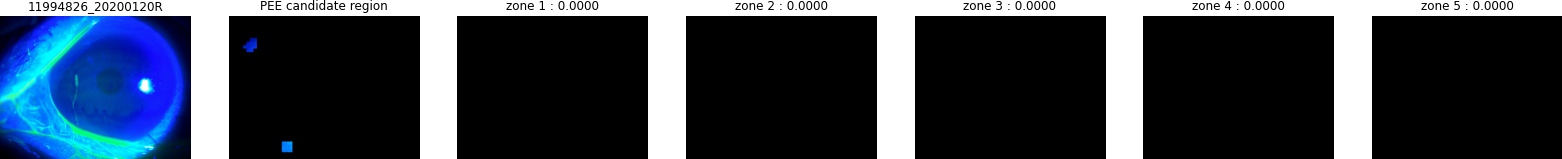

Supplement: S1 Dataset — (ZIP) [file pone.0299776.s002.zip › 11994826_20200120R/11994826_20200120R_zone.png]

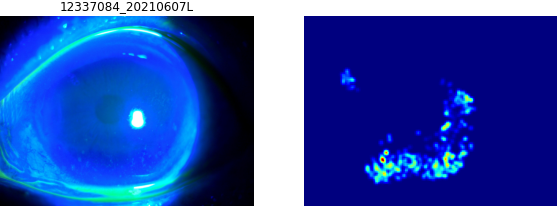

Supplement: S1 Dataset — (ZIP) [file pone.0299776.s002.zip › 12337084_20210607L/12337084_20210607L_densitymap.png]

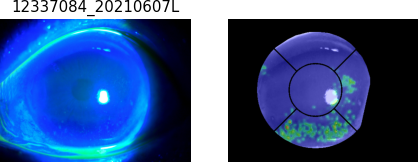

Supplement: S1 Dataset — (ZIP) [file pone.0299776.s002.zip › 12337084_20210607L/12337084_20210607L_whole.png]

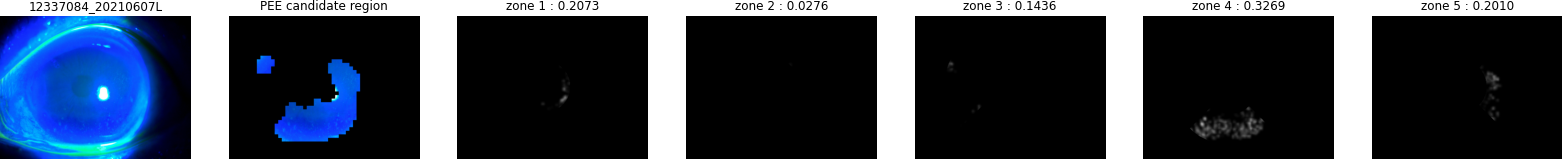

Supplement: S1 Dataset — (ZIP) [file pone.0299776.s002.zip › 12337084_20210607L/12337084_20210607L_zone.png]

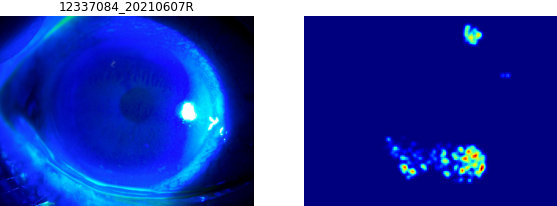

Supplement: S1 Dataset — (ZIP) [file pone.0299776.s002.zip › 12337084_20210607R/12337084_20210607R_densitymap.png]

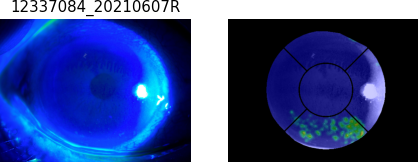

Supplement: S1 Dataset — (ZIP) [file pone.0299776.s002.zip › 12337084_20210607R/12337084_20210607R_whole.png]

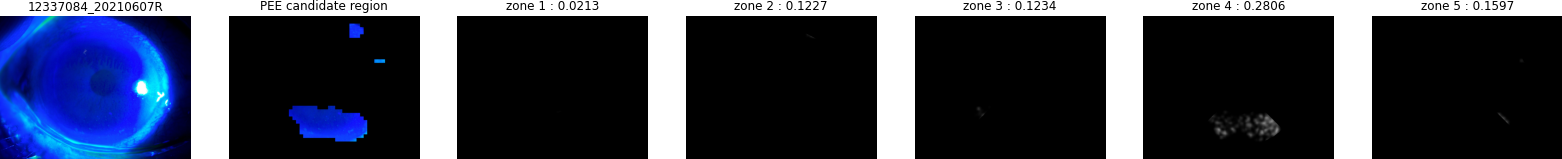

Supplement: S1 Dataset — (ZIP) [file pone.0299776.s002.zip › 12337084_20210607R/12337084_20210607R_zone.png]

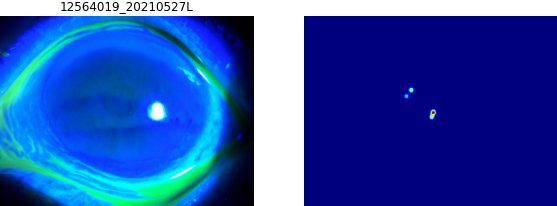

Supplement: S1 Dataset — (ZIP) [file pone.0299776.s002.zip › 12564019_20210527L/12564019_20210527L_densitymap.png]

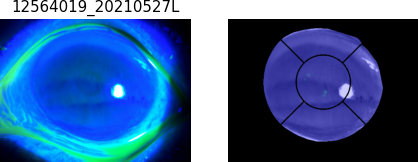

Supplement: S1 Dataset — (ZIP) [file pone.0299776.s002.zip › 12564019_20210527L/12564019_20210527L_whole.png]

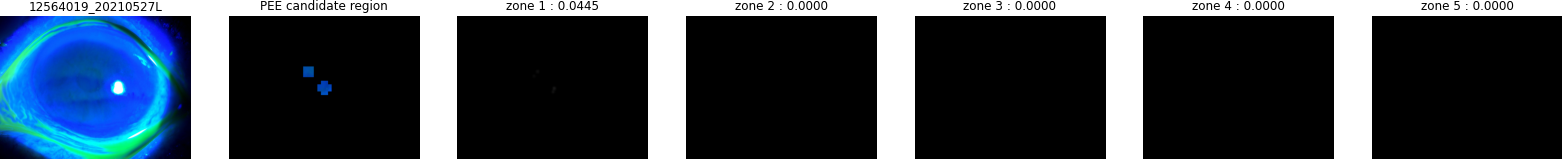

Supplement: S1 Dataset — (ZIP) [file pone.0299776.s002.zip › 12564019_20210527L/12564019_20210527L_zone.png]

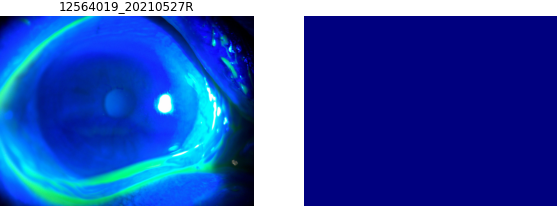

Supplement: S1 Dataset — (ZIP) [file pone.0299776.s002.zip › 12564019_20210527R/12564019_20210527R_densitymap.png]

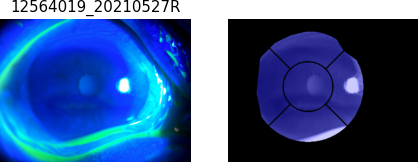

Supplement: S1 Dataset — (ZIP) [file pone.0299776.s002.zip › 12564019_20210527R/12564019_20210527R_whole.png]

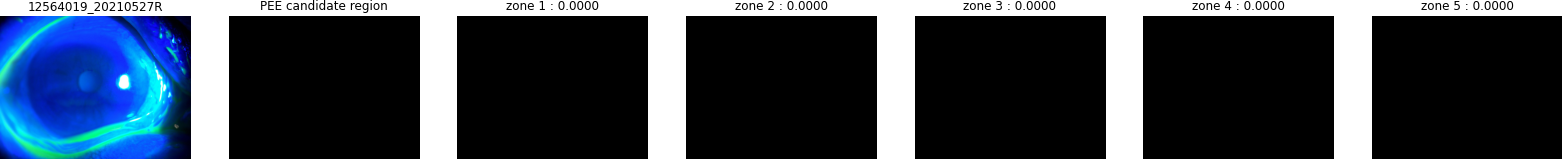

Supplement: S1 Dataset — (ZIP) [file pone.0299776.s002.zip › 12564019_20210527R/12564019_20210527R_zone.png]

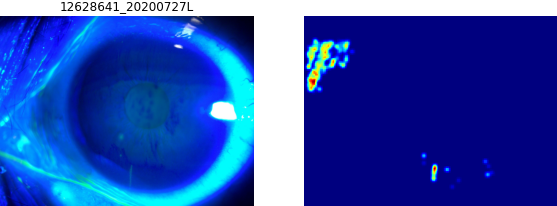

Supplement: S1 Dataset — (ZIP) [file pone.0299776.s002.zip › 12628641_20200727L/12628641_20200727L_densitymap.png]

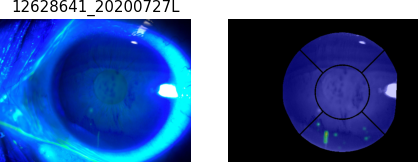

Supplement: S1 Dataset — (ZIP) [file pone.0299776.s002.zip › 12628641_20200727L/12628641_20200727L_whole.png]

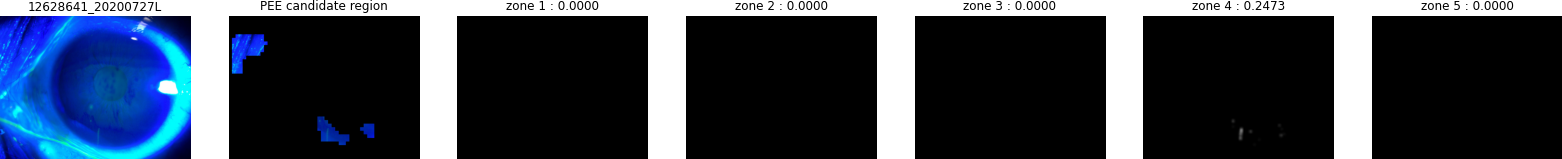

Supplement: S1 Dataset — (ZIP) [file pone.0299776.s002.zip › 12628641_20200727L/12628641_20200727L_zone.png]

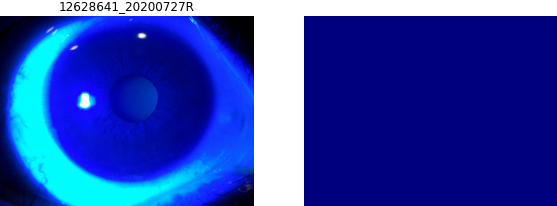

Supplement: S1 Dataset — (ZIP) [file pone.0299776.s002.zip › 12628641_20200727R/12628641_20200727R_densitymap.png]

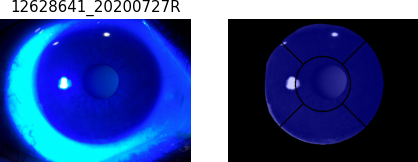

Supplement: S1 Dataset — (ZIP) [file pone.0299776.s002.zip › 12628641_20200727R/12628641_20200727R_whole.png]

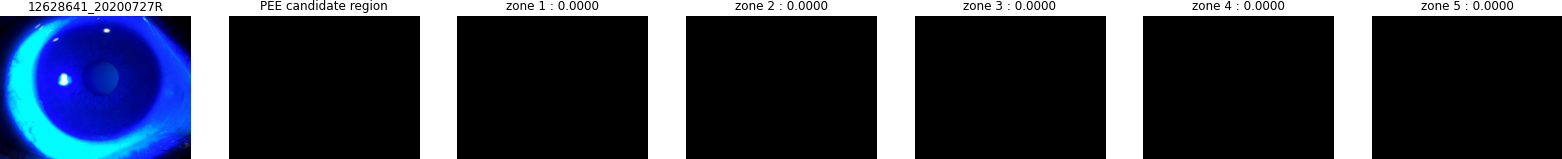

Supplement: S1 Dataset — (ZIP) [file pone.0299776.s002.zip › 12628641_20200727R/12628641_20200727R_zone.png]

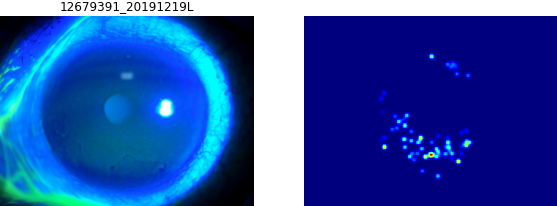

Supplement: S1 Dataset — (ZIP) [file pone.0299776.s002.zip › 12679391_20191219L/12679391_20191219L_densitymap.png]

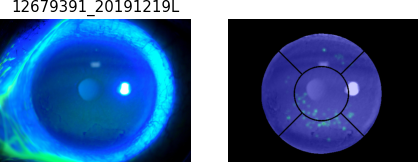

Supplement: S1 Dataset — (ZIP) [file pone.0299776.s002.zip › 12679391_20191219L/12679391_20191219L_whole.png]

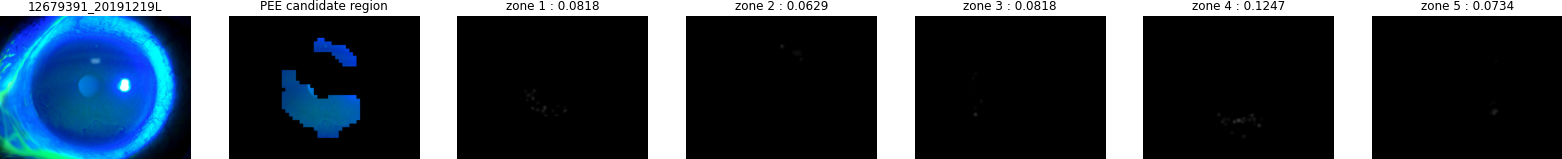

Supplement: S1 Dataset — (ZIP) [file pone.0299776.s002.zip › 12679391_20191219L/12679391_20191219L_zone.png]

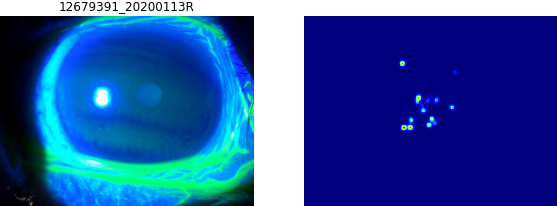

Supplement: S1 Dataset — (ZIP) [file pone.0299776.s002.zip › 12679391_20200113R/12679391_20200113R_densitymap.png]

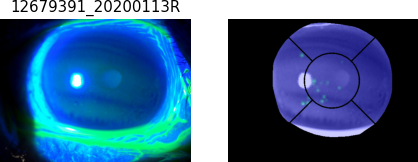

Supplement: S1 Dataset — (ZIP) [file pone.0299776.s002.zip › 12679391_20200113R/12679391_20200113R_whole.png]

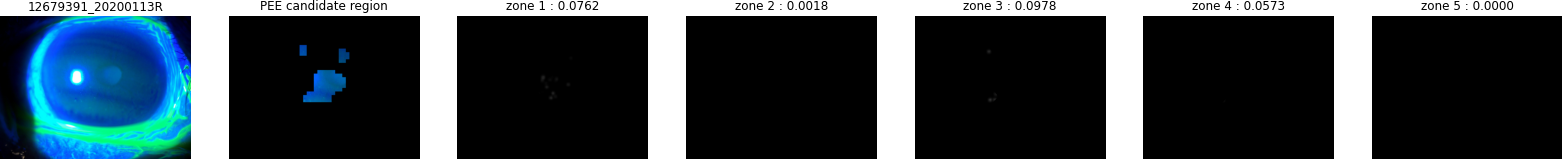

Supplement: S1 Dataset — (ZIP) [file pone.0299776.s002.zip › 12679391_20200113R/12679391_20200113R_zone.png]

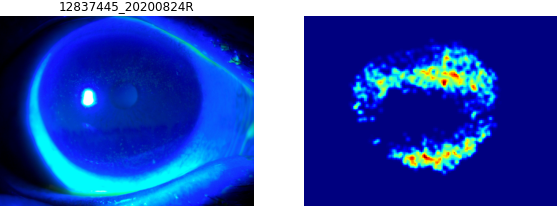

Supplement: S1 Dataset — (ZIP) [file pone.0299776.s002.zip › 12837445_20200824R/12837445_20200824R_densitymap.png]

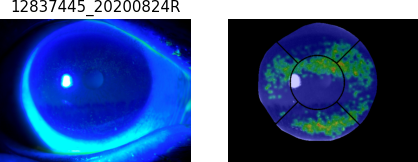

Supplement: S1 Dataset — (ZIP) [file pone.0299776.s002.zip › 12837445_20200824R/12837445_20200824R_whole.png]

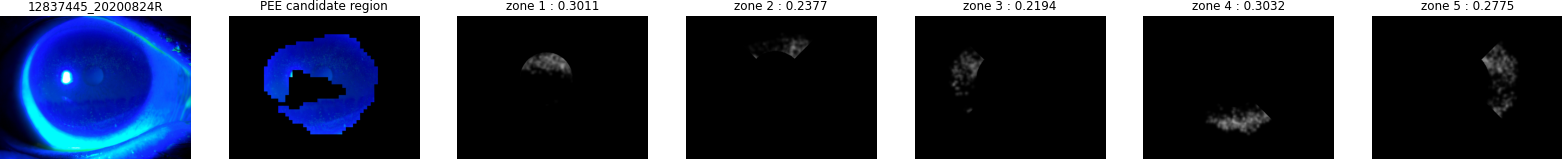

Supplement: S1 Dataset — (ZIP) [file pone.0299776.s002.zip › 12837445_20200824R/12837445_20200824R_zone.png]

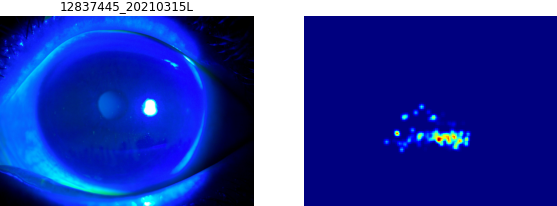

Supplement: S1 Dataset — (ZIP) [file pone.0299776.s002.zip › 12837445_20210315L/12837445_20210315L_densitymap.png]

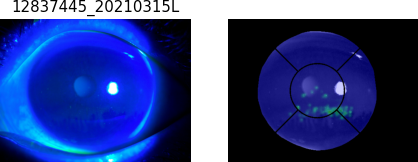

Supplement: S1 Dataset — (ZIP) [file pone.0299776.s002.zip › 12837445_20210315L/12837445_20210315L_whole.png]

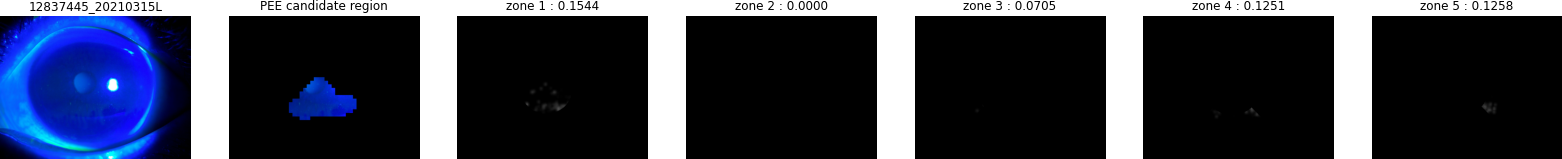

Supplement: S1 Dataset — (ZIP) [file pone.0299776.s002.zip › 12837445_20210315L/12837445_20210315L_zone.png]

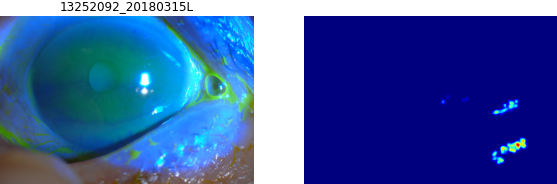

Supplement: S1 Dataset — (ZIP) [file pone.0299776.s002.zip › 13252092_20180315L/13252092_20180315L_densitymap.png]

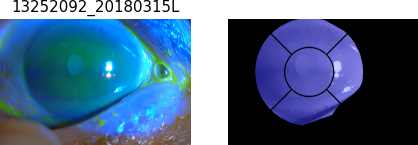

Supplement: S1 Dataset — (ZIP) [file pone.0299776.s002.zip › 13252092_20180315L/13252092_20180315L_whole.png]

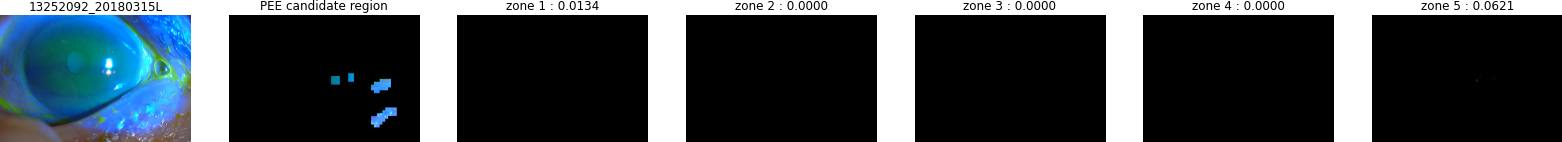

Supplement: S1 Dataset — (ZIP) [file pone.0299776.s002.zip › 13252092_20180315L/13252092_20180315L_zone.png]

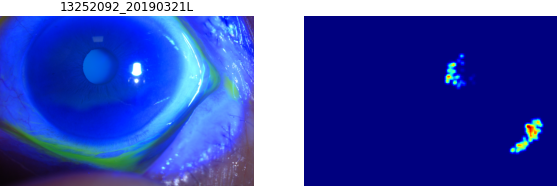

Supplement: S1 Dataset — (ZIP) [file pone.0299776.s002.zip › 13252092_20190321L/13252092_20190321L_densitymap.png]

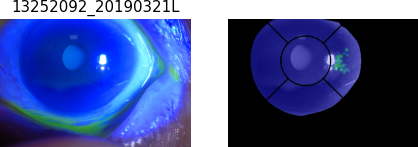

Supplement: S1 Dataset — (ZIP) [file pone.0299776.s002.zip › 13252092_20190321L/13252092_20190321L_whole.png]

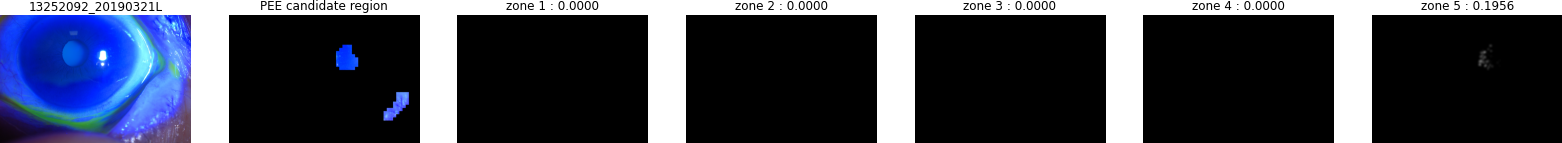

Supplement: S1 Dataset — (ZIP) [file pone.0299776.s002.zip › 13252092_20190321L/13252092_20190321L_zone.png]

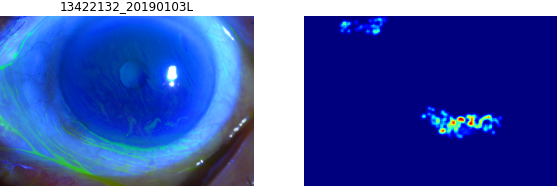

Supplement: S1 Dataset — (ZIP) [file pone.0299776.s002.zip › 13422132_20190103L/13422132_20190103L_densitymap.png]

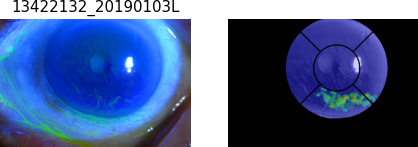

Supplement: S1 Dataset — (ZIP) [file pone.0299776.s002.zip › 13422132_20190103L/13422132_20190103L_whole.png]

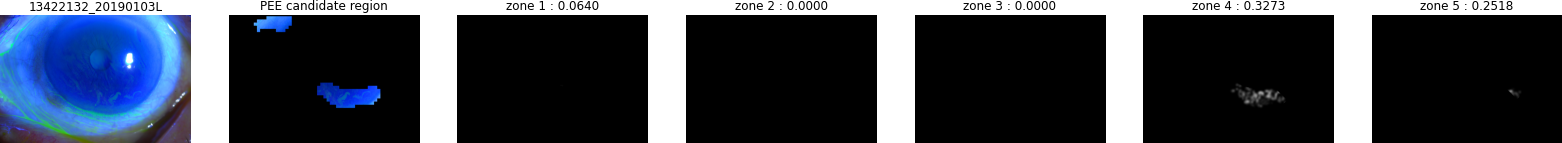

Supplement: S1 Dataset — (ZIP) [file pone.0299776.s002.zip › 13422132_20190103L/13422132_20190103L_zone.png]

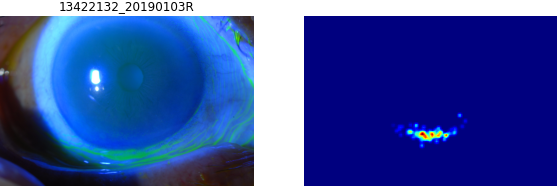

Supplement: S1 Dataset — (ZIP) [file pone.0299776.s002.zip › 13422132_20190103R/13422132_20190103R_densitymap.png]

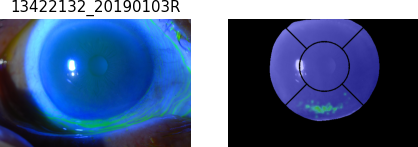

Supplement: S1 Dataset — (ZIP) [file pone.0299776.s002.zip › 13422132_20190103R/13422132_20190103R_whole.png]

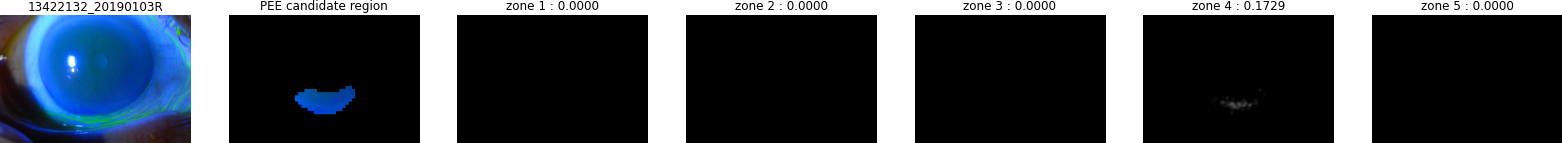

Supplement: S1 Dataset — (ZIP) [file pone.0299776.s002.zip › 13422132_20190103R/13422132_20190103R_zone.png]

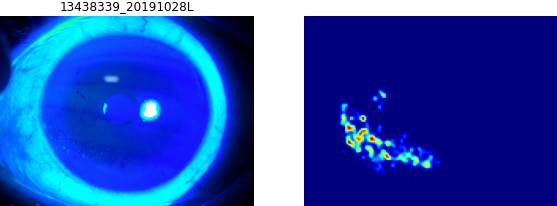

Supplement: S1 Dataset — (ZIP) [file pone.0299776.s002.zip › 13438339_20191028L/13438339_20191028L_densitymap.png]

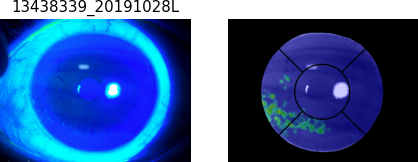

Supplement: S1 Dataset — (ZIP) [file pone.0299776.s002.zip › 13438339_20191028L/13438339_20191028L_whole.png]

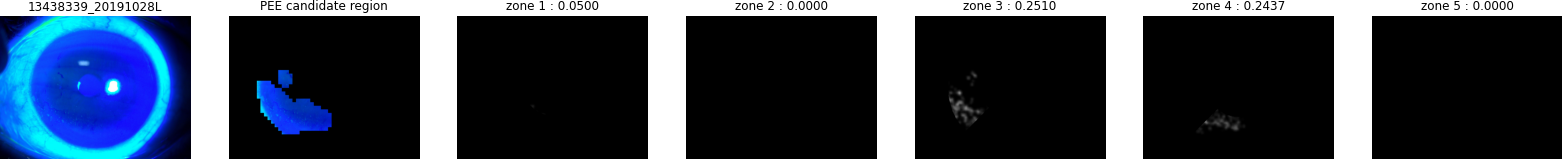

Supplement: S1 Dataset — (ZIP) [file pone.0299776.s002.zip › 13438339_20191028L/13438339_20191028L_zone.png]

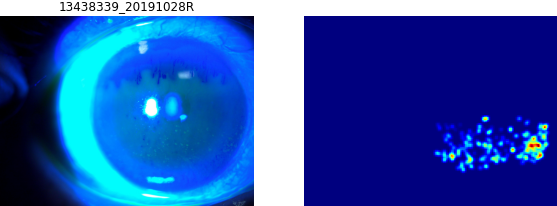

Supplement: S1 Dataset — (ZIP) [file pone.0299776.s002.zip › 13438339_20191028R/13438339_20191028R_densitymap.png]

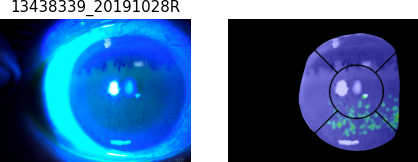

Supplement: S1 Dataset — (ZIP) [file pone.0299776.s002.zip › 13438339_20191028R/13438339_20191028R_whole.png]

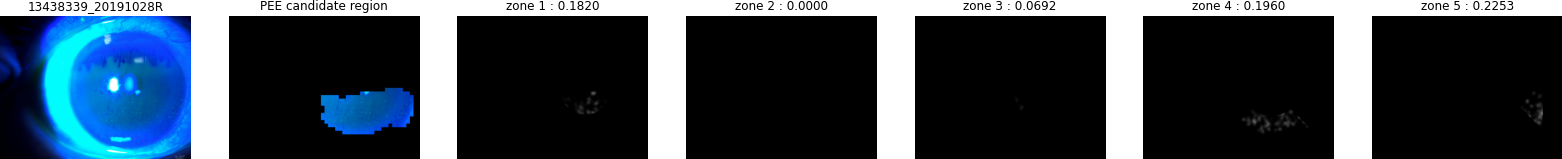

Supplement: S1 Dataset — (ZIP) [file pone.0299776.s002.zip › 13438339_20191028R/13438339_20191028R_zone.png]

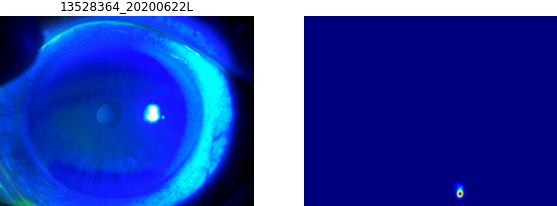

Supplement: S1 Dataset — (ZIP) [file pone.0299776.s002.zip › 13528364_20200622L/13528364_20200622L_densitymap.png]

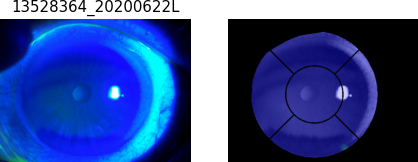

Supplement: S1 Dataset — (ZIP) [file pone.0299776.s002.zip › 13528364_20200622L/13528364_20200622L_whole.png]

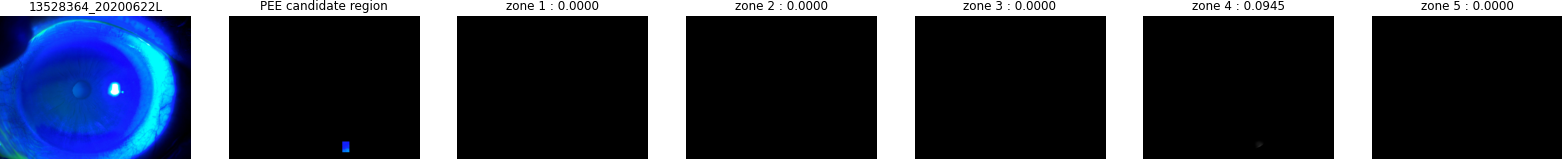

Supplement: S1 Dataset — (ZIP) [file pone.0299776.s002.zip › 13528364_20200622L/13528364_20200622L_zone.png]

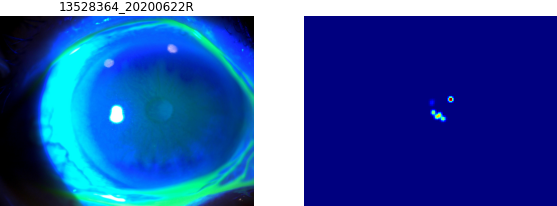

Supplement: S1 Dataset — (ZIP) [file pone.0299776.s002.zip › 13528364_20200622R/13528364_20200622R_densitymap.png]

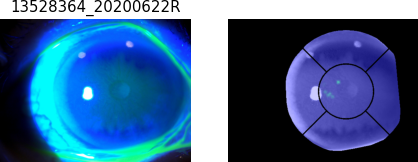

Supplement: S1 Dataset — (ZIP) [file pone.0299776.s002.zip › 13528364_20200622R/13528364_20200622R_whole.png]

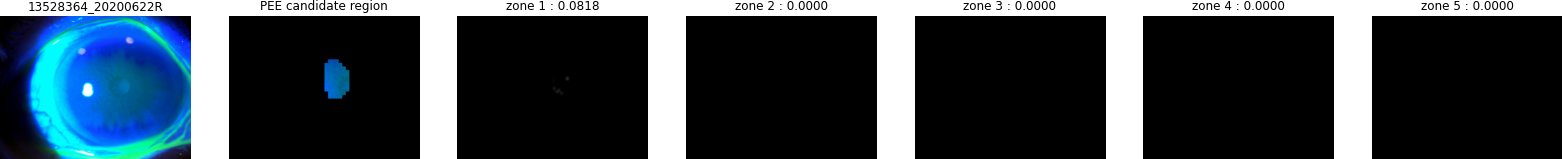

Supplement: S1 Dataset — (ZIP) [file pone.0299776.s002.zip › 13528364_20200622R/13528364_20200622R_zone.png]

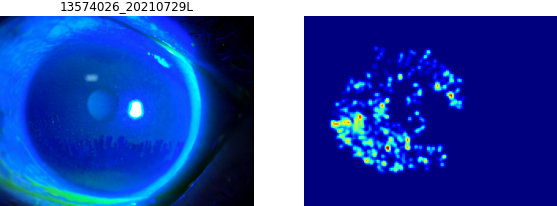

Supplement: S1 Dataset — (ZIP) [file pone.0299776.s002.zip › 13574026_20210729L/13574026_20210729L_densitymap.png]

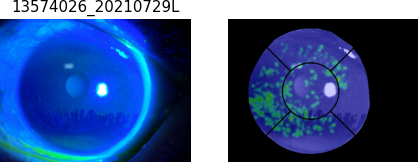

Supplement: S1 Dataset — (ZIP) [file pone.0299776.s002.zip › 13574026_20210729L/13574026_20210729L_whole.png]

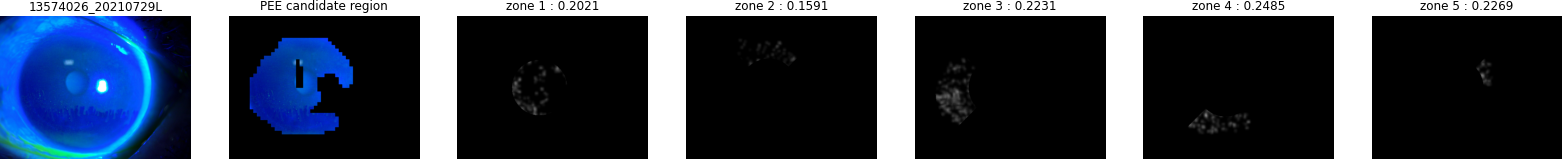

Supplement: S1 Dataset — (ZIP) [file pone.0299776.s002.zip › 13574026_20210729L/13574026_20210729L_zone.png]

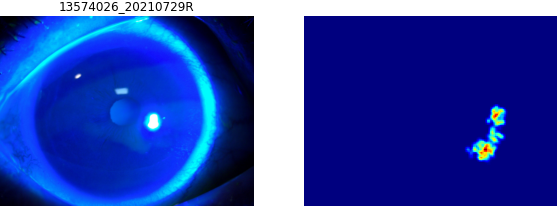

Supplement: S1 Dataset — (ZIP) [file pone.0299776.s002.zip › 13574026_20210729R/13574026_20210729R_densitymap.png]
