# Supplementary figures and images for: Deep learning-based fully automated grading system for dry eye disease severity (part 4 of 6)
Source: PLoS One. 2024 Mar 14;19(3):e0299776. doi: 10.1371/journal.pone.0299776 (PMC10939279; doi:10.1371/journal.pone.0299776)

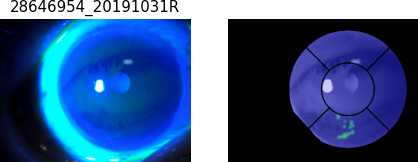

Supplement: S2 Dataset — (ZIP) [file pone.0299776.s003.zip › 28646954_20191031R/28646954_20191031R_whole.png]

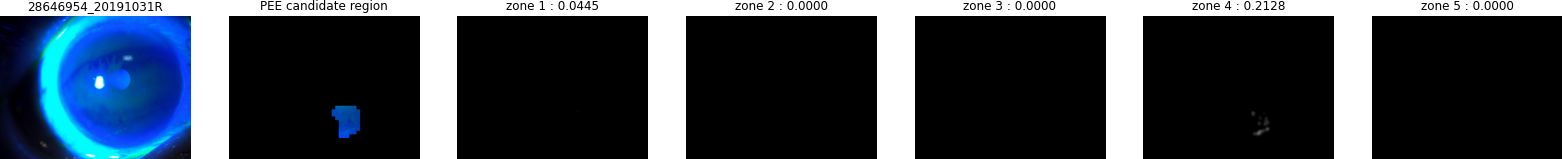

Supplement: S2 Dataset — (ZIP) [file pone.0299776.s003.zip › 28646954_20191031R/28646954_20191031R_zone.png]

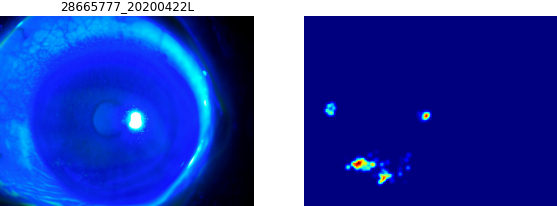

Supplement: S2 Dataset — (ZIP) [file pone.0299776.s003.zip › 28665777_20200422L/28665777_20200422L_densitymap.png]

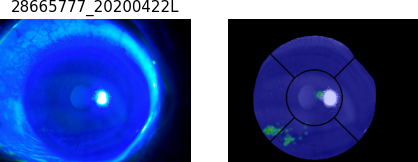

Supplement: S2 Dataset — (ZIP) [file pone.0299776.s003.zip › 28665777_20200422L/28665777_20200422L_whole.png]

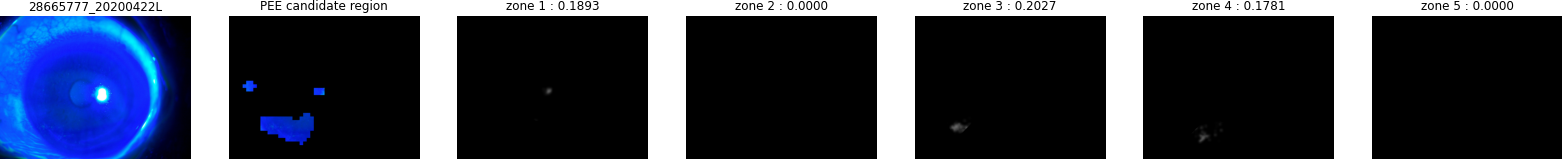

Supplement: S2 Dataset — (ZIP) [file pone.0299776.s003.zip › 28665777_20200422L/28665777_20200422L_zone.png]

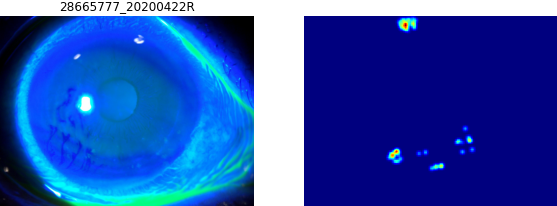

Supplement: S2 Dataset — (ZIP) [file pone.0299776.s003.zip › 28665777_20200422R/28665777_20200422R_densitymap.png]

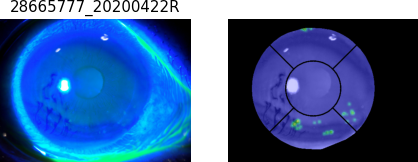

Supplement: S2 Dataset — (ZIP) [file pone.0299776.s003.zip › 28665777_20200422R/28665777_20200422R_whole.png]

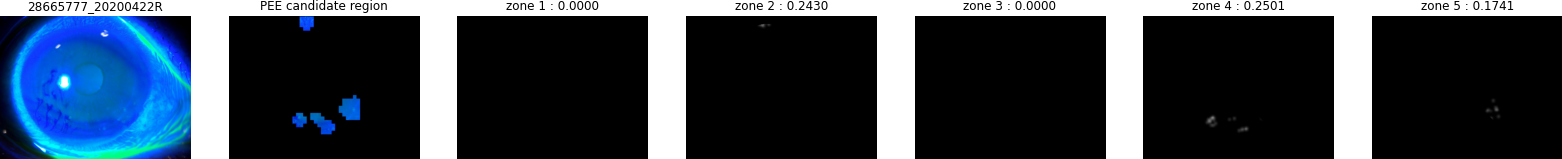

Supplement: S2 Dataset — (ZIP) [file pone.0299776.s003.zip › 28665777_20200422R/28665777_20200422R_zone.png]

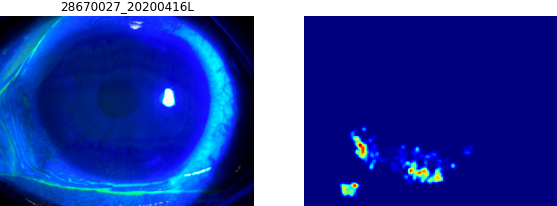

Supplement: S2 Dataset — (ZIP) [file pone.0299776.s003.zip › 28670027_20200416L/28670027_20200416L_densitymap.png]

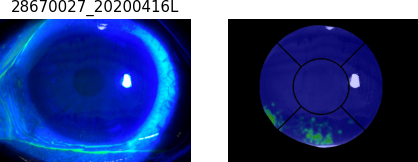

Supplement: S2 Dataset — (ZIP) [file pone.0299776.s003.zip › 28670027_20200416L/28670027_20200416L_whole.png]

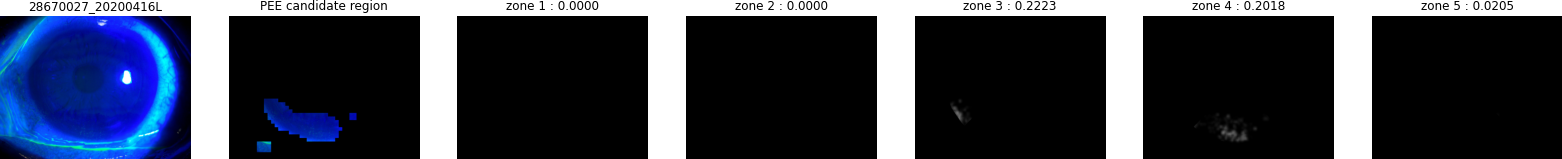

Supplement: S2 Dataset — (ZIP) [file pone.0299776.s003.zip › 28670027_20200416L/28670027_20200416L_zone.png]

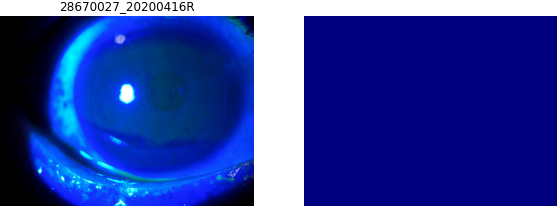

Supplement: S2 Dataset — (ZIP) [file pone.0299776.s003.zip › 28670027_20200416R/28670027_20200416R_densitymap.png]

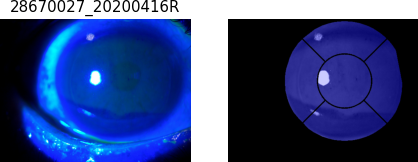

Supplement: S2 Dataset — (ZIP) [file pone.0299776.s003.zip › 28670027_20200416R/28670027_20200416R_whole.png]

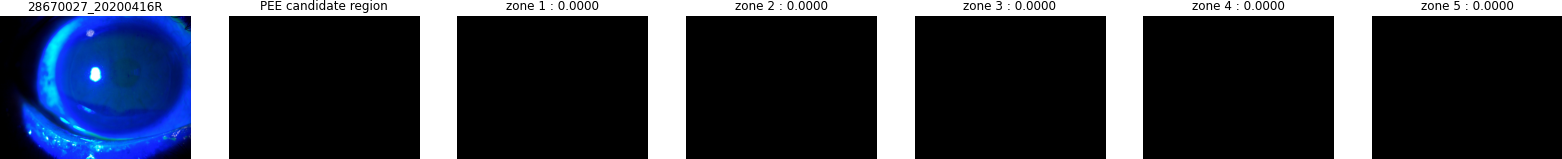

Supplement: S2 Dataset — (ZIP) [file pone.0299776.s003.zip › 28670027_20200416R/28670027_20200416R_zone.png]

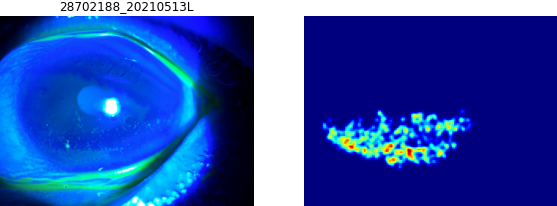

Supplement: S2 Dataset — (ZIP) [file pone.0299776.s003.zip › 28702188_20210513L/28702188_20210513L_densitymap.png]

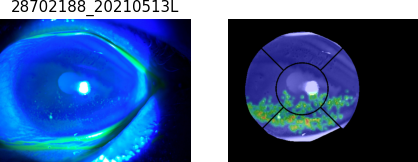

Supplement: S2 Dataset — (ZIP) [file pone.0299776.s003.zip › 28702188_20210513L/28702188_20210513L_whole.png]

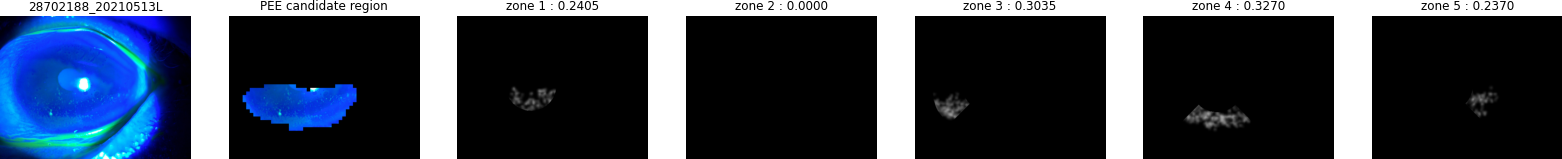

Supplement: S2 Dataset — (ZIP) [file pone.0299776.s003.zip › 28702188_20210513L/28702188_20210513L_zone.png]

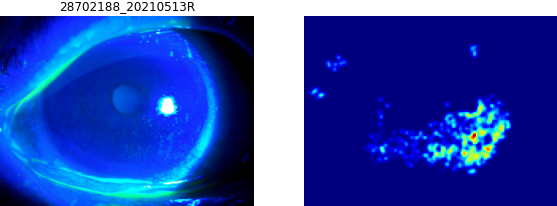

Supplement: S2 Dataset — (ZIP) [file pone.0299776.s003.zip › 28702188_20210513R/28702188_20210513R_densitymap.png]

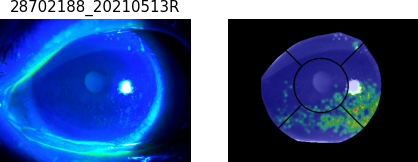

Supplement: S2 Dataset — (ZIP) [file pone.0299776.s003.zip › 28702188_20210513R/28702188_20210513R_whole.png]

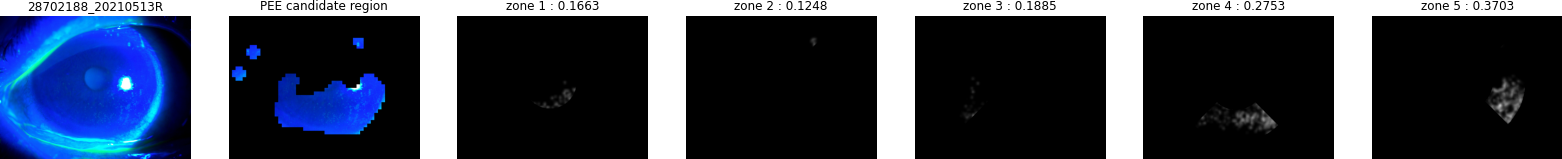

Supplement: S2 Dataset — (ZIP) [file pone.0299776.s003.zip › 28702188_20210513R/28702188_20210513R_zone.png]

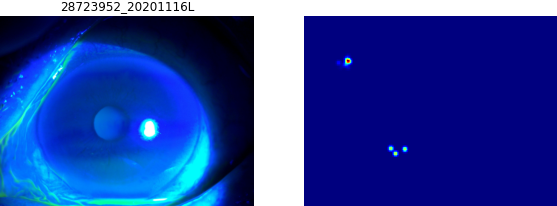

Supplement: S2 Dataset — (ZIP) [file pone.0299776.s003.zip › 28723952_20201116L/28723952_20201116L_densitymap.png]

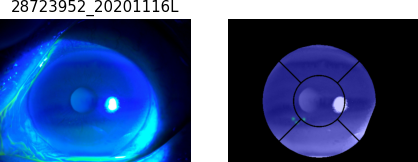

Supplement: S2 Dataset — (ZIP) [file pone.0299776.s003.zip › 28723952_20201116L/28723952_20201116L_whole.png]

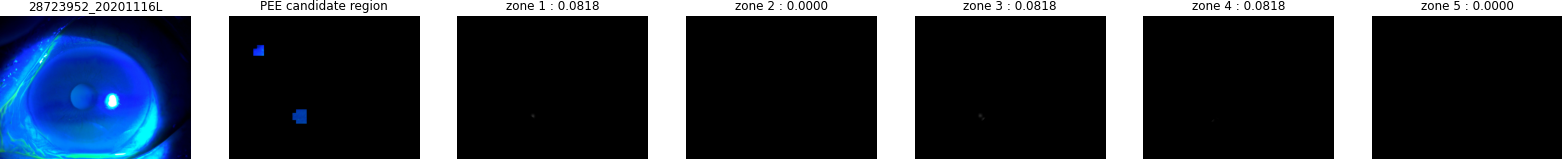

Supplement: S2 Dataset — (ZIP) [file pone.0299776.s003.zip › 28723952_20201116L/28723952_20201116L_zone.png]

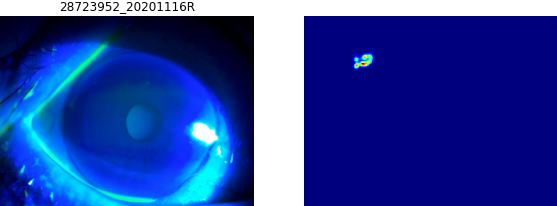

Supplement: S2 Dataset — (ZIP) [file pone.0299776.s003.zip › 28723952_20201116R/28723952_20201116R_densitymap.png]

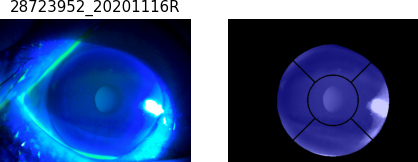

Supplement: S2 Dataset — (ZIP) [file pone.0299776.s003.zip › 28723952_20201116R/28723952_20201116R_whole.png]

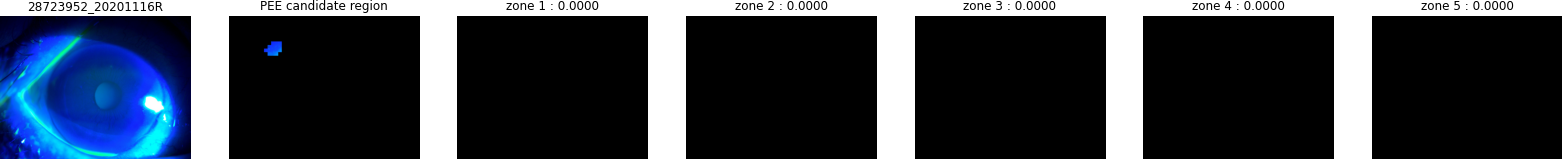

Supplement: S2 Dataset — (ZIP) [file pone.0299776.s003.zip › 28723952_20201116R/28723952_20201116R_zone.png]

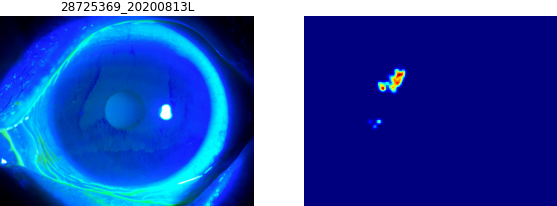

Supplement: S2 Dataset — (ZIP) [file pone.0299776.s003.zip › 28725369_20200813L/28725369_20200813L_densitymap.png]

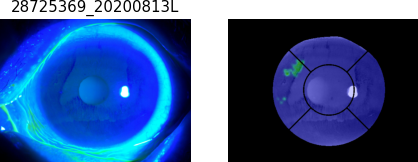

Supplement: S2 Dataset — (ZIP) [file pone.0299776.s003.zip › 28725369_20200813L/28725369_20200813L_whole.png]

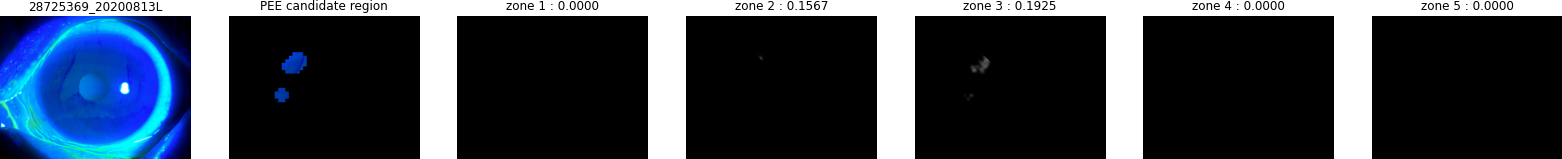

Supplement: S2 Dataset — (ZIP) [file pone.0299776.s003.zip › 28725369_20200813L/28725369_20200813L_zone.png]

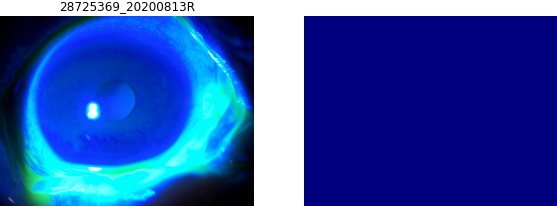

Supplement: S2 Dataset — (ZIP) [file pone.0299776.s003.zip › 28725369_20200813R/28725369_20200813R_densitymap.png]

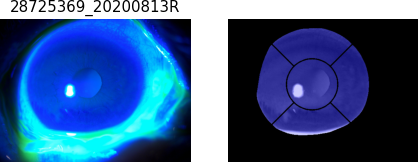

Supplement: S2 Dataset — (ZIP) [file pone.0299776.s003.zip › 28725369_20200813R/28725369_20200813R_whole.png]

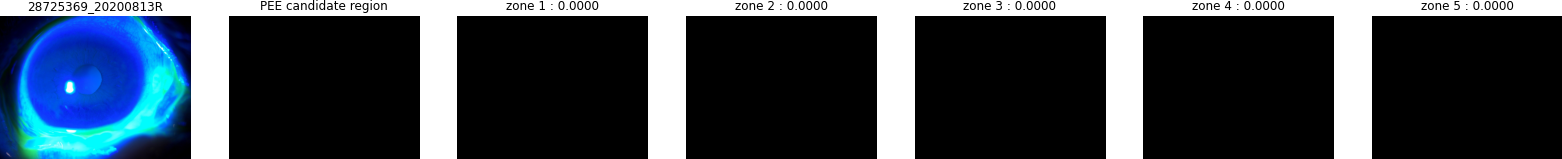

Supplement: S2 Dataset — (ZIP) [file pone.0299776.s003.zip › 28725369_20200813R/28725369_20200813R_zone.png]

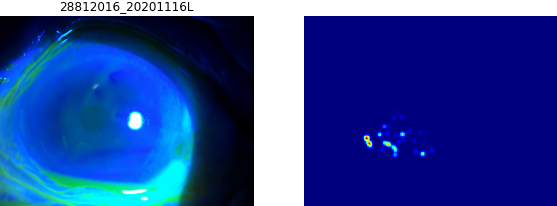

Supplement: S2 Dataset — (ZIP) [file pone.0299776.s003.zip › 28812016_20201116L/28812016_20201116L_densitymap.png]

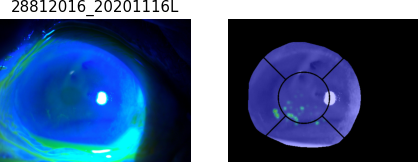

Supplement: S2 Dataset — (ZIP) [file pone.0299776.s003.zip › 28812016_20201116L/28812016_20201116L_whole.png]

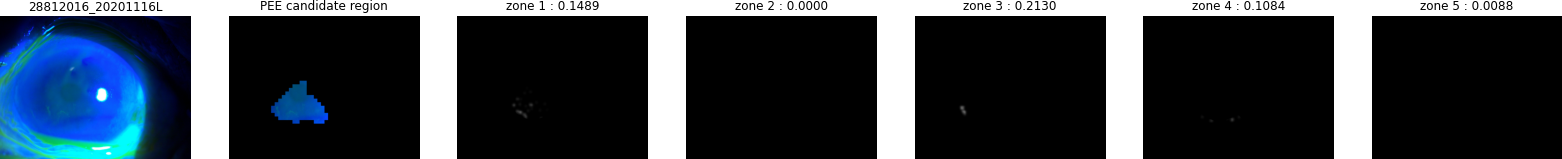

Supplement: S2 Dataset — (ZIP) [file pone.0299776.s003.zip › 28812016_20201116L/28812016_20201116L_zone.png]

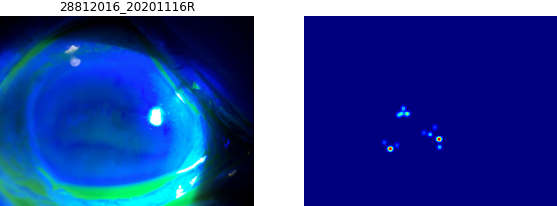

Supplement: S2 Dataset — (ZIP) [file pone.0299776.s003.zip › 28812016_20201116R/28812016_20201116R_densitymap.png]

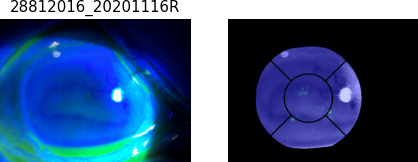

Supplement: S2 Dataset — (ZIP) [file pone.0299776.s003.zip › 28812016_20201116R/28812016_20201116R_whole.png]

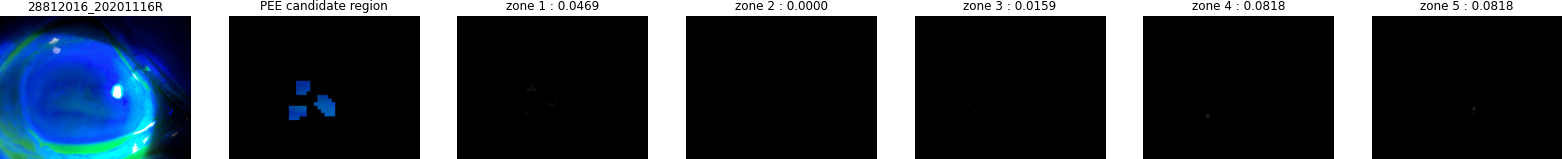

Supplement: S2 Dataset — (ZIP) [file pone.0299776.s003.zip › 28812016_20201116R/28812016_20201116R_zone.png]

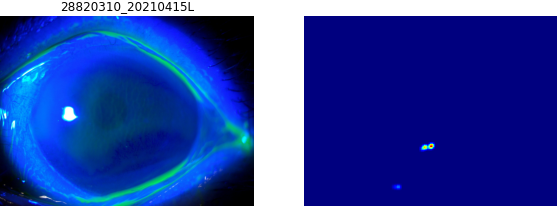

Supplement: S2 Dataset — (ZIP) [file pone.0299776.s003.zip › 28820310_20210415L/28820310_20210415L_densitymap.png]

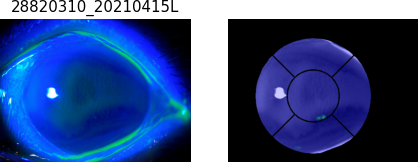

Supplement: S2 Dataset — (ZIP) [file pone.0299776.s003.zip › 28820310_20210415L/28820310_20210415L_whole.png]

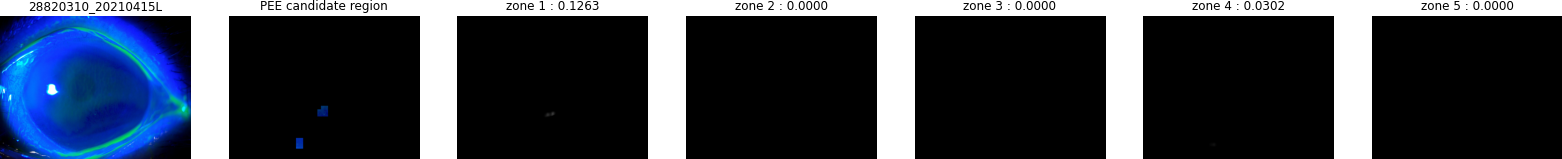

Supplement: S2 Dataset — (ZIP) [file pone.0299776.s003.zip › 28820310_20210415L/28820310_20210415L_zone.png]

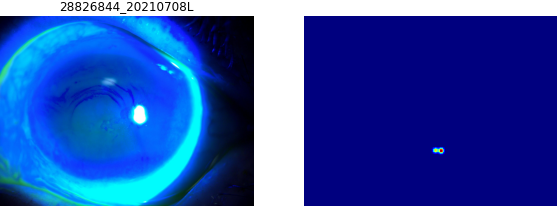

Supplement: S2 Dataset — (ZIP) [file pone.0299776.s003.zip › 28826844_20210708L/28826844_20210708L_densitymap.png]

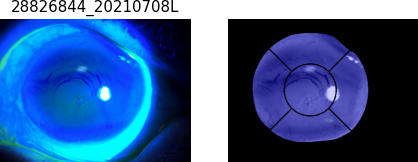

Supplement: S2 Dataset — (ZIP) [file pone.0299776.s003.zip › 28826844_20210708L/28826844_20210708L_whole.png]

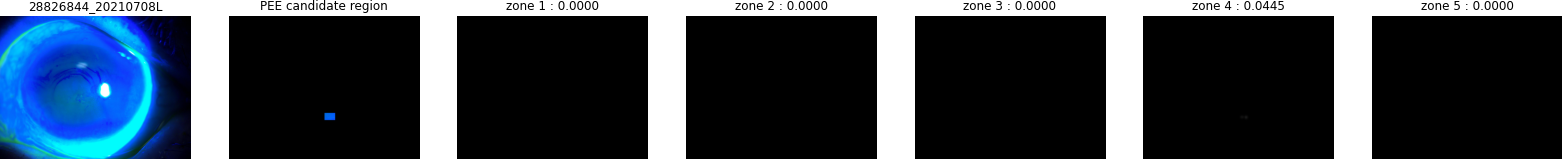

Supplement: S2 Dataset — (ZIP) [file pone.0299776.s003.zip › 28826844_20210708L/28826844_20210708L_zone.png]

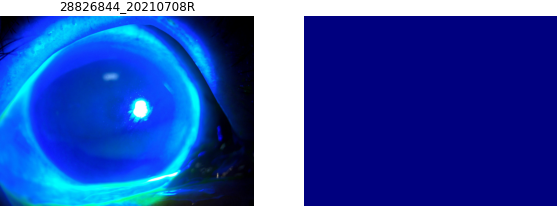

Supplement: S2 Dataset — (ZIP) [file pone.0299776.s003.zip › 28826844_20210708R/28826844_20210708R_densitymap.png]

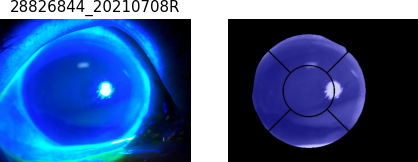

Supplement: S2 Dataset — (ZIP) [file pone.0299776.s003.zip › 28826844_20210708R/28826844_20210708R_whole.png]

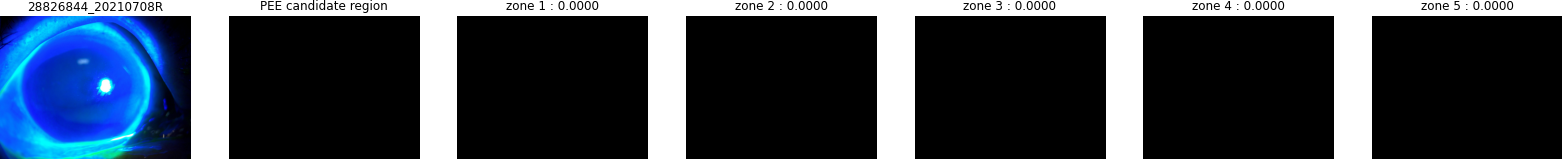

Supplement: S2 Dataset — (ZIP) [file pone.0299776.s003.zip › 28826844_20210708R/28826844_20210708R_zone.png]

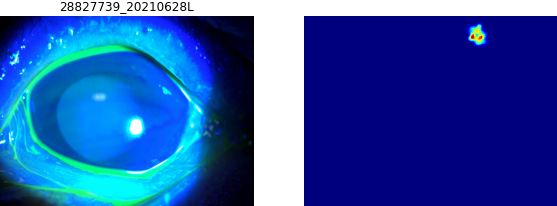

Supplement: S2 Dataset — (ZIP) [file pone.0299776.s003.zip › 28827739_20210628L/28827739_20210628L_densitymap.png]

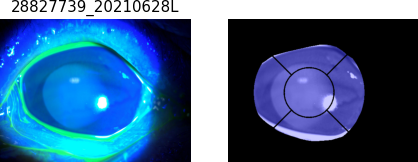

Supplement: S2 Dataset — (ZIP) [file pone.0299776.s003.zip › 28827739_20210628L/28827739_20210628L_whole.png]

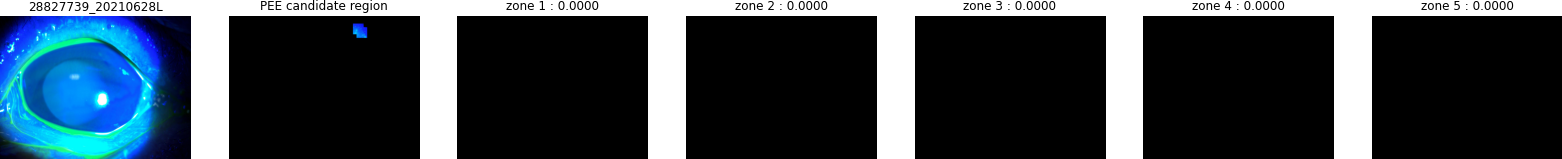

Supplement: S2 Dataset — (ZIP) [file pone.0299776.s003.zip › 28827739_20210628L/28827739_20210628L_zone.png]

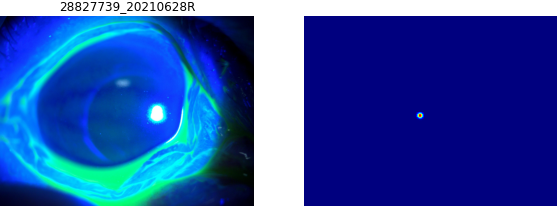

Supplement: S2 Dataset — (ZIP) [file pone.0299776.s003.zip › 28827739_20210628R/28827739_20210628R_densitymap.png]

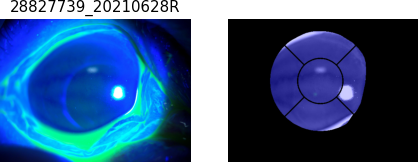

Supplement: S2 Dataset — (ZIP) [file pone.0299776.s003.zip › 28827739_20210628R/28827739_20210628R_whole.png]

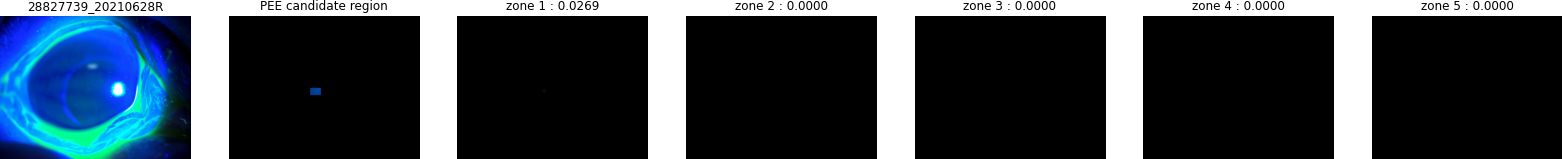

Supplement: S2 Dataset — (ZIP) [file pone.0299776.s003.zip › 28827739_20210628R/28827739_20210628R_zone.png]

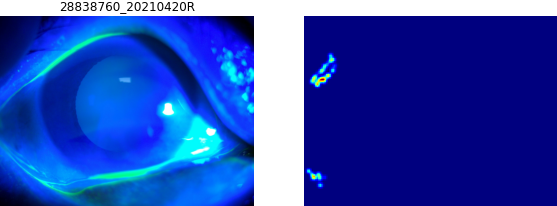

Supplement: S2 Dataset — (ZIP) [file pone.0299776.s003.zip › 28838760_20210420R/28838760_20210420R_densitymap.png]

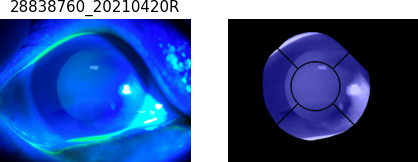

Supplement: S2 Dataset — (ZIP) [file pone.0299776.s003.zip › 28838760_20210420R/28838760_20210420R_whole.png]

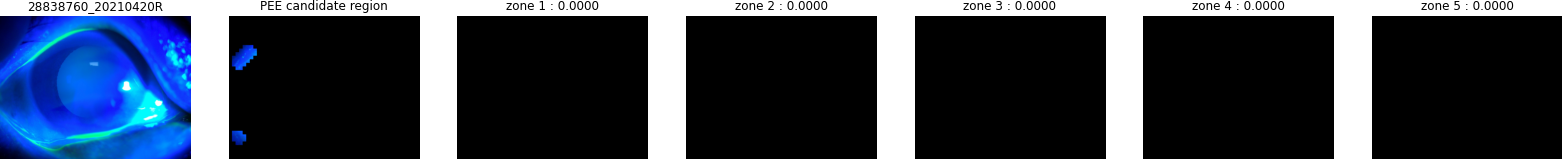

Supplement: S2 Dataset — (ZIP) [file pone.0299776.s003.zip › 28838760_20210420R/28838760_20210420R_zone.png]

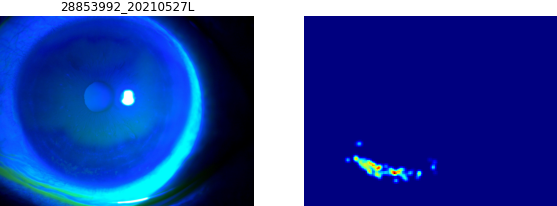

Supplement: S2 Dataset — (ZIP) [file pone.0299776.s003.zip › 28853992_20210527L/28853992_20210527L_densitymap.png]

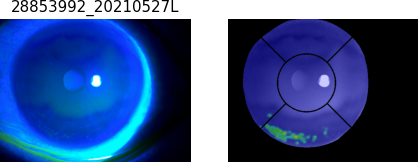

Supplement: S2 Dataset — (ZIP) [file pone.0299776.s003.zip › 28853992_20210527L/28853992_20210527L_whole.png]

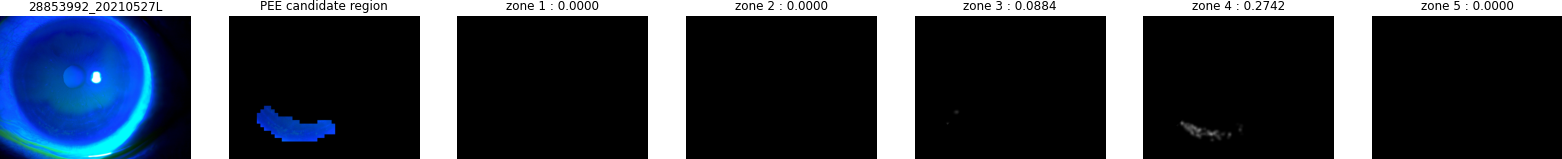

Supplement: S2 Dataset — (ZIP) [file pone.0299776.s003.zip › 28853992_20210527L/28853992_20210527L_zone.png]

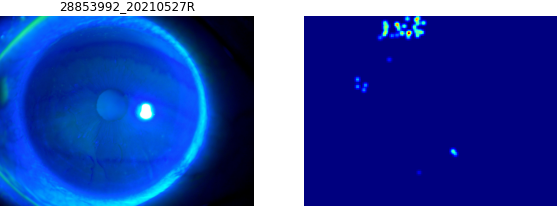

Supplement: S2 Dataset — (ZIP) [file pone.0299776.s003.zip › 28853992_20210527R/28853992_20210527R_densitymap.png]

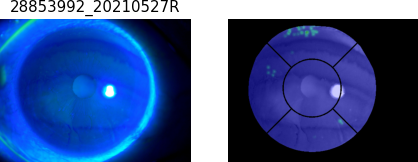

Supplement: S2 Dataset — (ZIP) [file pone.0299776.s003.zip › 28853992_20210527R/28853992_20210527R_whole.png]

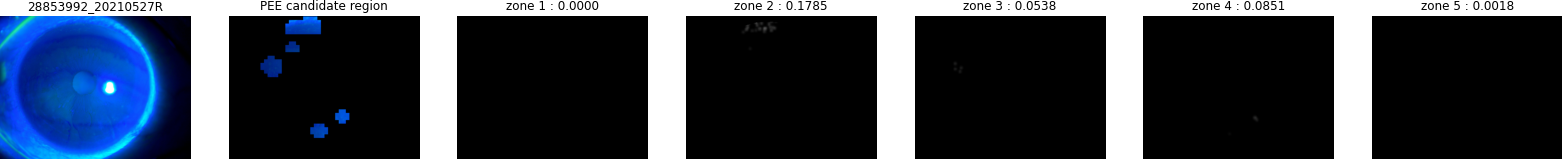

Supplement: S2 Dataset — (ZIP) [file pone.0299776.s003.zip › 28853992_20210527R/28853992_20210527R_zone.png]

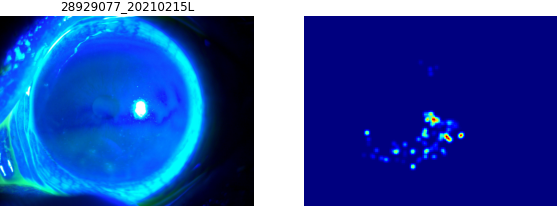

Supplement: S2 Dataset — (ZIP) [file pone.0299776.s003.zip › 28929077_20210215L/28929077_20210215L_densitymap.png]

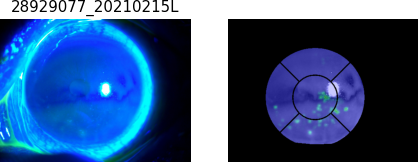

Supplement: S2 Dataset — (ZIP) [file pone.0299776.s003.zip › 28929077_20210215L/28929077_20210215L_whole.png]

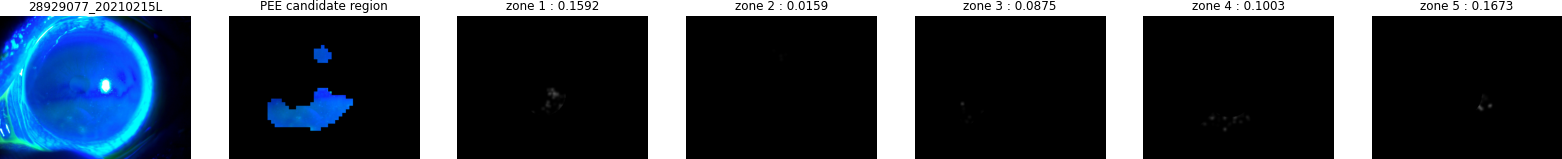

Supplement: S2 Dataset — (ZIP) [file pone.0299776.s003.zip › 28929077_20210215L/28929077_20210215L_zone.png]

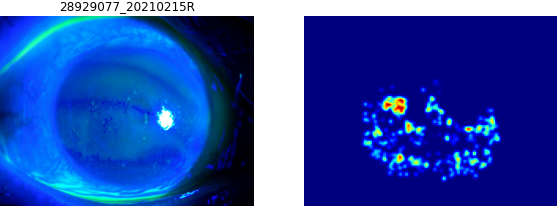

Supplement: S2 Dataset — (ZIP) [file pone.0299776.s003.zip › 28929077_20210215R/28929077_20210215R_densitymap.png]

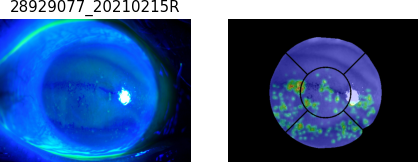

Supplement: S2 Dataset — (ZIP) [file pone.0299776.s003.zip › 28929077_20210215R/28929077_20210215R_whole.png]

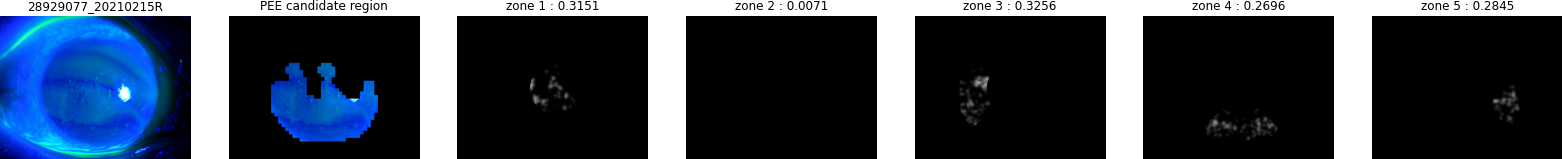

Supplement: S2 Dataset — (ZIP) [file pone.0299776.s003.zip › 28929077_20210215R/28929077_20210215R_zone.png]

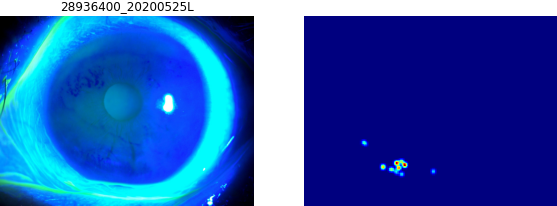

Supplement: S2 Dataset — (ZIP) [file pone.0299776.s003.zip › 28936400_20200525L/28936400_20200525L_densitymap.png]

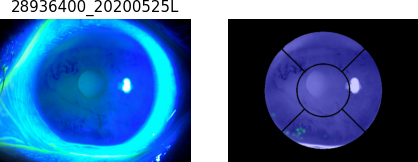

Supplement: S2 Dataset — (ZIP) [file pone.0299776.s003.zip › 28936400_20200525L/28936400_20200525L_whole.png]

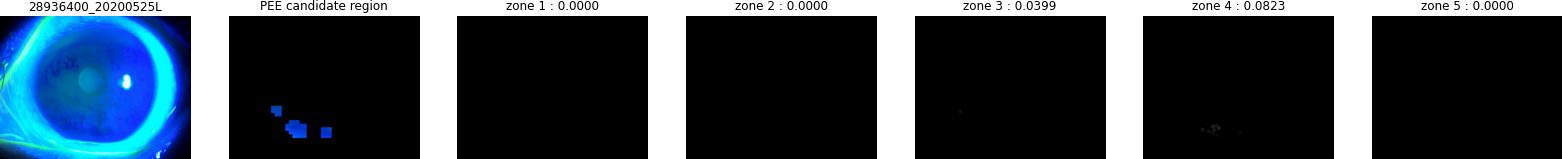

Supplement: S2 Dataset — (ZIP) [file pone.0299776.s003.zip › 28936400_20200525L/28936400_20200525L_zone.png]

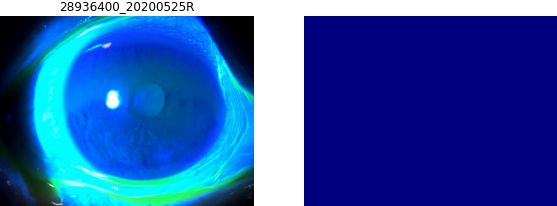

Supplement: S2 Dataset — (ZIP) [file pone.0299776.s003.zip › 28936400_20200525R/28936400_20200525R_densitymap.png]

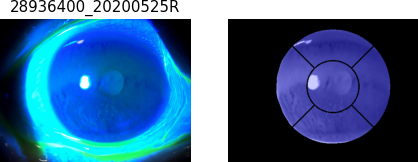

Supplement: S2 Dataset — (ZIP) [file pone.0299776.s003.zip › 28936400_20200525R/28936400_20200525R_whole.png]

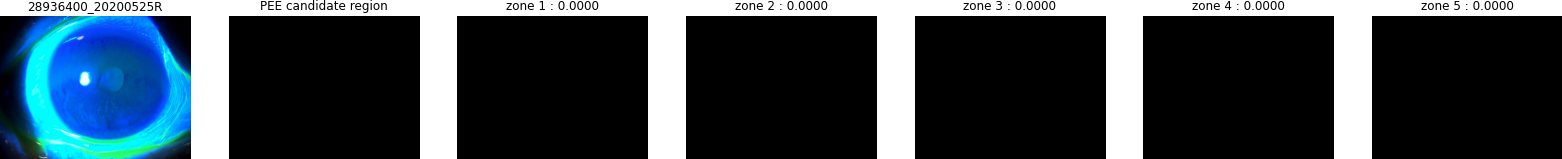

Supplement: S2 Dataset — (ZIP) [file pone.0299776.s003.zip › 28936400_20200525R/28936400_20200525R_zone.png]

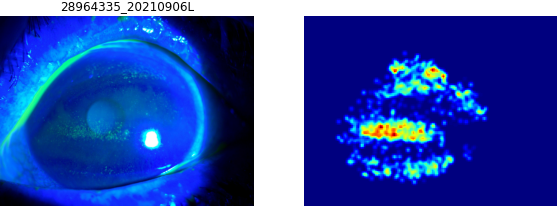

Supplement: S2 Dataset — (ZIP) [file pone.0299776.s003.zip › 28964335_20210906L/28964335_20210906L_densitymap.png]

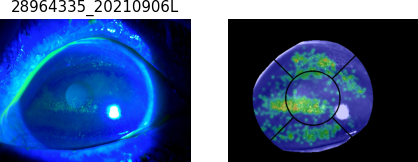

Supplement: S2 Dataset — (ZIP) [file pone.0299776.s003.zip › 28964335_20210906L/28964335_20210906L_whole.png]

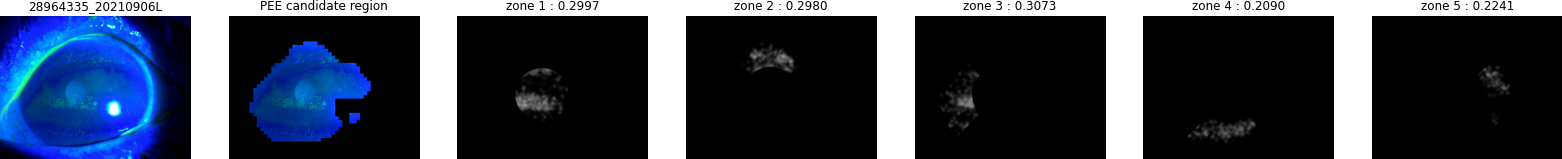

Supplement: S2 Dataset — (ZIP) [file pone.0299776.s003.zip › 28964335_20210906L/28964335_20210906L_zone.png]

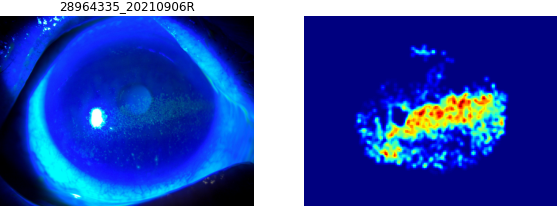

Supplement: S2 Dataset — (ZIP) [file pone.0299776.s003.zip › 28964335_20210906R/28964335_20210906R_densitymap.png]

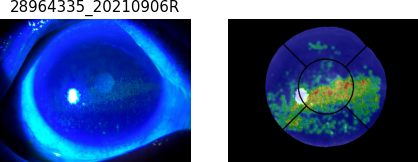

Supplement: S2 Dataset — (ZIP) [file pone.0299776.s003.zip › 28964335_20210906R/28964335_20210906R_whole.png]

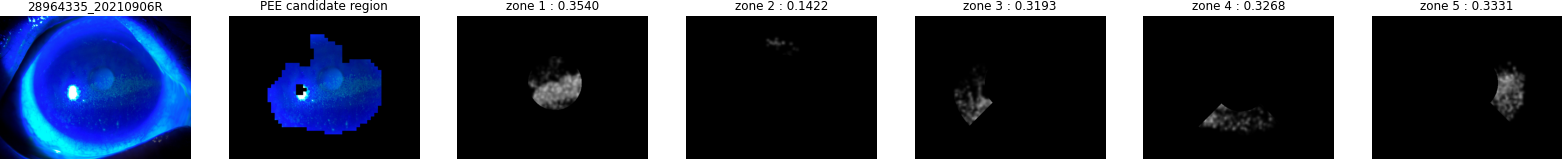

Supplement: S2 Dataset — (ZIP) [file pone.0299776.s003.zip › 28964335_20210906R/28964335_20210906R_zone.png]

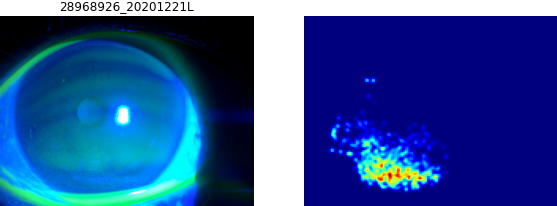

Supplement: S2 Dataset — (ZIP) [file pone.0299776.s003.zip › 28968926_20201221L/28968926_20201221L_densitymap.png]

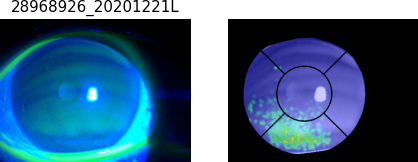

Supplement: S2 Dataset — (ZIP) [file pone.0299776.s003.zip › 28968926_20201221L/28968926_20201221L_whole.png]

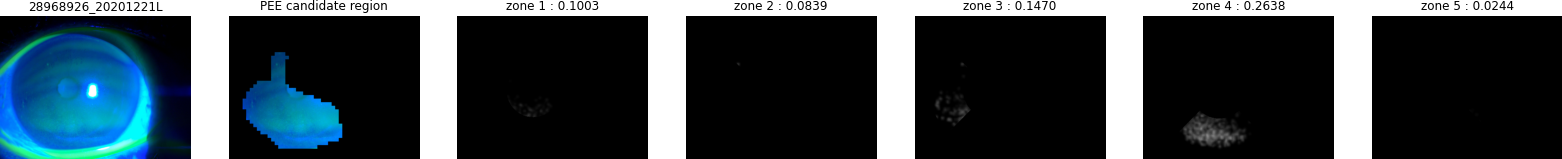

Supplement: S2 Dataset — (ZIP) [file pone.0299776.s003.zip › 28968926_20201221L/28968926_20201221L_zone.png]

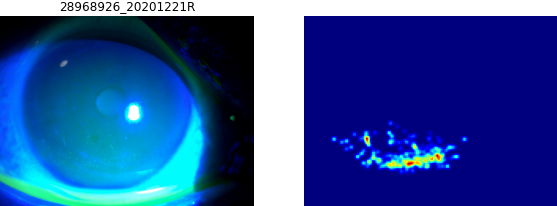

Supplement: S2 Dataset — (ZIP) [file pone.0299776.s003.zip › 28968926_20201221R/28968926_20201221R_densitymap.png]

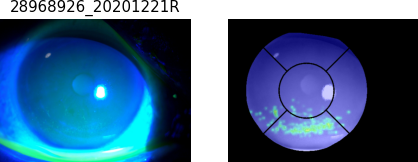

Supplement: S2 Dataset — (ZIP) [file pone.0299776.s003.zip › 28968926_20201221R/28968926_20201221R_whole.png]

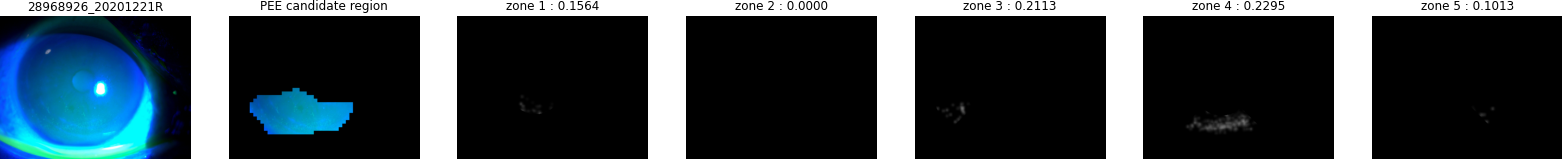

Supplement: S2 Dataset — (ZIP) [file pone.0299776.s003.zip › 28968926_20201221R/28968926_20201221R_zone.png]

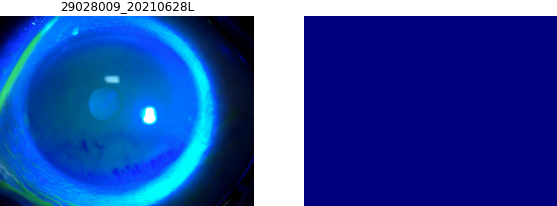

Supplement: S2 Dataset — (ZIP) [file pone.0299776.s003.zip › 29028009_20210628L/29028009_20210628L_densitymap.png]

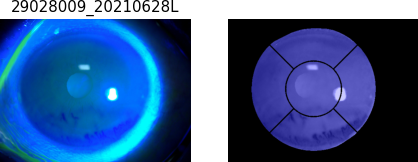

Supplement: S2 Dataset — (ZIP) [file pone.0299776.s003.zip › 29028009_20210628L/29028009_20210628L_whole.png]

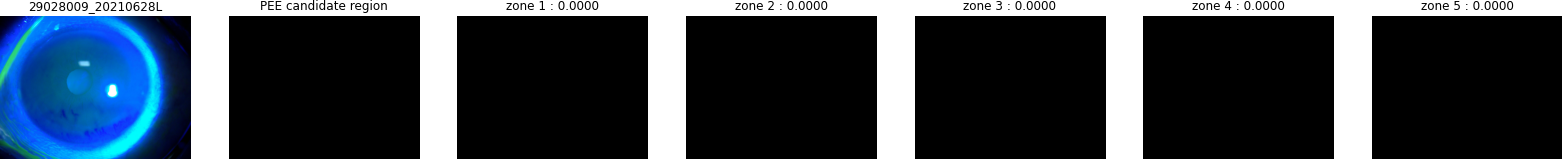

Supplement: S2 Dataset — (ZIP) [file pone.0299776.s003.zip › 29028009_20210628L/29028009_20210628L_zone.png]

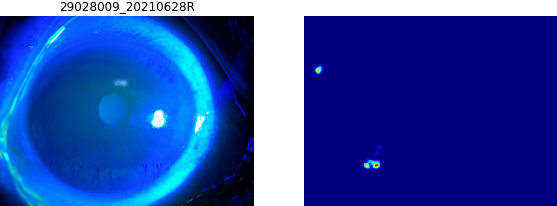

Supplement: S2 Dataset — (ZIP) [file pone.0299776.s003.zip › 29028009_20210628R/29028009_20210628R_densitymap.png]

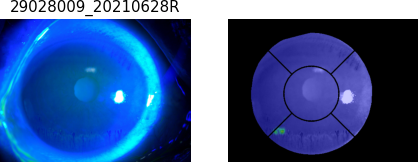

Supplement: S2 Dataset — (ZIP) [file pone.0299776.s003.zip › 29028009_20210628R/29028009_20210628R_whole.png]

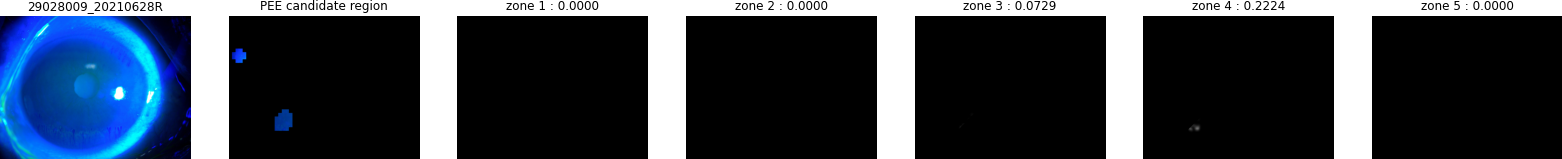

Supplement: S2 Dataset — (ZIP) [file pone.0299776.s003.zip › 29028009_20210628R/29028009_20210628R_zone.png]

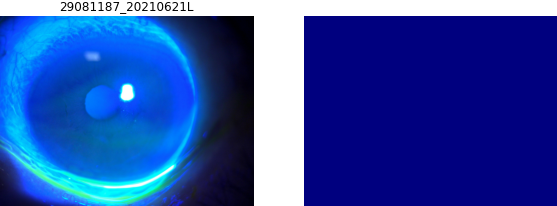

Supplement: S2 Dataset — (ZIP) [file pone.0299776.s003.zip › 29081187_20210621L/29081187_20210621L_densitymap.png]

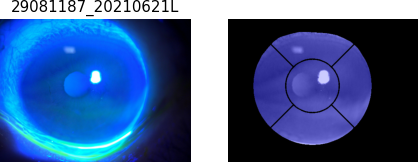

Supplement: S2 Dataset — (ZIP) [file pone.0299776.s003.zip › 29081187_20210621L/29081187_20210621L_whole.png]

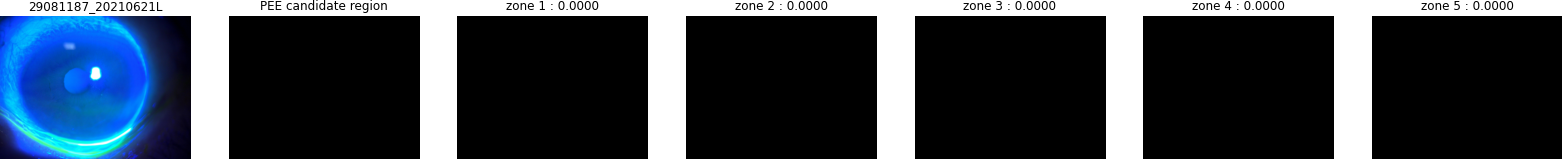

Supplement: S2 Dataset — (ZIP) [file pone.0299776.s003.zip › 29081187_20210621L/29081187_20210621L_zone.png]

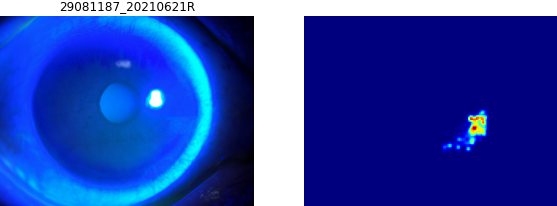

Supplement: S2 Dataset — (ZIP) [file pone.0299776.s003.zip › 29081187_20210621R/29081187_20210621R_densitymap.png]

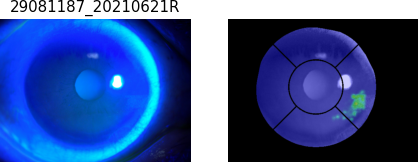

Supplement: S2 Dataset — (ZIP) [file pone.0299776.s003.zip › 29081187_20210621R/29081187_20210621R_whole.png]

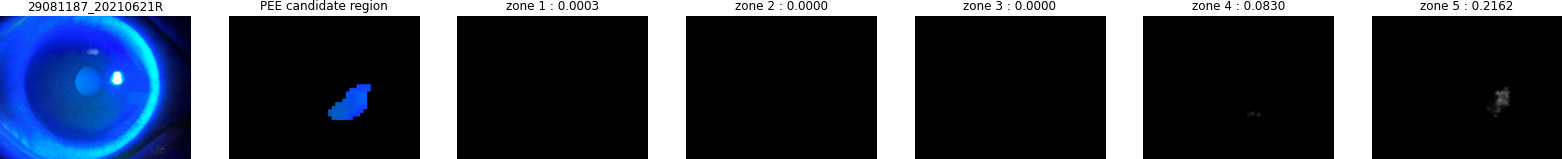

Supplement: S2 Dataset — (ZIP) [file pone.0299776.s003.zip › 29081187_20210621R/29081187_20210621R_zone.png]

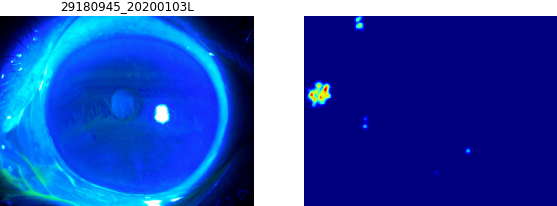

Supplement: S2 Dataset — (ZIP) [file pone.0299776.s003.zip › 29180945_20200103L/29180945_20200103L_densitymap.png]

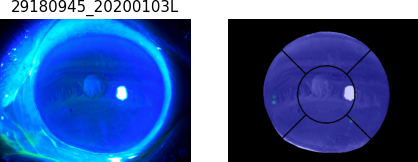

Supplement: S2 Dataset — (ZIP) [file pone.0299776.s003.zip › 29180945_20200103L/29180945_20200103L_whole.png]
