# Supplementary figures and images for: Deep learning-based fully automated grading system for dry eye disease severity (part 3 of 6)
Source: PLoS One. 2024 Mar 14;19(3):e0299776. doi: 10.1371/journal.pone.0299776 (PMC10939279; doi:10.1371/journal.pone.0299776)

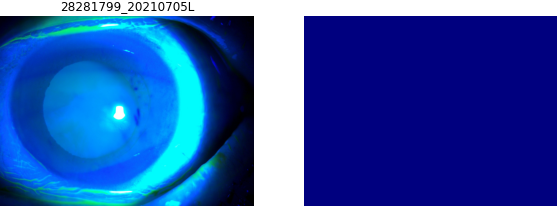

Supplement: S2 Dataset — (ZIP) [file pone.0299776.s003.zip › 28281799_20210705L/28281799_20210705L_densitymap.png]

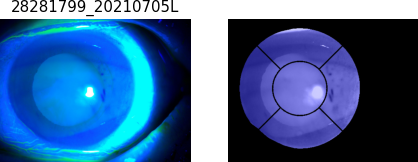

Supplement: S2 Dataset — (ZIP) [file pone.0299776.s003.zip › 28281799_20210705L/28281799_20210705L_whole.png]

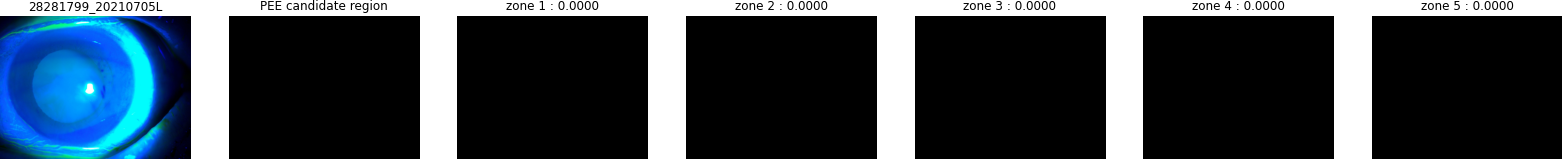

Supplement: S2 Dataset — (ZIP) [file pone.0299776.s003.zip › 28281799_20210705L/28281799_20210705L_zone.png]

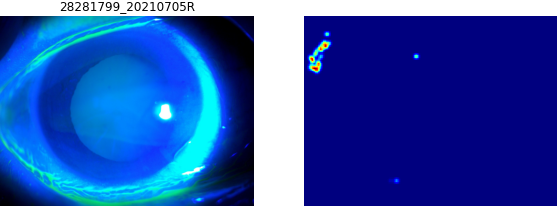

Supplement: S2 Dataset — (ZIP) [file pone.0299776.s003.zip › 28281799_20210705R/28281799_20210705R_densitymap.png]

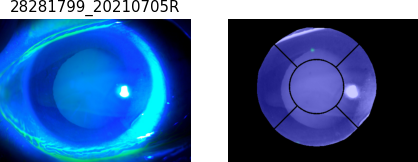

Supplement: S2 Dataset — (ZIP) [file pone.0299776.s003.zip › 28281799_20210705R/28281799_20210705R_whole.png]

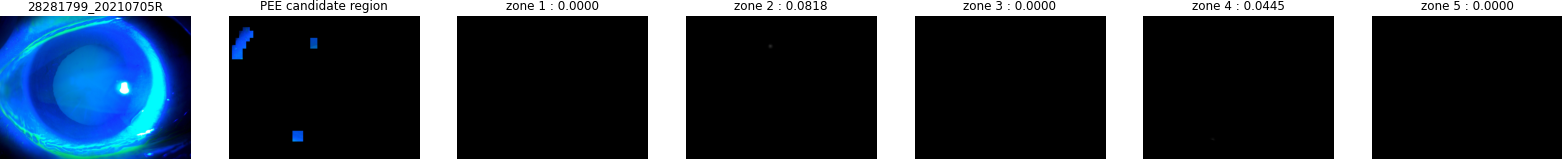

Supplement: S2 Dataset — (ZIP) [file pone.0299776.s003.zip › 28281799_20210705R/28281799_20210705R_zone.png]

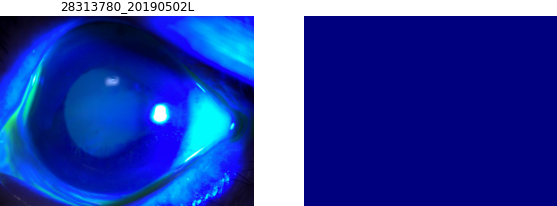

Supplement: S2 Dataset — (ZIP) [file pone.0299776.s003.zip › 28313780_20190502L/28313780_20190502L_densitymap.png]

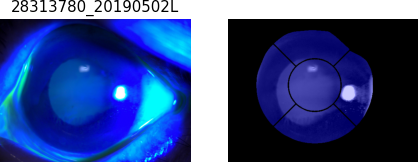

Supplement: S2 Dataset — (ZIP) [file pone.0299776.s003.zip › 28313780_20190502L/28313780_20190502L_whole.png]

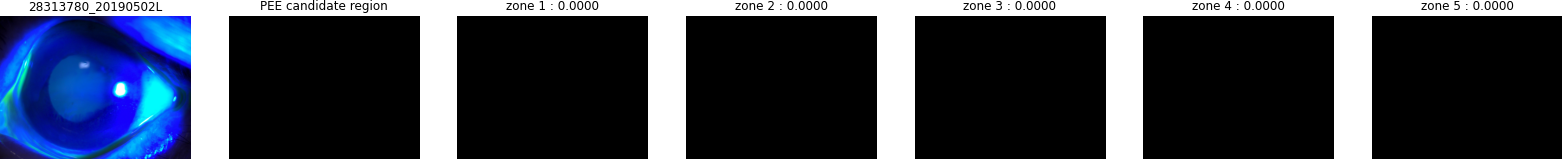

Supplement: S2 Dataset — (ZIP) [file pone.0299776.s003.zip › 28313780_20190502L/28313780_20190502L_zone.png]

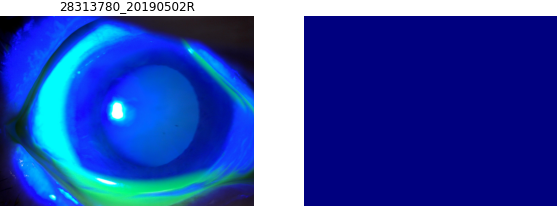

Supplement: S2 Dataset — (ZIP) [file pone.0299776.s003.zip › 28313780_20190502R/28313780_20190502R_densitymap.png]

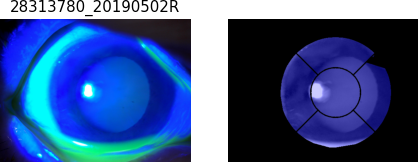

Supplement: S2 Dataset — (ZIP) [file pone.0299776.s003.zip › 28313780_20190502R/28313780_20190502R_whole.png]

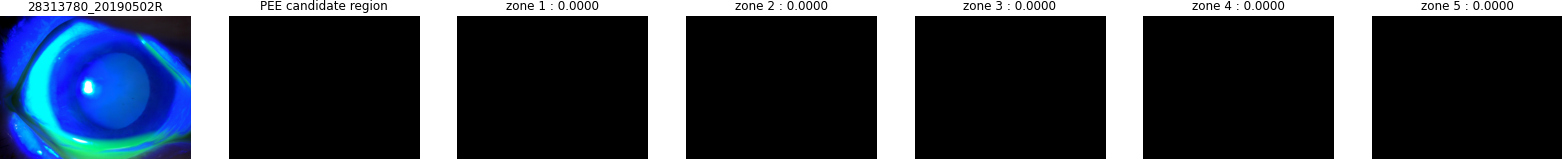

Supplement: S2 Dataset — (ZIP) [file pone.0299776.s003.zip › 28313780_20190502R/28313780_20190502R_zone.png]

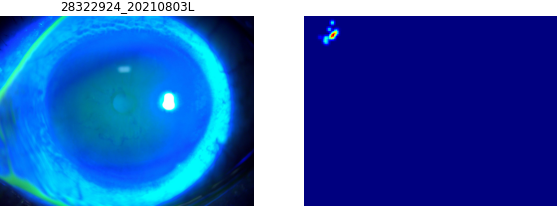

Supplement: S2 Dataset — (ZIP) [file pone.0299776.s003.zip › 28322924_20210803L/28322924_20210803L_densitymap.png]

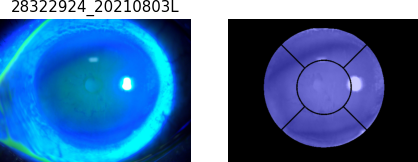

Supplement: S2 Dataset — (ZIP) [file pone.0299776.s003.zip › 28322924_20210803L/28322924_20210803L_whole.png]

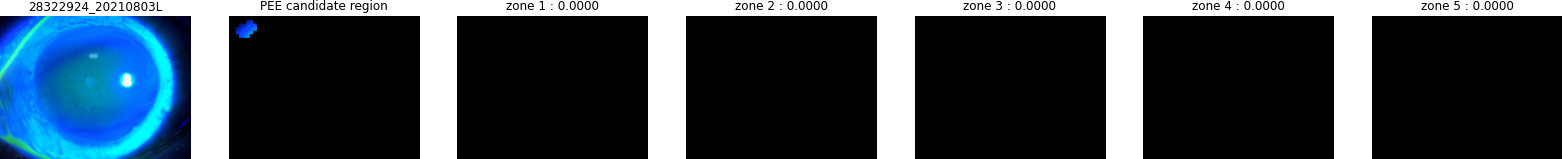

Supplement: S2 Dataset — (ZIP) [file pone.0299776.s003.zip › 28322924_20210803L/28322924_20210803L_zone.png]

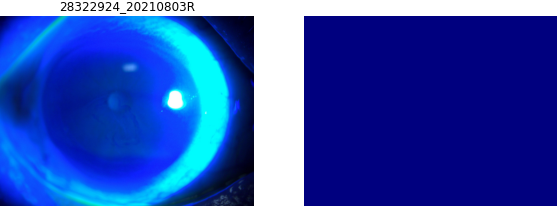

Supplement: S2 Dataset — (ZIP) [file pone.0299776.s003.zip › 28322924_20210803R/28322924_20210803R_densitymap.png]

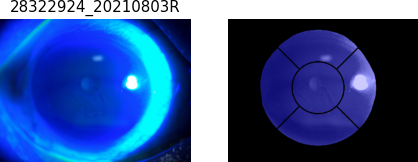

Supplement: S2 Dataset — (ZIP) [file pone.0299776.s003.zip › 28322924_20210803R/28322924_20210803R_whole.png]

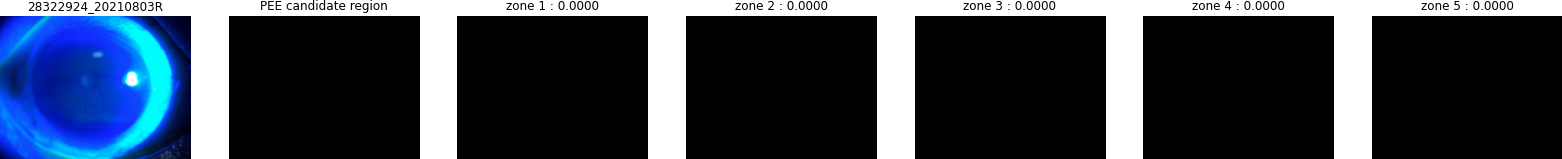

Supplement: S2 Dataset — (ZIP) [file pone.0299776.s003.zip › 28322924_20210803R/28322924_20210803R_zone.png]

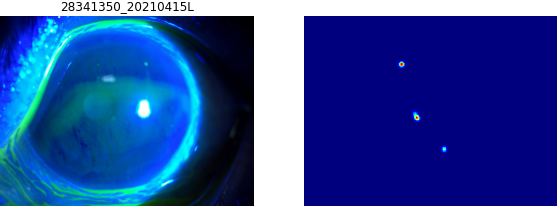

Supplement: S2 Dataset — (ZIP) [file pone.0299776.s003.zip › 28341350_20210415L/28341350_20210415L_densitymap.png]

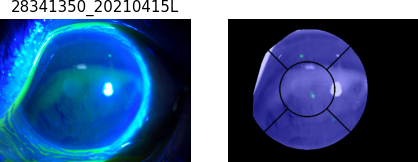

Supplement: S2 Dataset — (ZIP) [file pone.0299776.s003.zip › 28341350_20210415L/28341350_20210415L_whole.png]

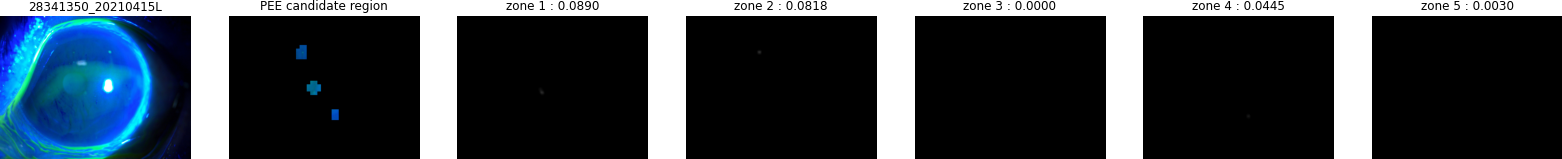

Supplement: S2 Dataset — (ZIP) [file pone.0299776.s003.zip › 28341350_20210415L/28341350_20210415L_zone.png]

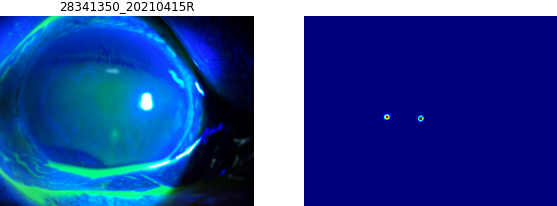

Supplement: S2 Dataset — (ZIP) [file pone.0299776.s003.zip › 28341350_20210415R/28341350_20210415R_densitymap.png]

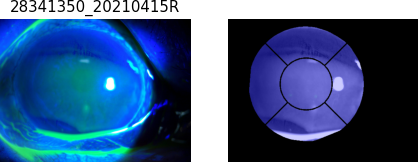

Supplement: S2 Dataset — (ZIP) [file pone.0299776.s003.zip › 28341350_20210415R/28341350_20210415R_whole.png]

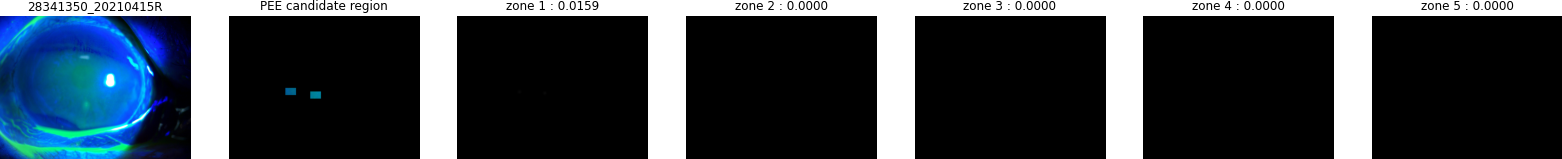

Supplement: S2 Dataset — (ZIP) [file pone.0299776.s003.zip › 28341350_20210415R/28341350_20210415R_zone.png]

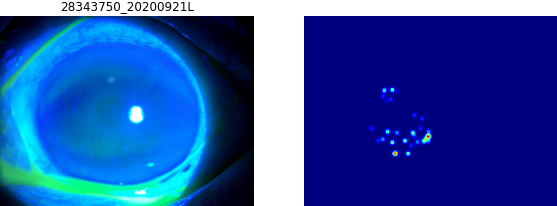

Supplement: S2 Dataset — (ZIP) [file pone.0299776.s003.zip › 28343750_20200921L/28343750_20200921L_densitymap.png]

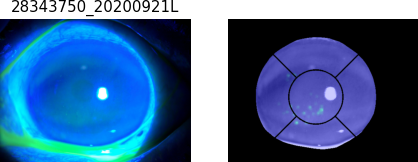

Supplement: S2 Dataset — (ZIP) [file pone.0299776.s003.zip › 28343750_20200921L/28343750_20200921L_whole.png]

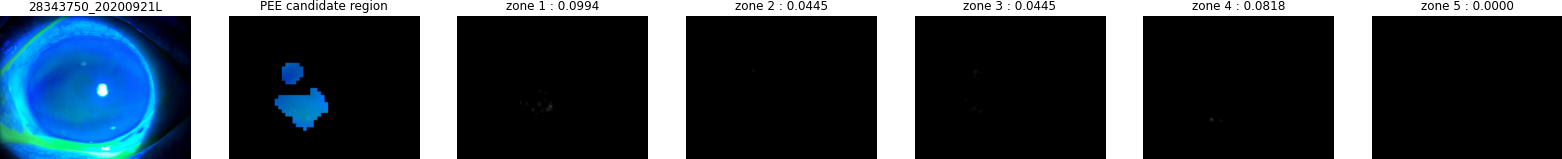

Supplement: S2 Dataset — (ZIP) [file pone.0299776.s003.zip › 28343750_20200921L/28343750_20200921L_zone.png]

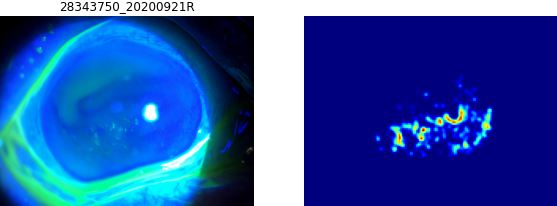

Supplement: S2 Dataset — (ZIP) [file pone.0299776.s003.zip › 28343750_20200921R/28343750_20200921R_densitymap.png]

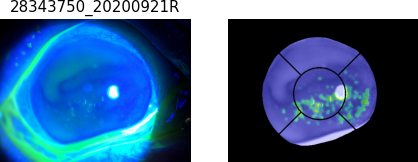

Supplement: S2 Dataset — (ZIP) [file pone.0299776.s003.zip › 28343750_20200921R/28343750_20200921R_whole.png]

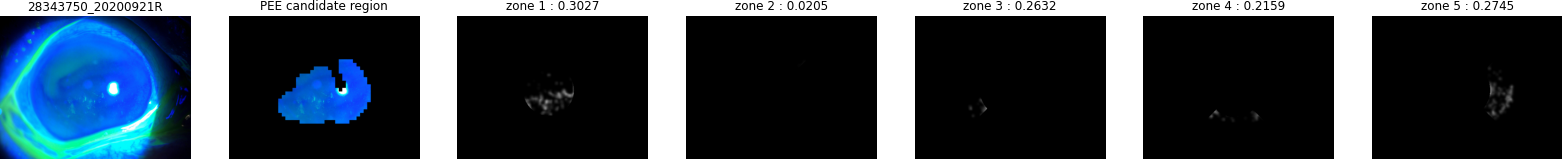

Supplement: S2 Dataset — (ZIP) [file pone.0299776.s003.zip › 28343750_20200921R/28343750_20200921R_zone.png]

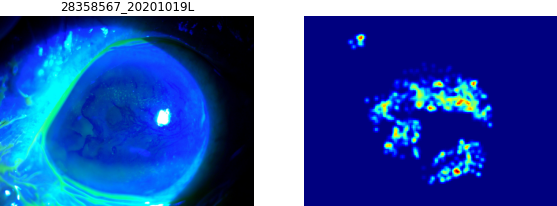

Supplement: S2 Dataset — (ZIP) [file pone.0299776.s003.zip › 28358567_20201019L/28358567_20201019L_densitymap.png]

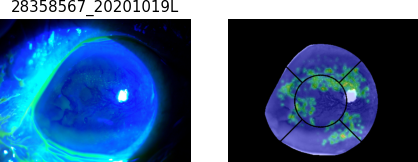

Supplement: S2 Dataset — (ZIP) [file pone.0299776.s003.zip › 28358567_20201019L/28358567_20201019L_whole.png]

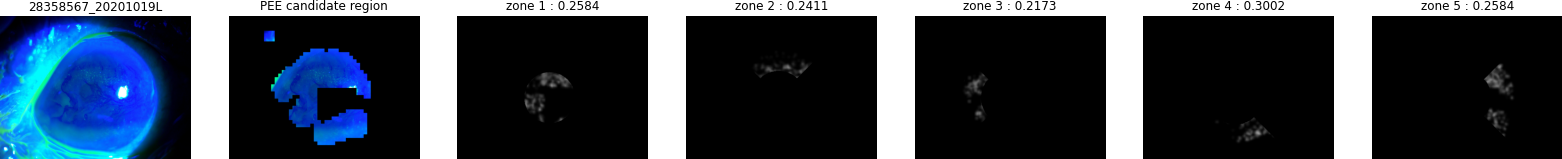

Supplement: S2 Dataset — (ZIP) [file pone.0299776.s003.zip › 28358567_20201019L/28358567_20201019L_zone.png]

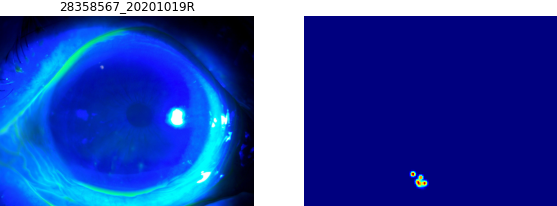

Supplement: S2 Dataset — (ZIP) [file pone.0299776.s003.zip › 28358567_20201019R/28358567_20201019R_densitymap.png]

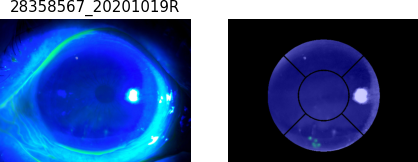

Supplement: S2 Dataset — (ZIP) [file pone.0299776.s003.zip › 28358567_20201019R/28358567_20201019R_whole.png]

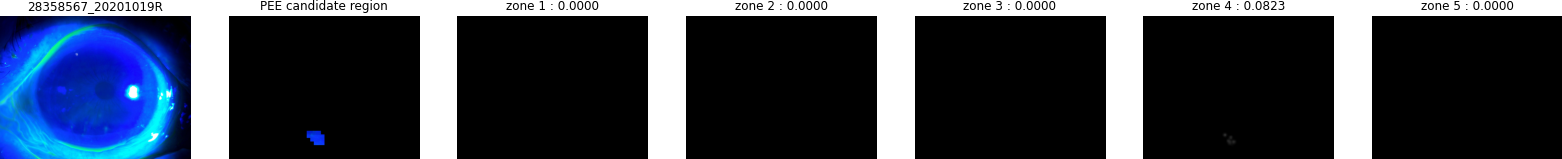

Supplement: S2 Dataset — (ZIP) [file pone.0299776.s003.zip › 28358567_20201019R/28358567_20201019R_zone.png]

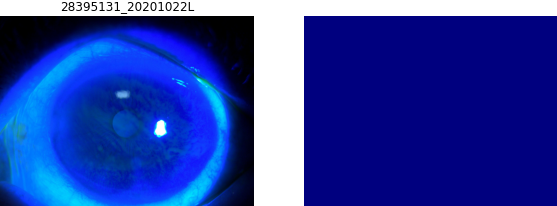

Supplement: S2 Dataset — (ZIP) [file pone.0299776.s003.zip › 28395131_20201022L/28395131_20201022L_densitymap.png]

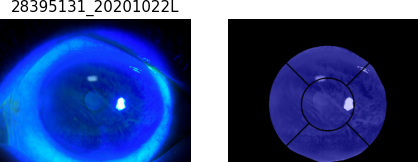

Supplement: S2 Dataset — (ZIP) [file pone.0299776.s003.zip › 28395131_20201022L/28395131_20201022L_whole.png]

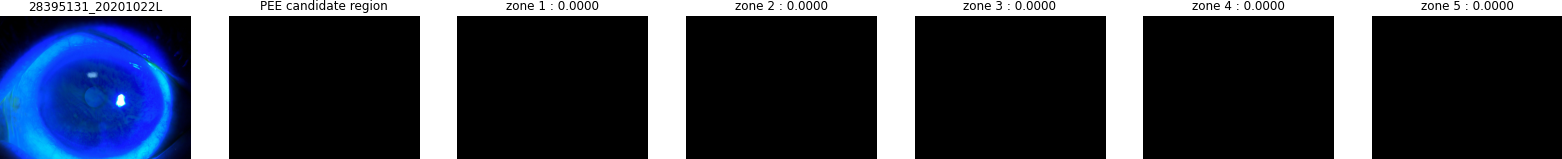

Supplement: S2 Dataset — (ZIP) [file pone.0299776.s003.zip › 28395131_20201022L/28395131_20201022L_zone.png]

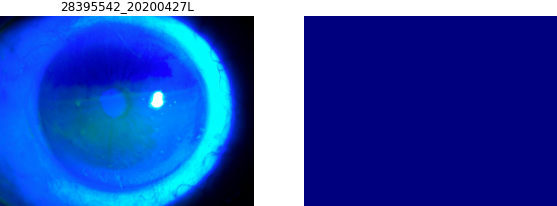

Supplement: S2 Dataset — (ZIP) [file pone.0299776.s003.zip › 28395542_20200427L/28395542_20200427L_densitymap.png]

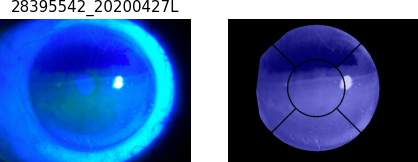

Supplement: S2 Dataset — (ZIP) [file pone.0299776.s003.zip › 28395542_20200427L/28395542_20200427L_whole.png]

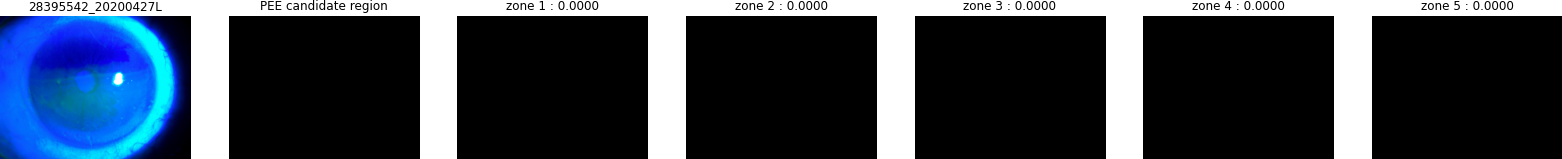

Supplement: S2 Dataset — (ZIP) [file pone.0299776.s003.zip › 28395542_20200427L/28395542_20200427L_zone.png]

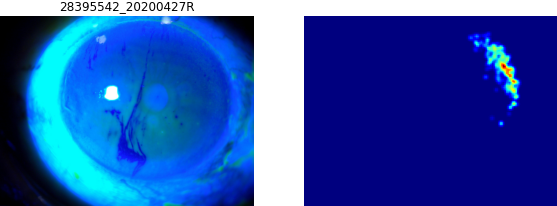

Supplement: S2 Dataset — (ZIP) [file pone.0299776.s003.zip › 28395542_20200427R/28395542_20200427R_densitymap.png]

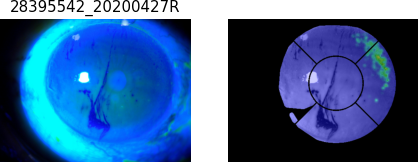

Supplement: S2 Dataset — (ZIP) [file pone.0299776.s003.zip › 28395542_20200427R/28395542_20200427R_whole.png]

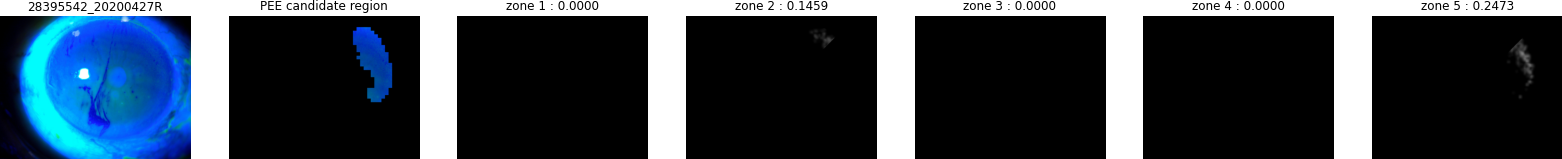

Supplement: S2 Dataset — (ZIP) [file pone.0299776.s003.zip › 28395542_20200427R/28395542_20200427R_zone.png]

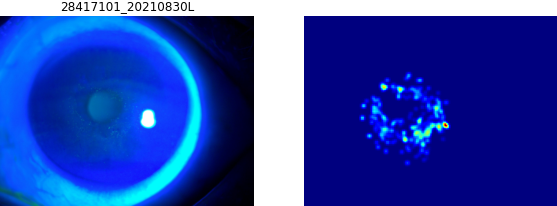

Supplement: S2 Dataset — (ZIP) [file pone.0299776.s003.zip › 28417101_20210830L/28417101_20210830L_densitymap.png]

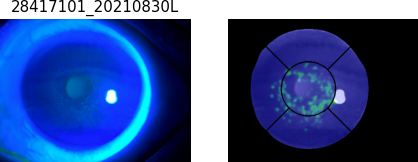

Supplement: S2 Dataset — (ZIP) [file pone.0299776.s003.zip › 28417101_20210830L/28417101_20210830L_whole.png]

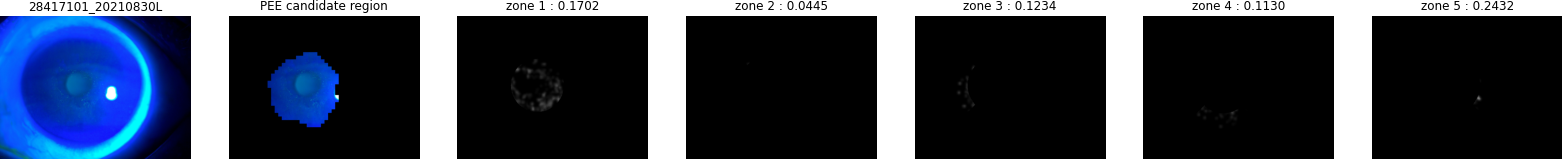

Supplement: S2 Dataset — (ZIP) [file pone.0299776.s003.zip › 28417101_20210830L/28417101_20210830L_zone.png]

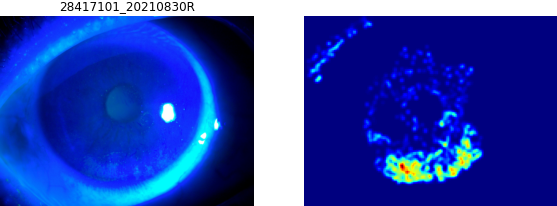

Supplement: S2 Dataset — (ZIP) [file pone.0299776.s003.zip › 28417101_20210830R/28417101_20210830R_densitymap.png]

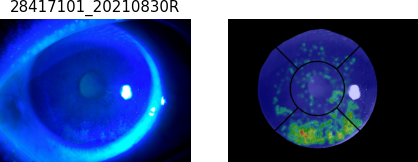

Supplement: S2 Dataset — (ZIP) [file pone.0299776.s003.zip › 28417101_20210830R/28417101_20210830R_whole.png]

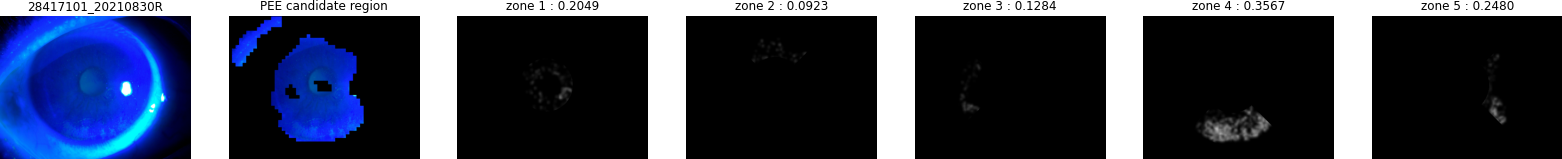

Supplement: S2 Dataset — (ZIP) [file pone.0299776.s003.zip › 28417101_20210830R/28417101_20210830R_zone.png]

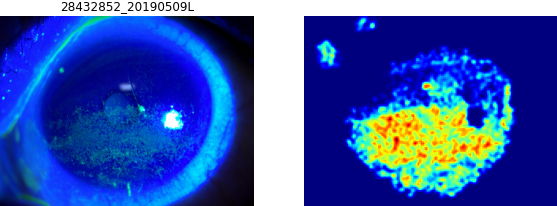

Supplement: S2 Dataset — (ZIP) [file pone.0299776.s003.zip › 28432852_20190509L/28432852_20190509L_densitymap.png]

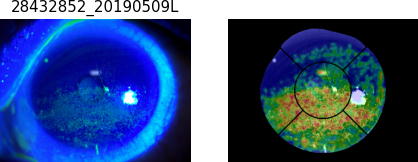

Supplement: S2 Dataset — (ZIP) [file pone.0299776.s003.zip › 28432852_20190509L/28432852_20190509L_whole.png]

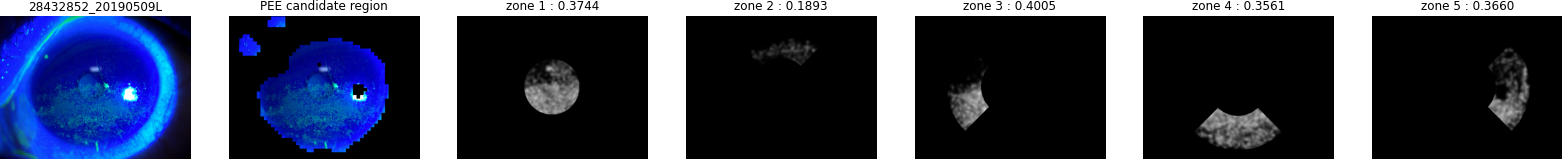

Supplement: S2 Dataset — (ZIP) [file pone.0299776.s003.zip › 28432852_20190509L/28432852_20190509L_zone.png]

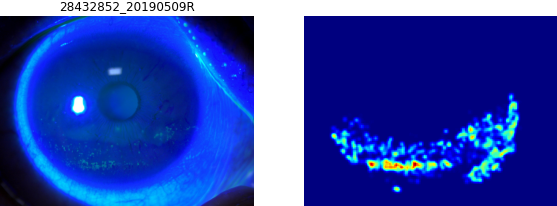

Supplement: S2 Dataset — (ZIP) [file pone.0299776.s003.zip › 28432852_20190509R/28432852_20190509R_densitymap.png]

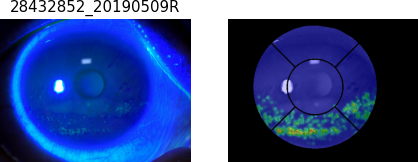

Supplement: S2 Dataset — (ZIP) [file pone.0299776.s003.zip › 28432852_20190509R/28432852_20190509R_whole.png]

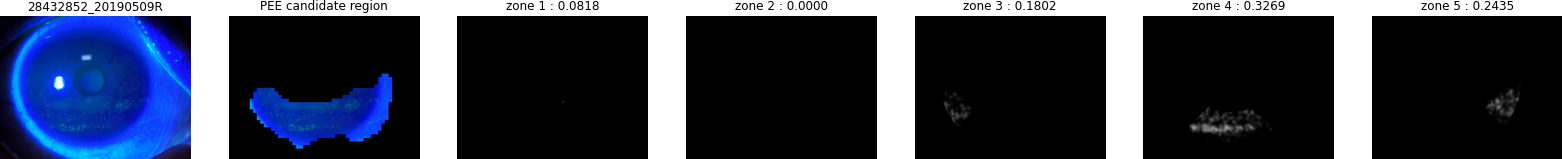

Supplement: S2 Dataset — (ZIP) [file pone.0299776.s003.zip › 28432852_20190509R/28432852_20190509R_zone.png]

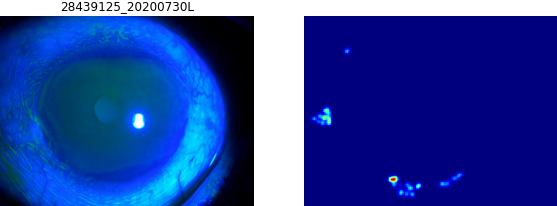

Supplement: S2 Dataset — (ZIP) [file pone.0299776.s003.zip › 28439125_20200730L/28439125_20200730L_densitymap.png]

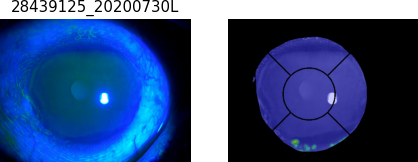

Supplement: S2 Dataset — (ZIP) [file pone.0299776.s003.zip › 28439125_20200730L/28439125_20200730L_whole.png]

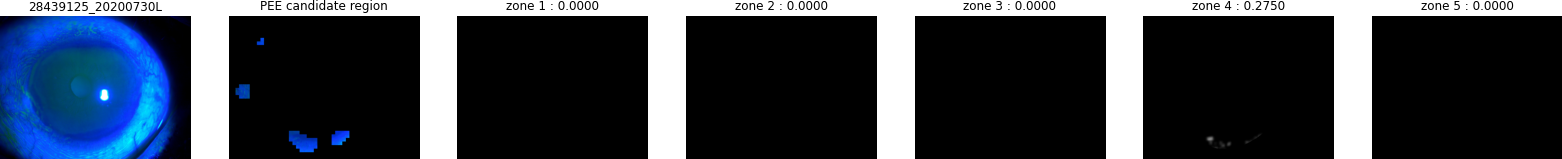

Supplement: S2 Dataset — (ZIP) [file pone.0299776.s003.zip › 28439125_20200730L/28439125_20200730L_zone.png]

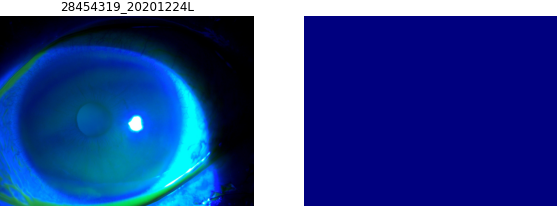

Supplement: S2 Dataset — (ZIP) [file pone.0299776.s003.zip › 28454319_20201224L/28454319_20201224L_densitymap.png]

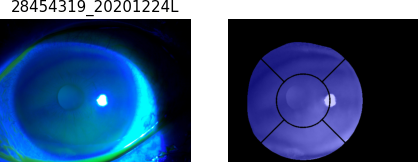

Supplement: S2 Dataset — (ZIP) [file pone.0299776.s003.zip › 28454319_20201224L/28454319_20201224L_whole.png]

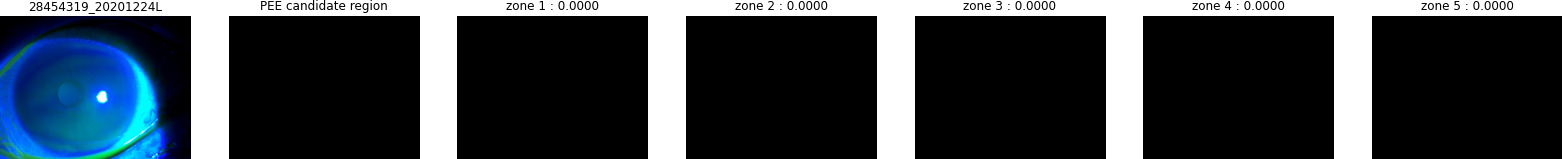

Supplement: S2 Dataset — (ZIP) [file pone.0299776.s003.zip › 28454319_20201224L/28454319_20201224L_zone.png]

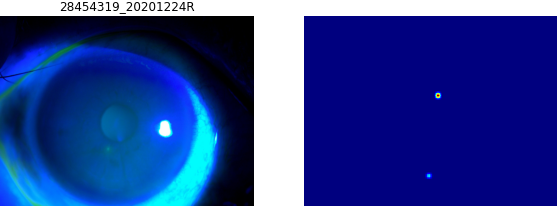

Supplement: S2 Dataset — (ZIP) [file pone.0299776.s003.zip › 28454319_20201224R/28454319_20201224R_densitymap.png]

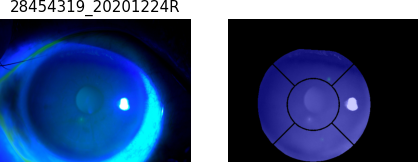

Supplement: S2 Dataset — (ZIP) [file pone.0299776.s003.zip › 28454319_20201224R/28454319_20201224R_whole.png]

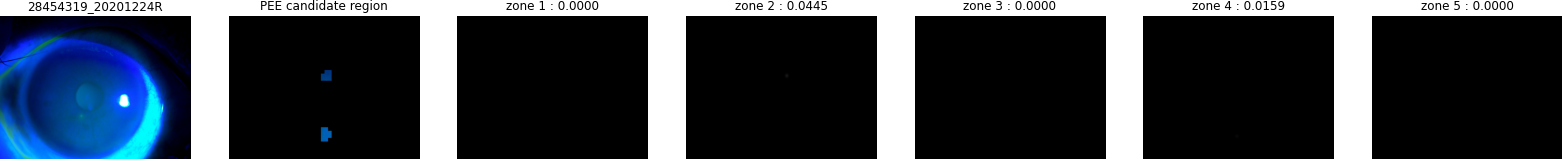

Supplement: S2 Dataset — (ZIP) [file pone.0299776.s003.zip › 28454319_20201224R/28454319_20201224R_zone.png]

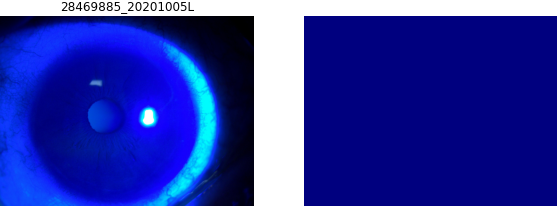

Supplement: S2 Dataset — (ZIP) [file pone.0299776.s003.zip › 28469885_20201005L/28469885_20201005L_densitymap.png]

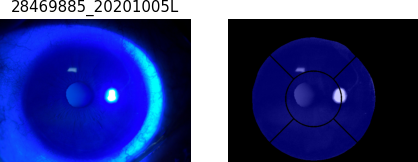

Supplement: S2 Dataset — (ZIP) [file pone.0299776.s003.zip › 28469885_20201005L/28469885_20201005L_whole.png]

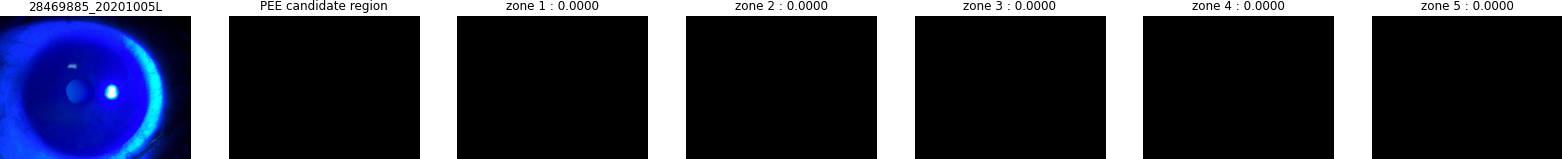

Supplement: S2 Dataset — (ZIP) [file pone.0299776.s003.zip › 28469885_20201005L/28469885_20201005L_zone.png]

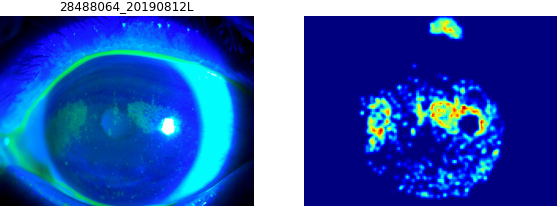

Supplement: S2 Dataset — (ZIP) [file pone.0299776.s003.zip › 28488064_20190812L/28488064_20190812L_densitymap.png]

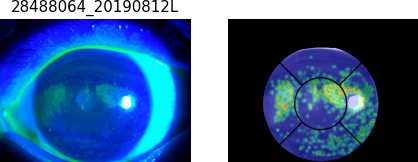

Supplement: S2 Dataset — (ZIP) [file pone.0299776.s003.zip › 28488064_20190812L/28488064_20190812L_whole.png]

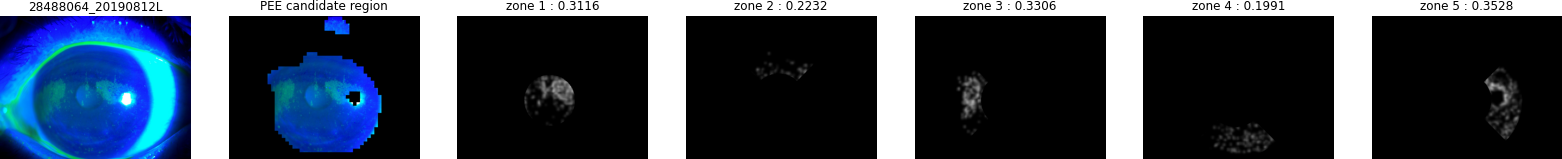

Supplement: S2 Dataset — (ZIP) [file pone.0299776.s003.zip › 28488064_20190812L/28488064_20190812L_zone.png]

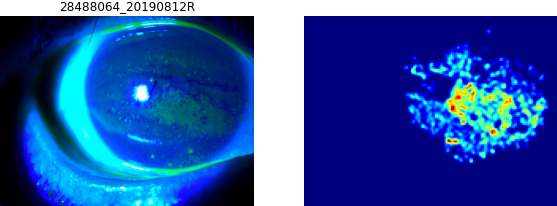

Supplement: S2 Dataset — (ZIP) [file pone.0299776.s003.zip › 28488064_20190812R/28488064_20190812R_densitymap.png]

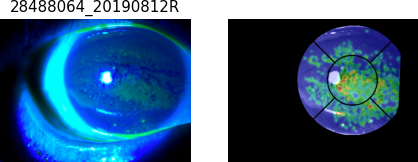

Supplement: S2 Dataset — (ZIP) [file pone.0299776.s003.zip › 28488064_20190812R/28488064_20190812R_whole.png]

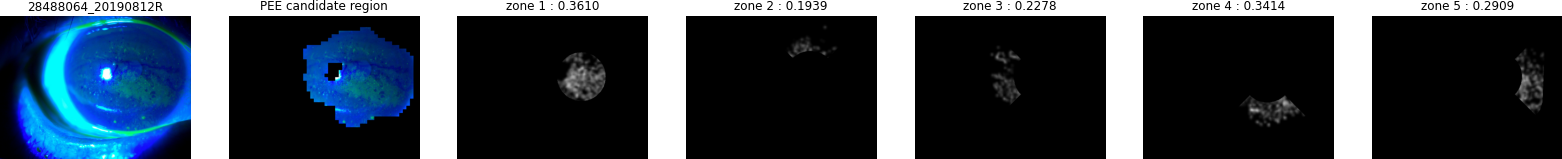

Supplement: S2 Dataset — (ZIP) [file pone.0299776.s003.zip › 28488064_20190812R/28488064_20190812R_zone.png]

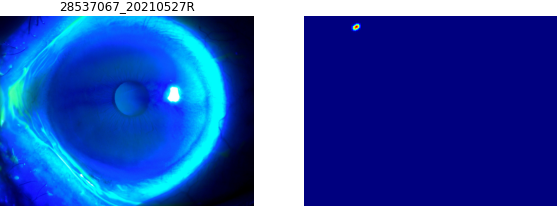

Supplement: S2 Dataset — (ZIP) [file pone.0299776.s003.zip › 28537067_20210527R/28537067_20210527R_densitymap.png]

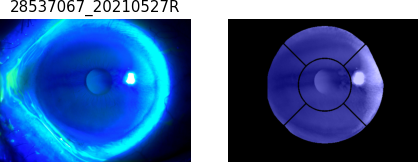

Supplement: S2 Dataset — (ZIP) [file pone.0299776.s003.zip › 28537067_20210527R/28537067_20210527R_whole.png]

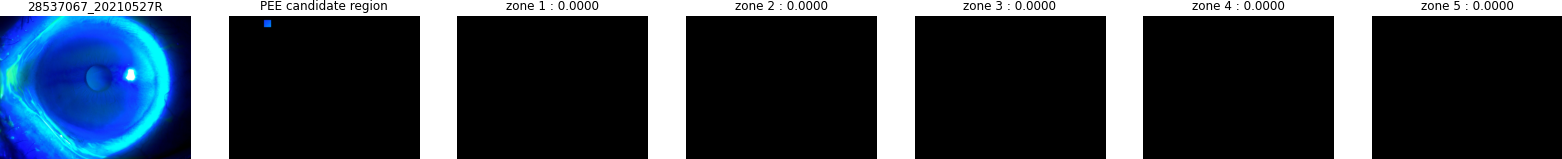

Supplement: S2 Dataset — (ZIP) [file pone.0299776.s003.zip › 28537067_20210527R/28537067_20210527R_zone.png]

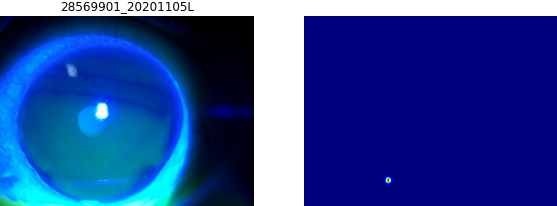

Supplement: S2 Dataset — (ZIP) [file pone.0299776.s003.zip › 28569901_20201105L/28569901_20201105L_densitymap.png]

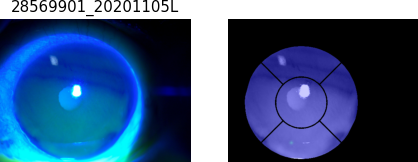

Supplement: S2 Dataset — (ZIP) [file pone.0299776.s003.zip › 28569901_20201105L/28569901_20201105L_whole.png]

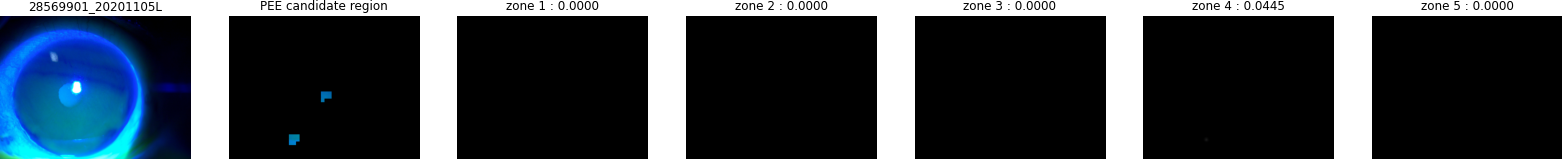

Supplement: S2 Dataset — (ZIP) [file pone.0299776.s003.zip › 28569901_20201105L/28569901_20201105L_zone.png]

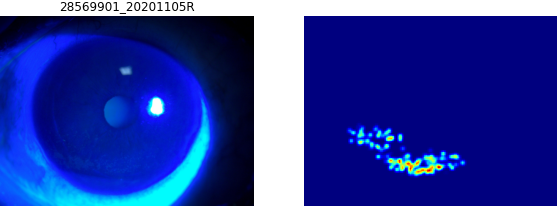

Supplement: S2 Dataset — (ZIP) [file pone.0299776.s003.zip › 28569901_20201105R/28569901_20201105R_densitymap.png]

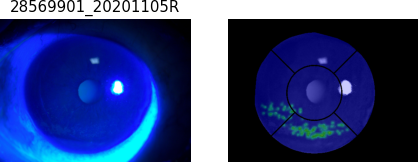

Supplement: S2 Dataset — (ZIP) [file pone.0299776.s003.zip › 28569901_20201105R/28569901_20201105R_whole.png]

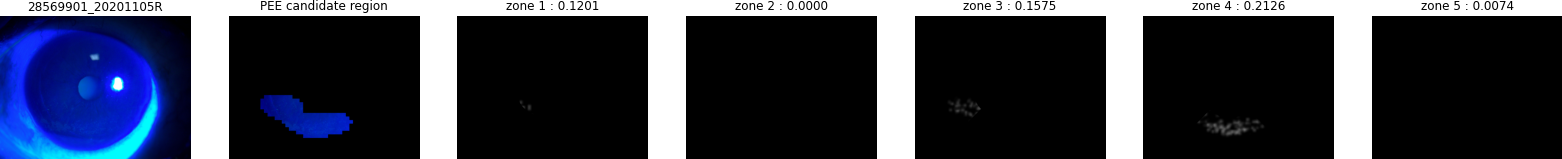

Supplement: S2 Dataset — (ZIP) [file pone.0299776.s003.zip › 28569901_20201105R/28569901_20201105R_zone.png]

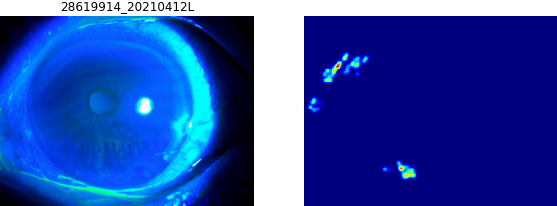

Supplement: S2 Dataset — (ZIP) [file pone.0299776.s003.zip › 28619914_20210412L/28619914_20210412L_densitymap.png]

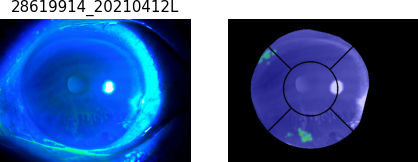

Supplement: S2 Dataset — (ZIP) [file pone.0299776.s003.zip › 28619914_20210412L/28619914_20210412L_whole.png]

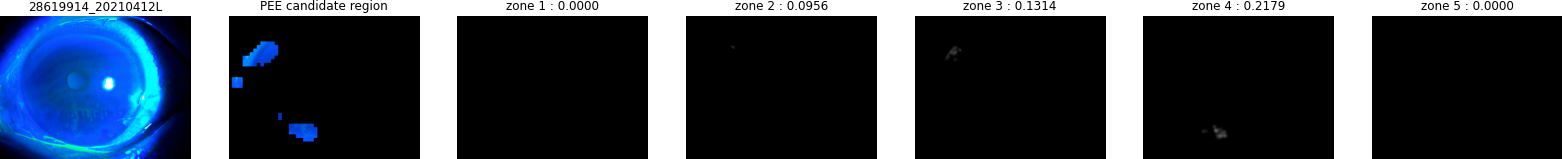

Supplement: S2 Dataset — (ZIP) [file pone.0299776.s003.zip › 28619914_20210412L/28619914_20210412L_zone.png]

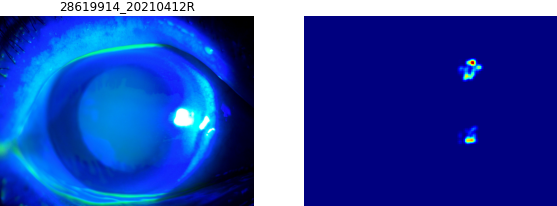

Supplement: S2 Dataset — (ZIP) [file pone.0299776.s003.zip › 28619914_20210412R/28619914_20210412R_densitymap.png]

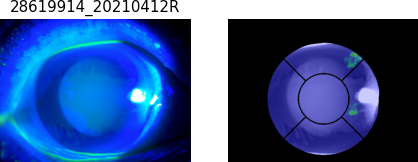

Supplement: S2 Dataset — (ZIP) [file pone.0299776.s003.zip › 28619914_20210412R/28619914_20210412R_whole.png]

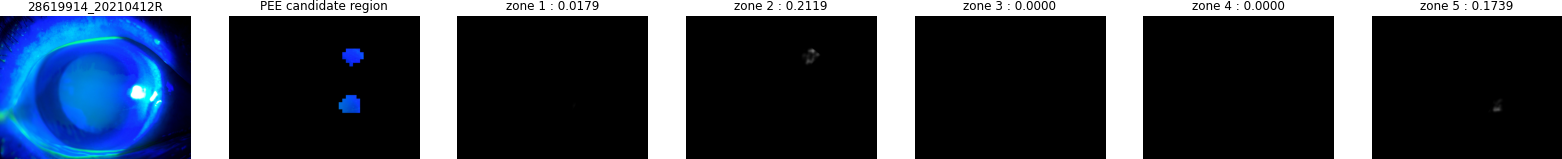

Supplement: S2 Dataset — (ZIP) [file pone.0299776.s003.zip › 28619914_20210412R/28619914_20210412R_zone.png]

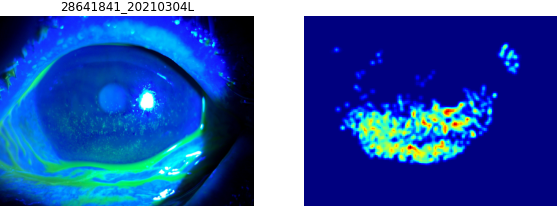

Supplement: S2 Dataset — (ZIP) [file pone.0299776.s003.zip › 28641841_20210304L/28641841_20210304L_densitymap.png]

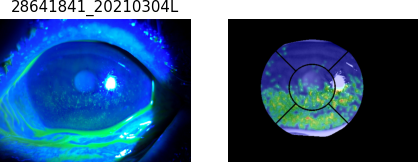

Supplement: S2 Dataset — (ZIP) [file pone.0299776.s003.zip › 28641841_20210304L/28641841_20210304L_whole.png]

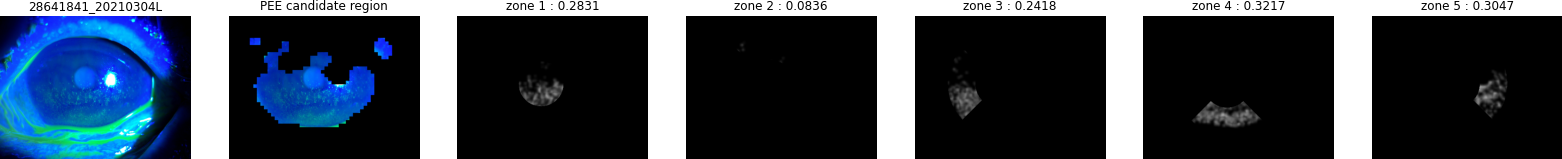

Supplement: S2 Dataset — (ZIP) [file pone.0299776.s003.zip › 28641841_20210304L/28641841_20210304L_zone.png]

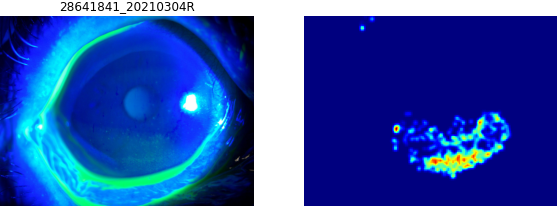

Supplement: S2 Dataset — (ZIP) [file pone.0299776.s003.zip › 28641841_20210304R/28641841_20210304R_densitymap.png]

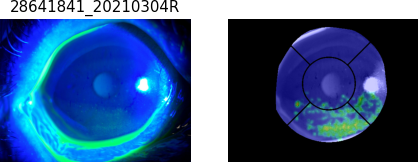

Supplement: S2 Dataset — (ZIP) [file pone.0299776.s003.zip › 28641841_20210304R/28641841_20210304R_whole.png]

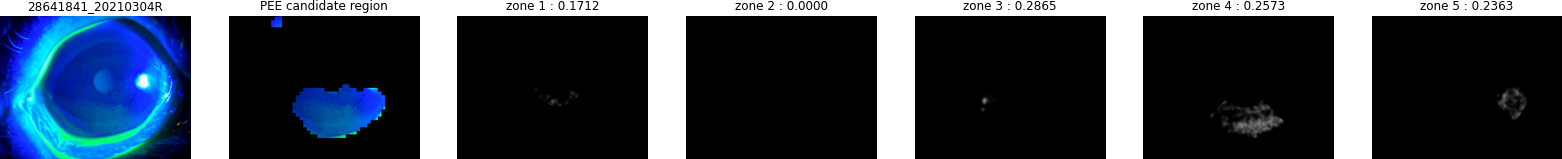

Supplement: S2 Dataset — (ZIP) [file pone.0299776.s003.zip › 28641841_20210304R/28641841_20210304R_zone.png]

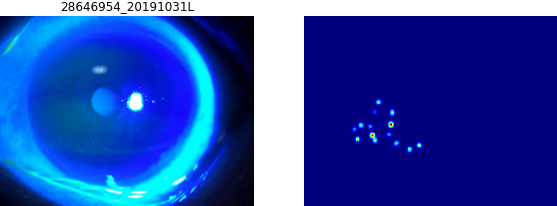

Supplement: S2 Dataset — (ZIP) [file pone.0299776.s003.zip › 28646954_20191031L/28646954_20191031L_densitymap.png]

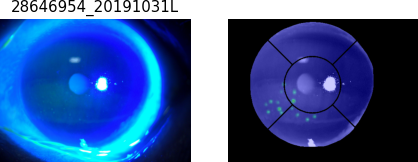

Supplement: S2 Dataset — (ZIP) [file pone.0299776.s003.zip › 28646954_20191031L/28646954_20191031L_whole.png]

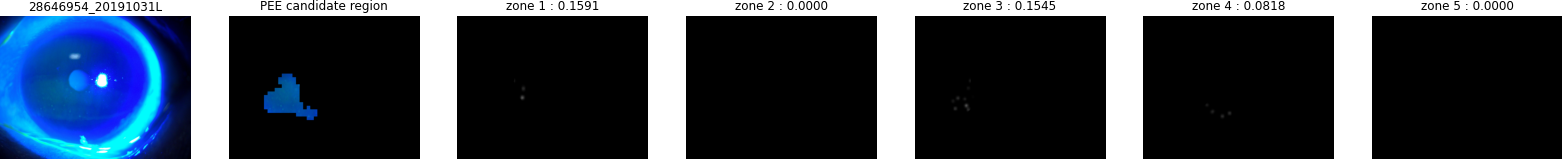

Supplement: S2 Dataset — (ZIP) [file pone.0299776.s003.zip › 28646954_20191031L/28646954_20191031L_zone.png]

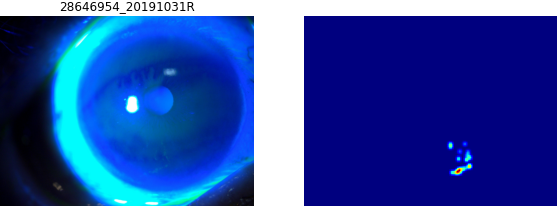

Supplement: S2 Dataset — (ZIP) [file pone.0299776.s003.zip › 28646954_20191031R/28646954_20191031R_densitymap.png]
