# Supplementary figures and images for: Deep learning-based fully automated grading system for dry eye disease severity (part 6 of 6)
Source: PLoS One. 2024 Mar 14;19(3):e0299776. doi: 10.1371/journal.pone.0299776 (PMC10939279; doi:10.1371/journal.pone.0299776)

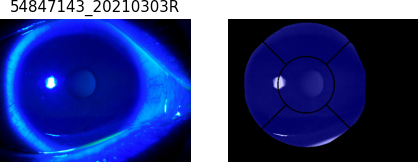

Supplement: S3 Dataset — (ZIP) [file pone.0299776.s004.zip › 54847143_20210303R/54847143_20210303R_whole.png]

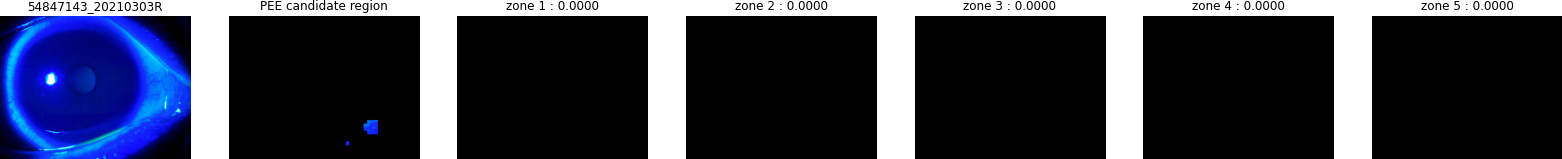

Supplement: S3 Dataset — (ZIP) [file pone.0299776.s004.zip › 54847143_20210303R/54847143_20210303R_zone.png]

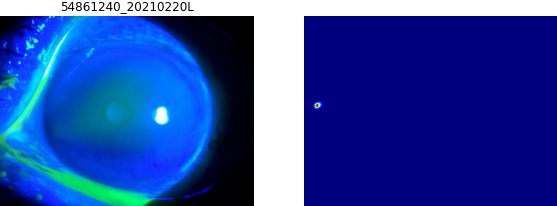

Supplement: S3 Dataset — (ZIP) [file pone.0299776.s004.zip › 54861240_20210220L/54861240_20210220L_densitymap.png]

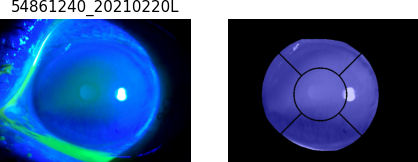

Supplement: S3 Dataset — (ZIP) [file pone.0299776.s004.zip › 54861240_20210220L/54861240_20210220L_whole.png]

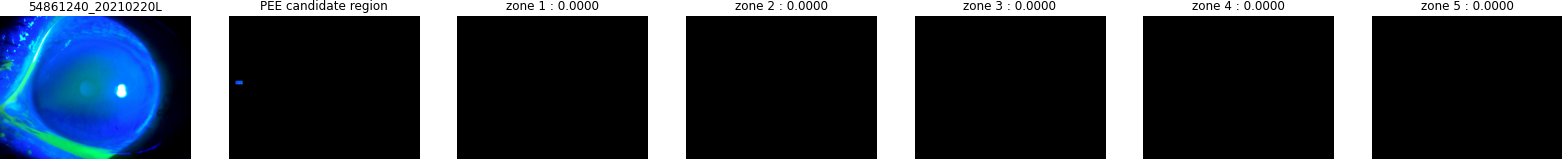

Supplement: S3 Dataset — (ZIP) [file pone.0299776.s004.zip › 54861240_20210220L/54861240_20210220L_zone.png]

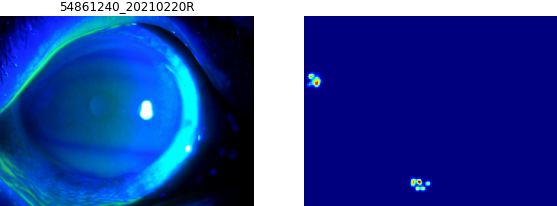

Supplement: S3 Dataset — (ZIP) [file pone.0299776.s004.zip › 54861240_20210220R/54861240_20210220R_densitymap.png]

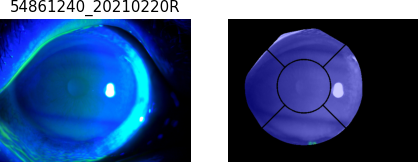

Supplement: S3 Dataset — (ZIP) [file pone.0299776.s004.zip › 54861240_20210220R/54861240_20210220R_whole.png]

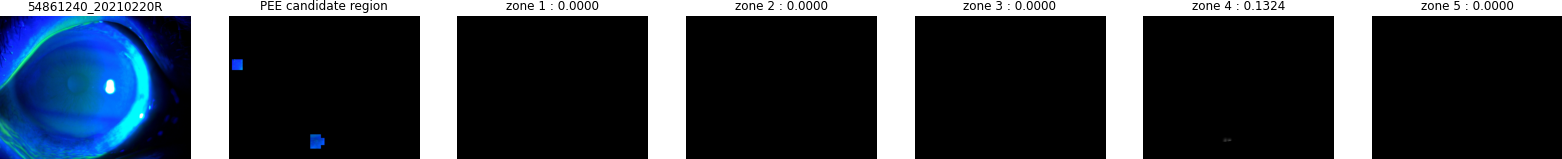

Supplement: S3 Dataset — (ZIP) [file pone.0299776.s004.zip › 54861240_20210220R/54861240_20210220R_zone.png]

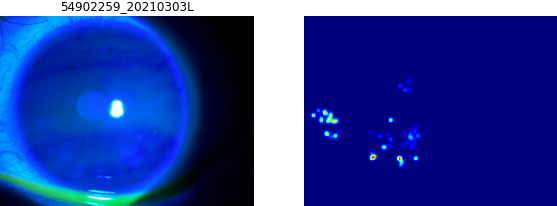

Supplement: S3 Dataset — (ZIP) [file pone.0299776.s004.zip › 54902259_20210303L/54902259_20210303L_densitymap.png]

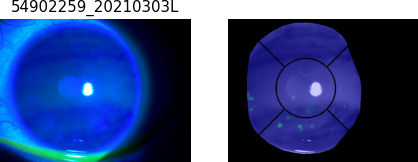

Supplement: S3 Dataset — (ZIP) [file pone.0299776.s004.zip › 54902259_20210303L/54902259_20210303L_whole.png]

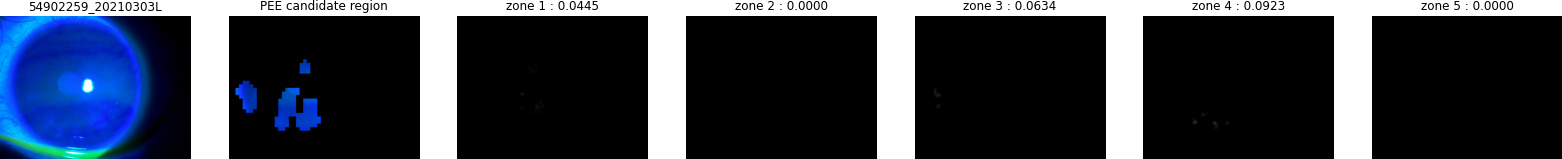

Supplement: S3 Dataset — (ZIP) [file pone.0299776.s004.zip › 54902259_20210303L/54902259_20210303L_zone.png]

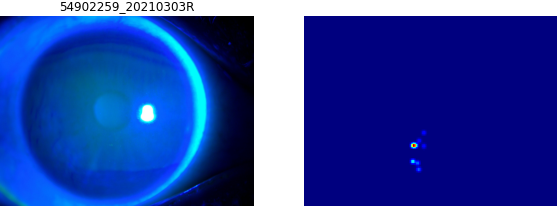

Supplement: S3 Dataset — (ZIP) [file pone.0299776.s004.zip › 54902259_20210303R/54902259_20210303R_densitymap.png]

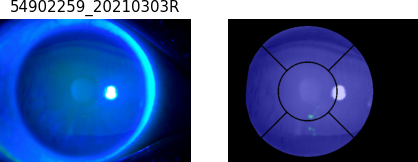

Supplement: S3 Dataset — (ZIP) [file pone.0299776.s004.zip › 54902259_20210303R/54902259_20210303R_whole.png]

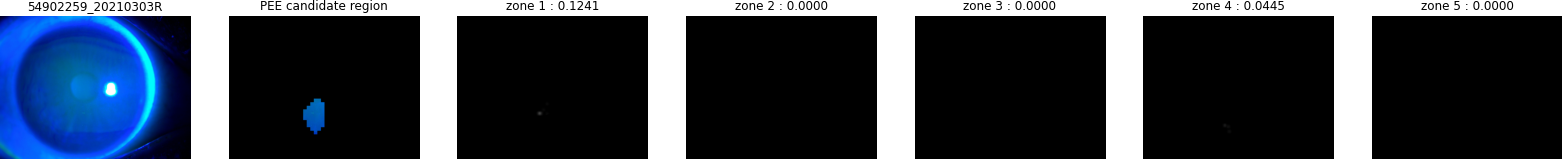

Supplement: S3 Dataset — (ZIP) [file pone.0299776.s004.zip › 54902259_20210303R/54902259_20210303R_zone.png]

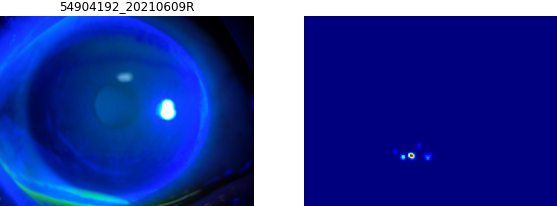

Supplement: S3 Dataset — (ZIP) [file pone.0299776.s004.zip › 54904192_20210609R/54904192_20210609R_densitymap.png]

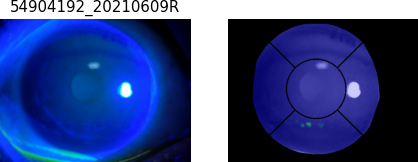

Supplement: S3 Dataset — (ZIP) [file pone.0299776.s004.zip › 54904192_20210609R/54904192_20210609R_whole.png]

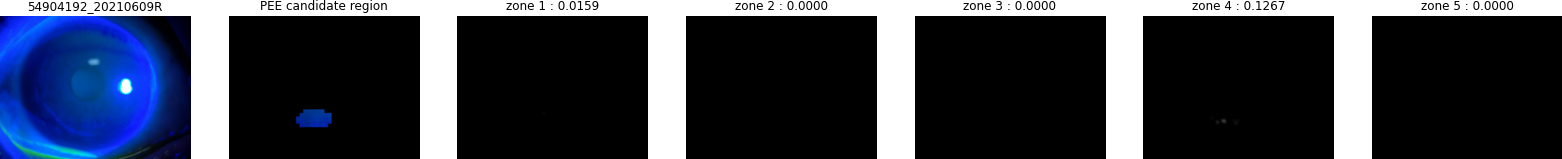

Supplement: S3 Dataset — (ZIP) [file pone.0299776.s004.zip › 54904192_20210609R/54904192_20210609R_zone.png]

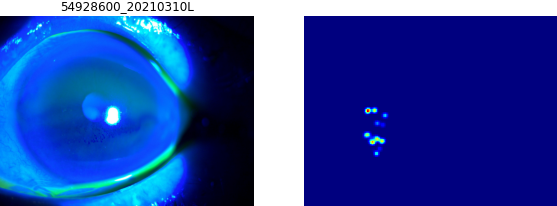

Supplement: S3 Dataset — (ZIP) [file pone.0299776.s004.zip › 54928600_20210310L/54928600_20210310L_densitymap.png]

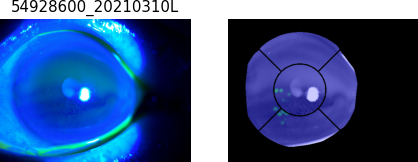

Supplement: S3 Dataset — (ZIP) [file pone.0299776.s004.zip › 54928600_20210310L/54928600_20210310L_whole.png]

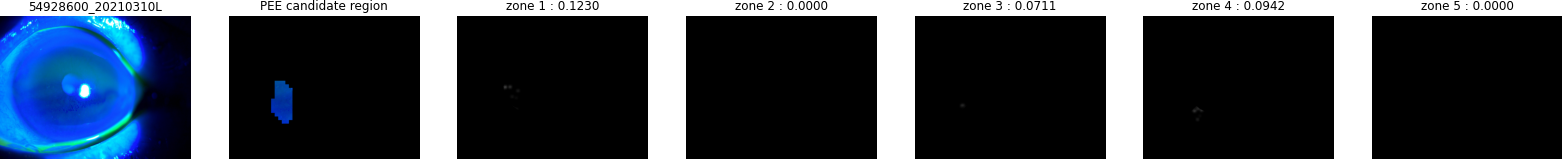

Supplement: S3 Dataset — (ZIP) [file pone.0299776.s004.zip › 54928600_20210310L/54928600_20210310L_zone.png]

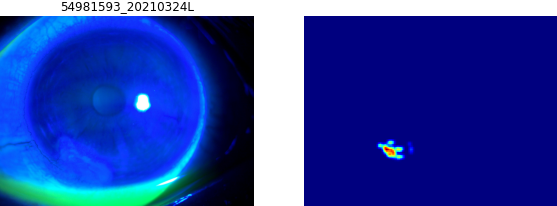

Supplement: S3 Dataset — (ZIP) [file pone.0299776.s004.zip › 54981593_20210324L/54981593_20210324L_densitymap.png]

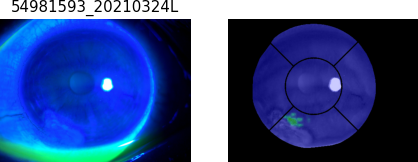

Supplement: S3 Dataset — (ZIP) [file pone.0299776.s004.zip › 54981593_20210324L/54981593_20210324L_whole.png]

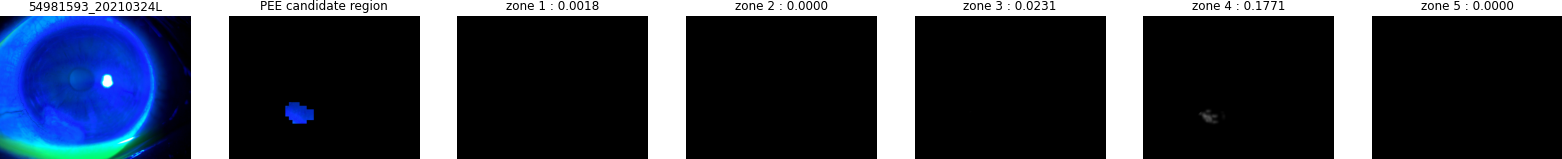

Supplement: S3 Dataset — (ZIP) [file pone.0299776.s004.zip › 54981593_20210324L/54981593_20210324L_zone.png]

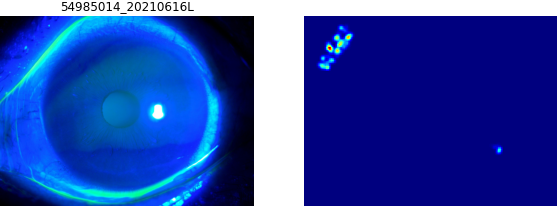

Supplement: S3 Dataset — (ZIP) [file pone.0299776.s004.zip › 54985014_20210616L/54985014_20210616L_densitymap.png]

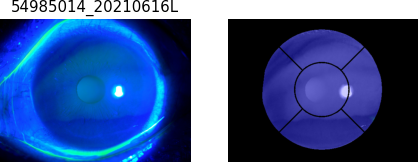

Supplement: S3 Dataset — (ZIP) [file pone.0299776.s004.zip › 54985014_20210616L/54985014_20210616L_whole.png]

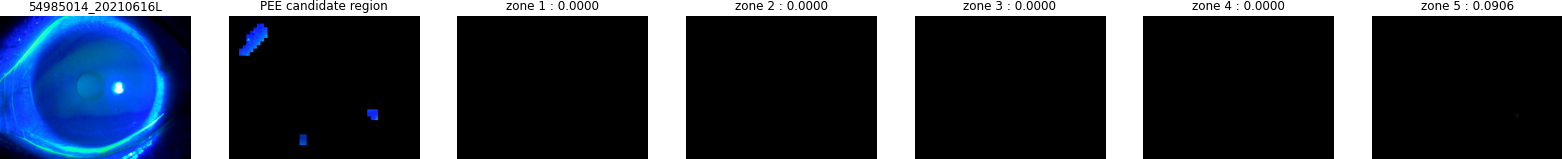

Supplement: S3 Dataset — (ZIP) [file pone.0299776.s004.zip › 54985014_20210616L/54985014_20210616L_zone.png]

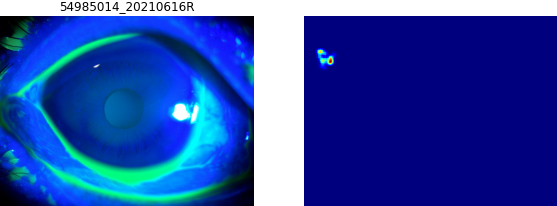

Supplement: S3 Dataset — (ZIP) [file pone.0299776.s004.zip › 54985014_20210616R/54985014_20210616R_densitymap.png]

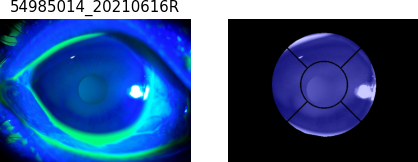

Supplement: S3 Dataset — (ZIP) [file pone.0299776.s004.zip › 54985014_20210616R/54985014_20210616R_whole.png]

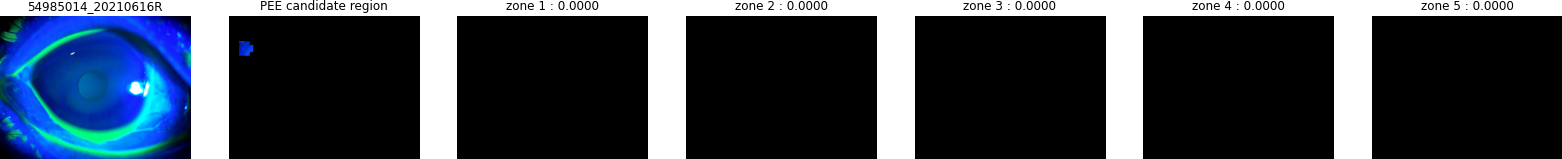

Supplement: S3 Dataset — (ZIP) [file pone.0299776.s004.zip › 54985014_20210616R/54985014_20210616R_zone.png]

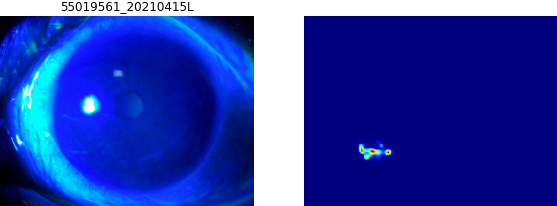

Supplement: S3 Dataset — (ZIP) [file pone.0299776.s004.zip › 55019561_20210415L/55019561_20210415L_densitymap.png]

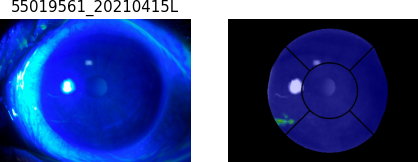

Supplement: S3 Dataset — (ZIP) [file pone.0299776.s004.zip › 55019561_20210415L/55019561_20210415L_whole.png]

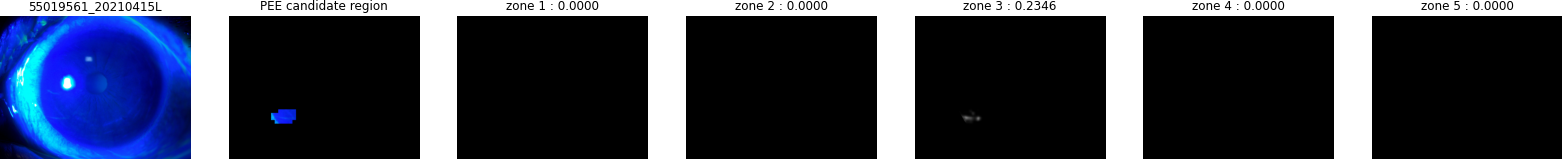

Supplement: S3 Dataset — (ZIP) [file pone.0299776.s004.zip › 55019561_20210415L/55019561_20210415L_zone.png]

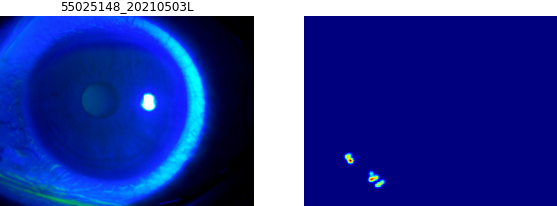

Supplement: S3 Dataset — (ZIP) [file pone.0299776.s004.zip › 55025148_20210503L/55025148_20210503L_densitymap.png]

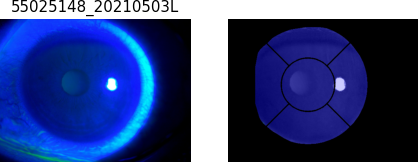

Supplement: S3 Dataset — (ZIP) [file pone.0299776.s004.zip › 55025148_20210503L/55025148_20210503L_whole.png]

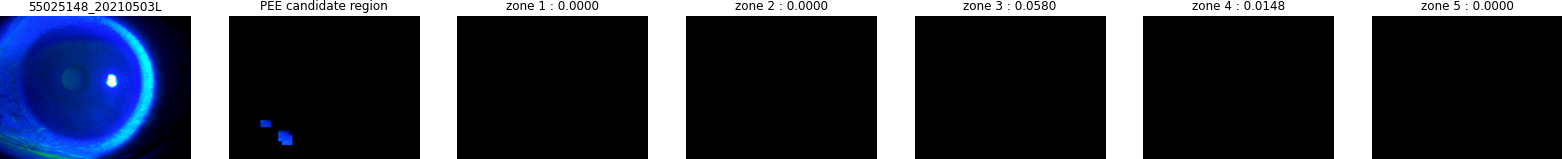

Supplement: S3 Dataset — (ZIP) [file pone.0299776.s004.zip › 55025148_20210503L/55025148_20210503L_zone.png]

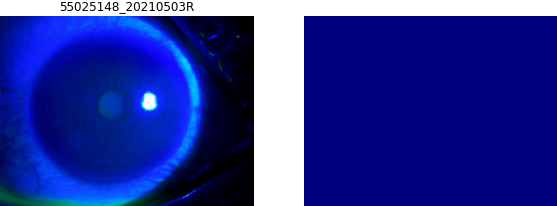

Supplement: S3 Dataset — (ZIP) [file pone.0299776.s004.zip › 55025148_20210503R/55025148_20210503R_densitymap.png]

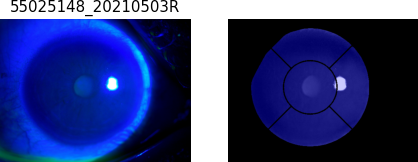

Supplement: S3 Dataset — (ZIP) [file pone.0299776.s004.zip › 55025148_20210503R/55025148_20210503R_whole.png]

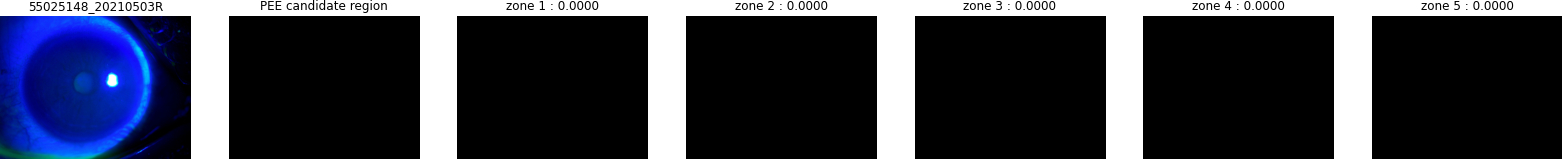

Supplement: S3 Dataset — (ZIP) [file pone.0299776.s004.zip › 55025148_20210503R/55025148_20210503R_zone.png]

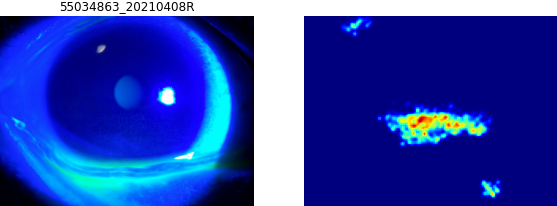

Supplement: S3 Dataset — (ZIP) [file pone.0299776.s004.zip › 55034863_20210408R/55034863_20210408R_densitymap.png]

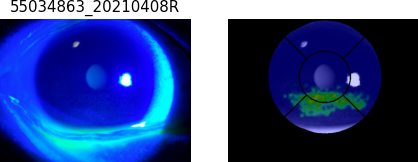

Supplement: S3 Dataset — (ZIP) [file pone.0299776.s004.zip › 55034863_20210408R/55034863_20210408R_whole.png]

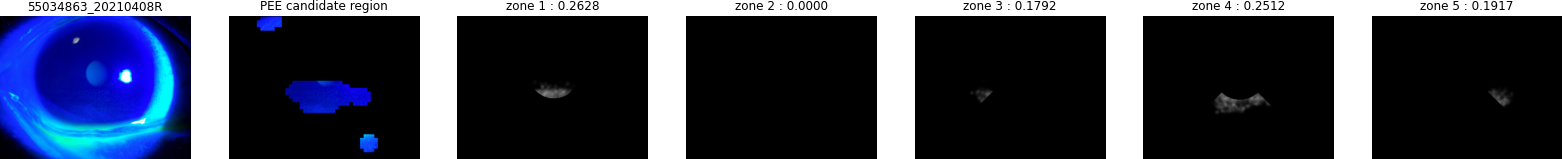

Supplement: S3 Dataset — (ZIP) [file pone.0299776.s004.zip › 55034863_20210408R/55034863_20210408R_zone.png]

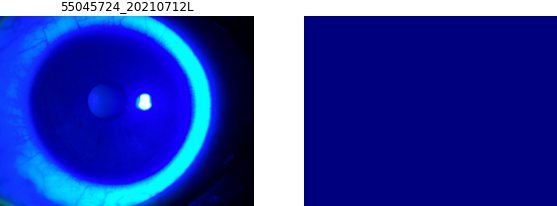

Supplement: S3 Dataset — (ZIP) [file pone.0299776.s004.zip › 55045724_20210712L/55045724_20210712L_densitymap.png]

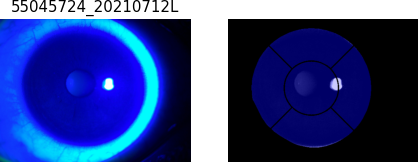

Supplement: S3 Dataset — (ZIP) [file pone.0299776.s004.zip › 55045724_20210712L/55045724_20210712L_whole.png]

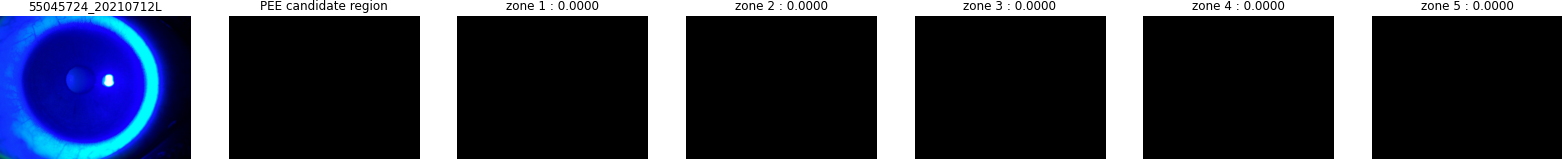

Supplement: S3 Dataset — (ZIP) [file pone.0299776.s004.zip › 55045724_20210712L/55045724_20210712L_zone.png]

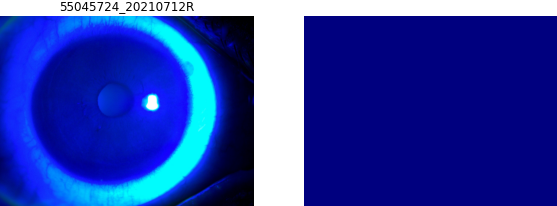

Supplement: S3 Dataset — (ZIP) [file pone.0299776.s004.zip › 55045724_20210712R/55045724_20210712R_densitymap.png]

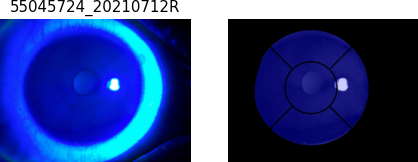

Supplement: S3 Dataset — (ZIP) [file pone.0299776.s004.zip › 55045724_20210712R/55045724_20210712R_whole.png]

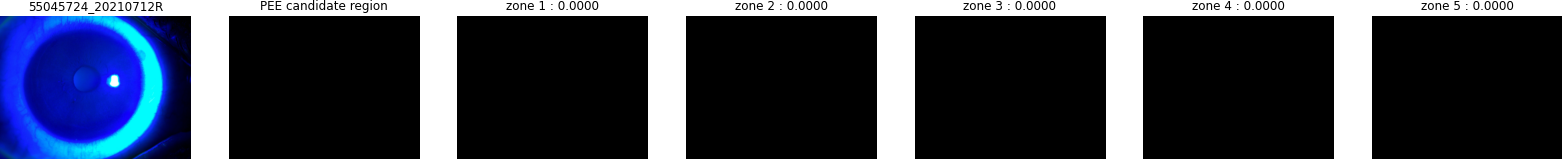

Supplement: S3 Dataset — (ZIP) [file pone.0299776.s004.zip › 55045724_20210712R/55045724_20210712R_zone.png]

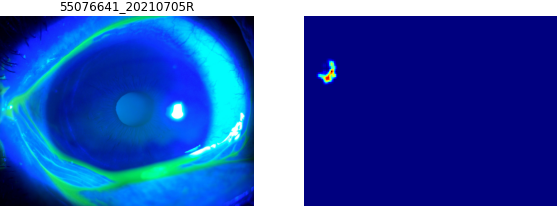

Supplement: S3 Dataset — (ZIP) [file pone.0299776.s004.zip › 55076641_20210705R/55076641_20210705R_densitymap.png]

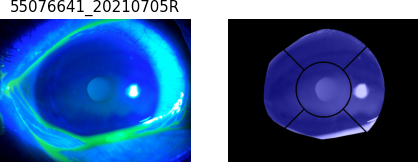

Supplement: S3 Dataset — (ZIP) [file pone.0299776.s004.zip › 55076641_20210705R/55076641_20210705R_whole.png]

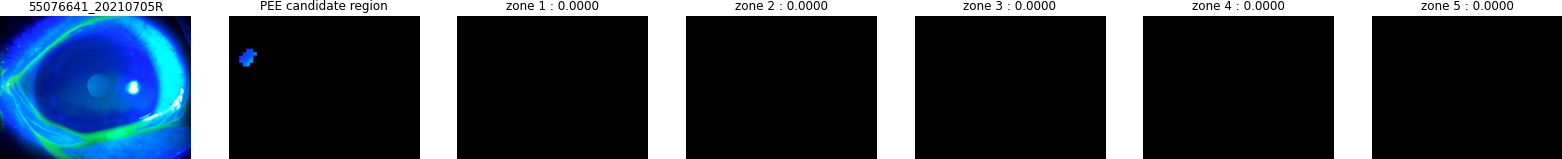

Supplement: S3 Dataset — (ZIP) [file pone.0299776.s004.zip › 55076641_20210705R/55076641_20210705R_zone.png]

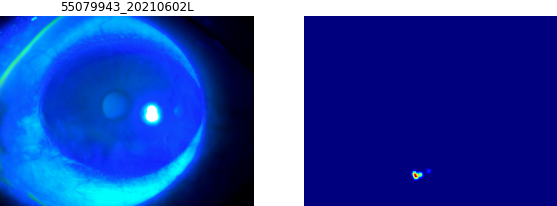

Supplement: S3 Dataset — (ZIP) [file pone.0299776.s004.zip › 55079943_20210602L/55079943_20210602L_densitymap.png]

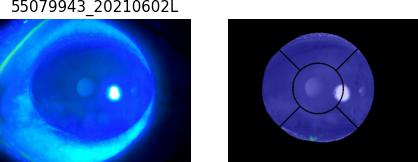

Supplement: S3 Dataset — (ZIP) [file pone.0299776.s004.zip › 55079943_20210602L/55079943_20210602L_whole.png]

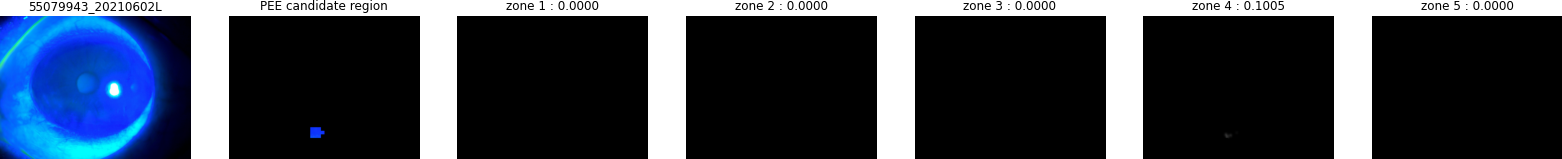

Supplement: S3 Dataset — (ZIP) [file pone.0299776.s004.zip › 55079943_20210602L/55079943_20210602L_zone.png]

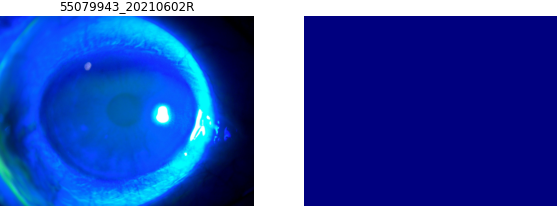

Supplement: S3 Dataset — (ZIP) [file pone.0299776.s004.zip › 55079943_20210602R/55079943_20210602R_densitymap.png]

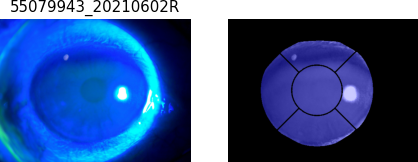

Supplement: S3 Dataset — (ZIP) [file pone.0299776.s004.zip › 55079943_20210602R/55079943_20210602R_whole.png]

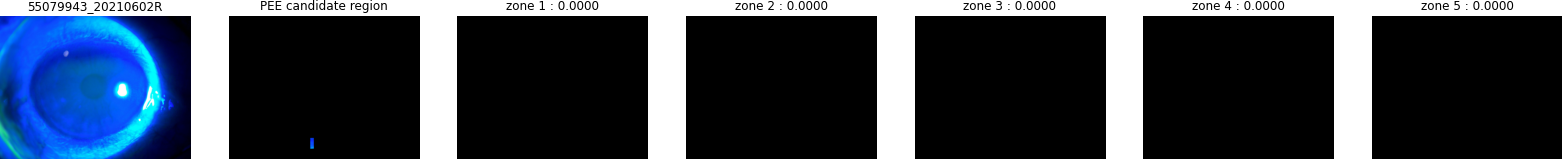

Supplement: S3 Dataset — (ZIP) [file pone.0299776.s004.zip › 55079943_20210602R/55079943_20210602R_zone.png]

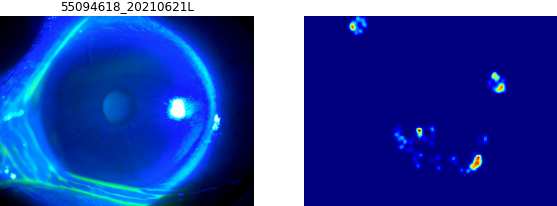

Supplement: S3 Dataset — (ZIP) [file pone.0299776.s004.zip › 55094618_20210621L/55094618_20210621L_densitymap.png]

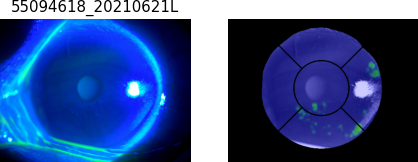

Supplement: S3 Dataset — (ZIP) [file pone.0299776.s004.zip › 55094618_20210621L/55094618_20210621L_whole.png]

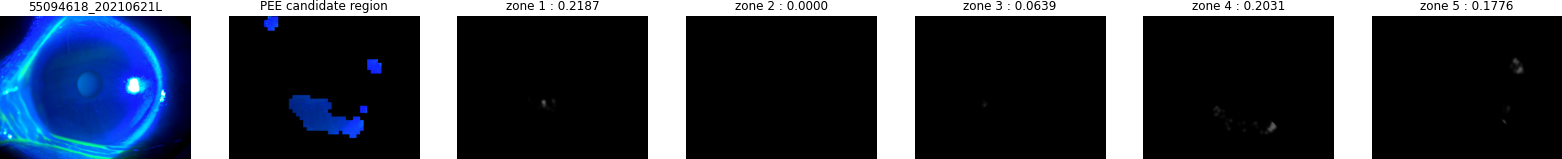

Supplement: S3 Dataset — (ZIP) [file pone.0299776.s004.zip › 55094618_20210621L/55094618_20210621L_zone.png]

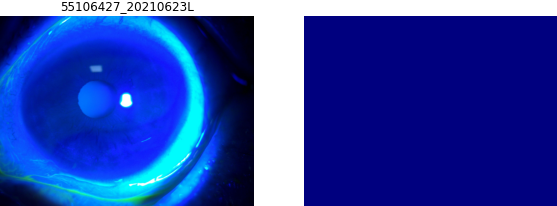

Supplement: S3 Dataset — (ZIP) [file pone.0299776.s004.zip › 55106427_20210623L/55106427_20210623L_densitymap.png]

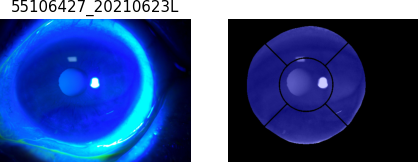

Supplement: S3 Dataset — (ZIP) [file pone.0299776.s004.zip › 55106427_20210623L/55106427_20210623L_whole.png]

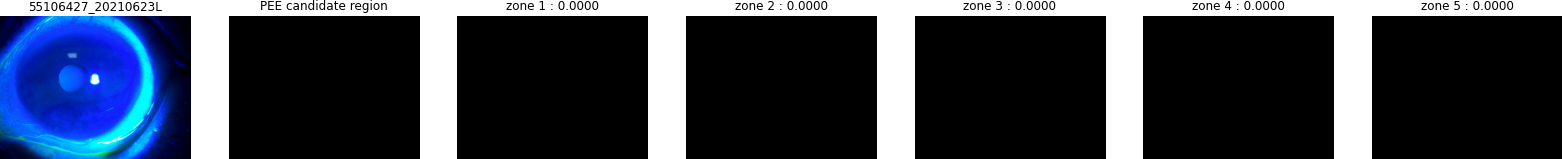

Supplement: S3 Dataset — (ZIP) [file pone.0299776.s004.zip › 55106427_20210623L/55106427_20210623L_zone.png]

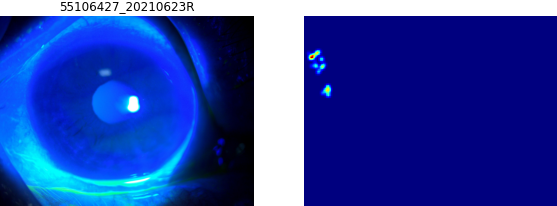

Supplement: S3 Dataset — (ZIP) [file pone.0299776.s004.zip › 55106427_20210623R/55106427_20210623R_densitymap.png]

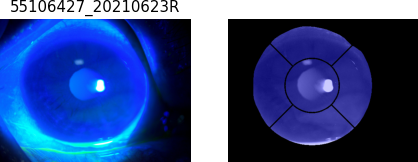

Supplement: S3 Dataset — (ZIP) [file pone.0299776.s004.zip › 55106427_20210623R/55106427_20210623R_whole.png]

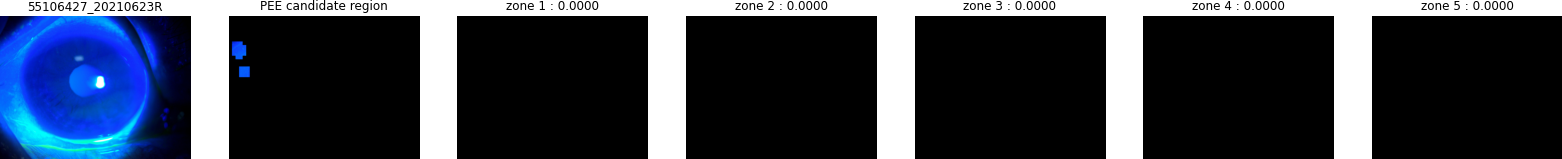

Supplement: S3 Dataset — (ZIP) [file pone.0299776.s004.zip › 55106427_20210623R/55106427_20210623R_zone.png]

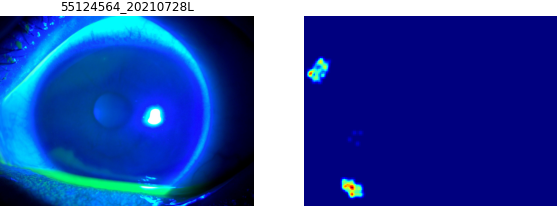

Supplement: S3 Dataset — (ZIP) [file pone.0299776.s004.zip › 55124564_20210728L/55124564_20210728L_densitymap.png]

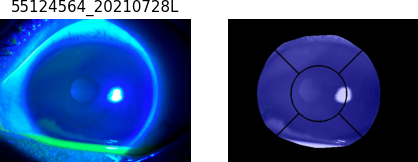

Supplement: S3 Dataset — (ZIP) [file pone.0299776.s004.zip › 55124564_20210728L/55124564_20210728L_whole.png]

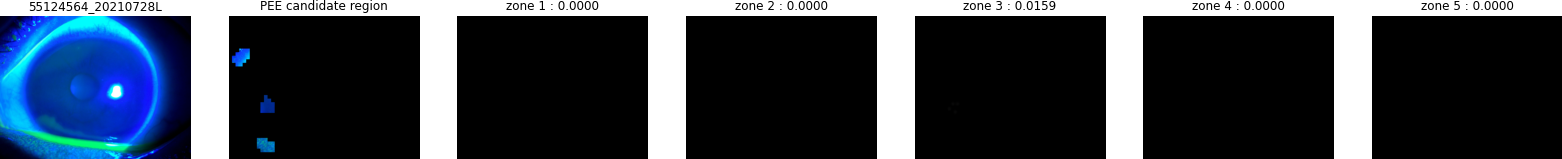

Supplement: S3 Dataset — (ZIP) [file pone.0299776.s004.zip › 55124564_20210728L/55124564_20210728L_zone.png]

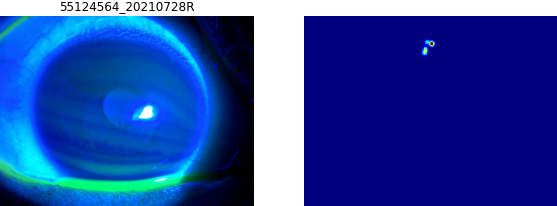

Supplement: S3 Dataset — (ZIP) [file pone.0299776.s004.zip › 55124564_20210728R/55124564_20210728R_densitymap.png]

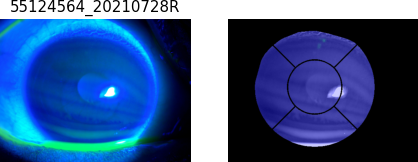

Supplement: S3 Dataset — (ZIP) [file pone.0299776.s004.zip › 55124564_20210728R/55124564_20210728R_whole.png]

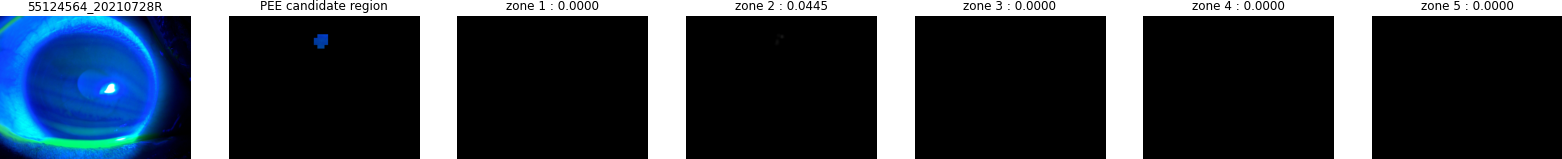

Supplement: S3 Dataset — (ZIP) [file pone.0299776.s004.zip › 55124564_20210728R/55124564_20210728R_zone.png]

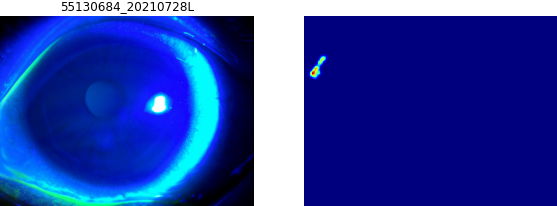

Supplement: S3 Dataset — (ZIP) [file pone.0299776.s004.zip › 55130684_20210728L/55130684_20210728L_densitymap.png]

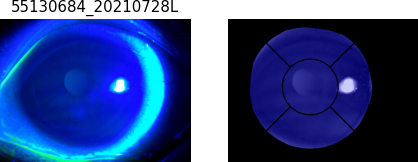

Supplement: S3 Dataset — (ZIP) [file pone.0299776.s004.zip › 55130684_20210728L/55130684_20210728L_whole.png]

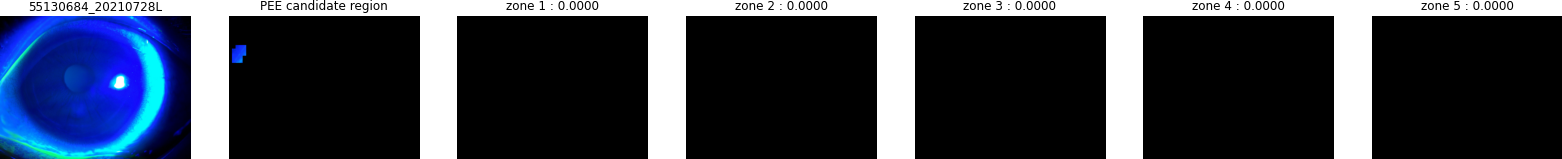

Supplement: S3 Dataset — (ZIP) [file pone.0299776.s004.zip › 55130684_20210728L/55130684_20210728L_zone.png]

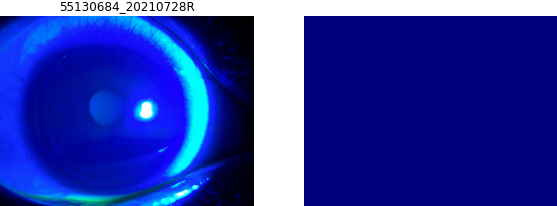

Supplement: S3 Dataset — (ZIP) [file pone.0299776.s004.zip › 55130684_20210728R/55130684_20210728R_densitymap.png]

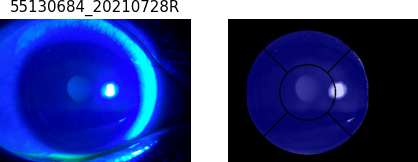

Supplement: S3 Dataset — (ZIP) [file pone.0299776.s004.zip › 55130684_20210728R/55130684_20210728R_whole.png]

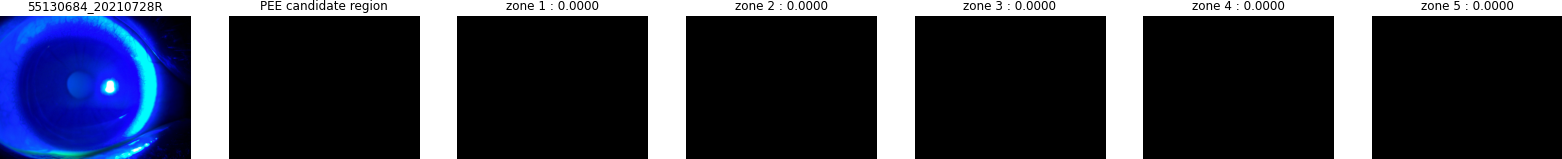

Supplement: S3 Dataset — (ZIP) [file pone.0299776.s004.zip › 55130684_20210728R/55130684_20210728R_zone.png]

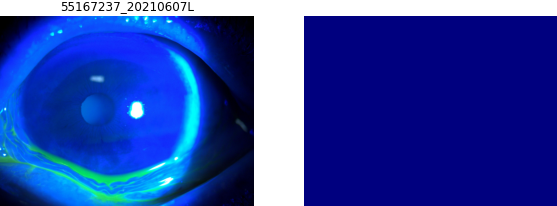

Supplement: S3 Dataset — (ZIP) [file pone.0299776.s004.zip › 55167237_20210607L/55167237_20210607L_densitymap.png]

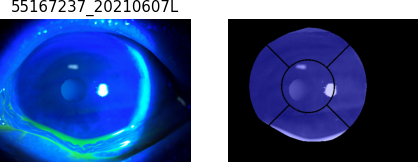

Supplement: S3 Dataset — (ZIP) [file pone.0299776.s004.zip › 55167237_20210607L/55167237_20210607L_whole.png]

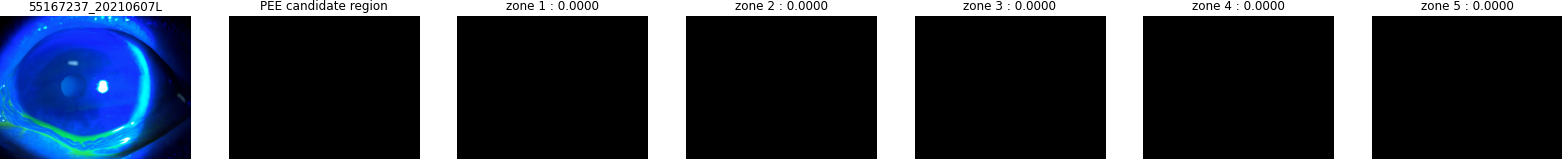

Supplement: S3 Dataset — (ZIP) [file pone.0299776.s004.zip › 55167237_20210607L/55167237_20210607L_zone.png]

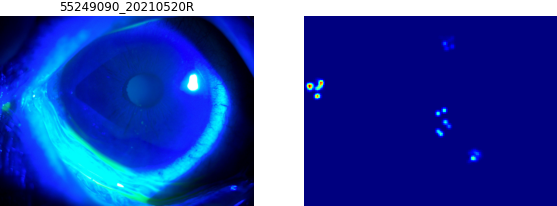

Supplement: S3 Dataset — (ZIP) [file pone.0299776.s004.zip › 55249090_20210520R/55249090_20210520R_densitymap.png]

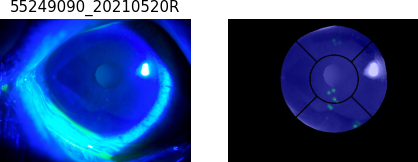

Supplement: S3 Dataset — (ZIP) [file pone.0299776.s004.zip › 55249090_20210520R/55249090_20210520R_whole.png]

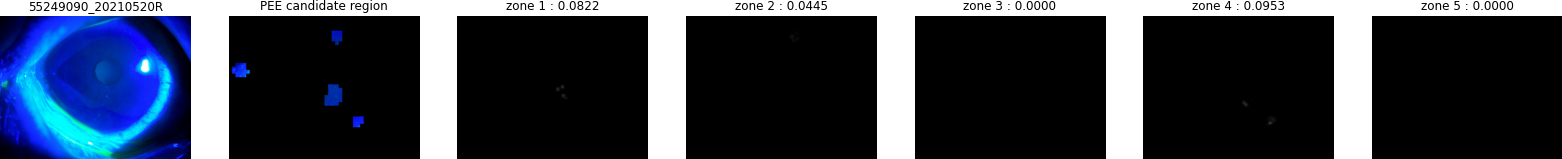

Supplement: S3 Dataset — (ZIP) [file pone.0299776.s004.zip › 55249090_20210520R/55249090_20210520R_zone.png]

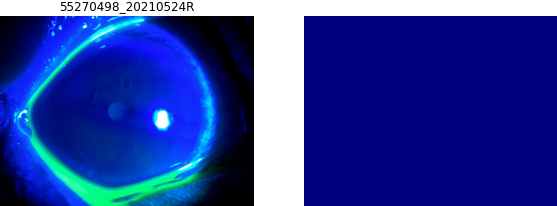

Supplement: S3 Dataset — (ZIP) [file pone.0299776.s004.zip › 55270498_20210524R/55270498_20210524R_densitymap.png]

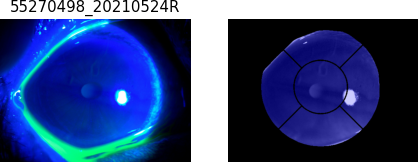

Supplement: S3 Dataset — (ZIP) [file pone.0299776.s004.zip › 55270498_20210524R/55270498_20210524R_whole.png]

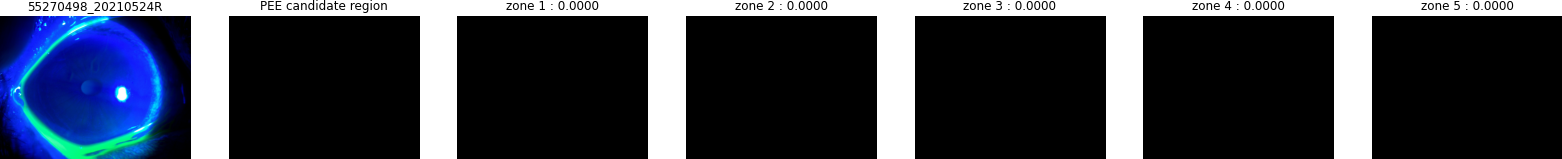

Supplement: S3 Dataset — (ZIP) [file pone.0299776.s004.zip › 55270498_20210524R/55270498_20210524R_zone.png]

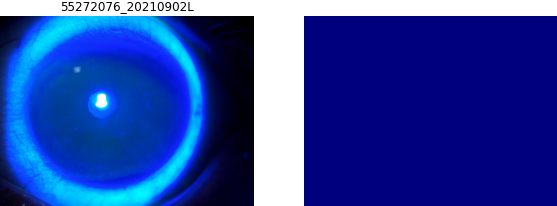

Supplement: S3 Dataset — (ZIP) [file pone.0299776.s004.zip › 55272076_20210902L/55272076_20210902L_densitymap.png]

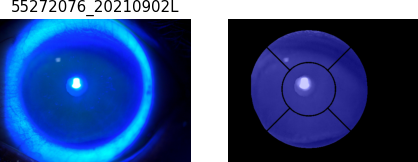

Supplement: S3 Dataset — (ZIP) [file pone.0299776.s004.zip › 55272076_20210902L/55272076_20210902L_whole.png]

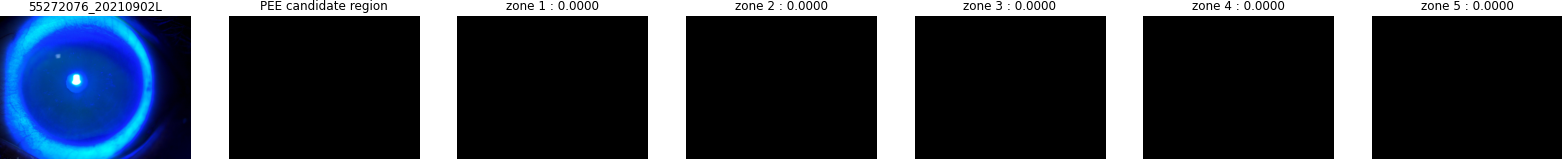

Supplement: S3 Dataset — (ZIP) [file pone.0299776.s004.zip › 55272076_20210902L/55272076_20210902L_zone.png]

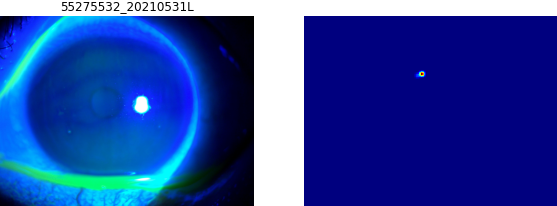

Supplement: S3 Dataset — (ZIP) [file pone.0299776.s004.zip › 55275532_20210531L/55275532_20210531L_densitymap.png]

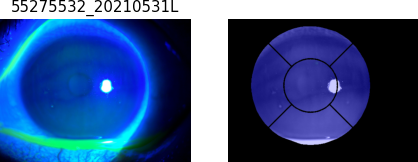

Supplement: S3 Dataset — (ZIP) [file pone.0299776.s004.zip › 55275532_20210531L/55275532_20210531L_whole.png]

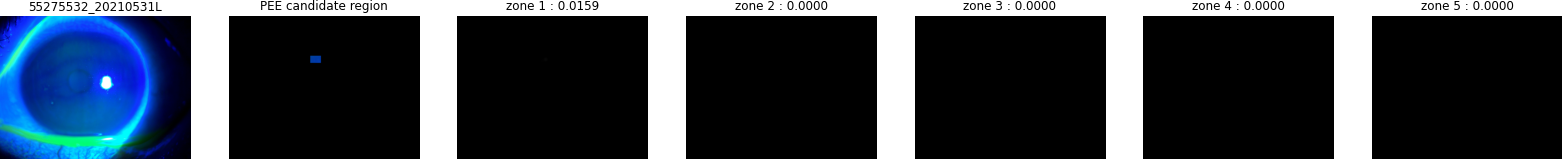

Supplement: S3 Dataset — (ZIP) [file pone.0299776.s004.zip › 55275532_20210531L/55275532_20210531L_zone.png]

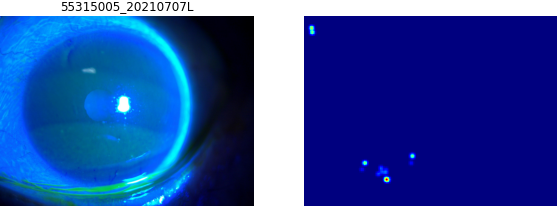

Supplement: S3 Dataset — (ZIP) [file pone.0299776.s004.zip › 55315005_20210707L/55315005_20210707L_densitymap.png]

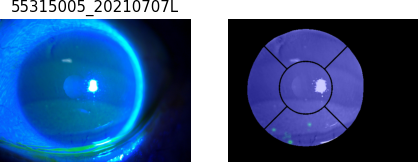

Supplement: S3 Dataset — (ZIP) [file pone.0299776.s004.zip › 55315005_20210707L/55315005_20210707L_whole.png]

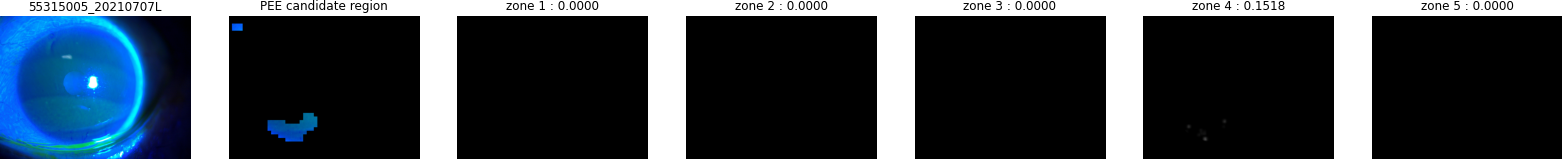

Supplement: S3 Dataset — (ZIP) [file pone.0299776.s004.zip › 55315005_20210707L/55315005_20210707L_zone.png]

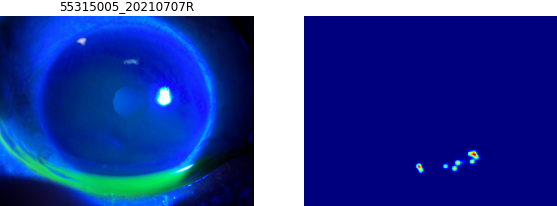

Supplement: S3 Dataset — (ZIP) [file pone.0299776.s004.zip › 55315005_20210707R/55315005_20210707R_densitymap.png]

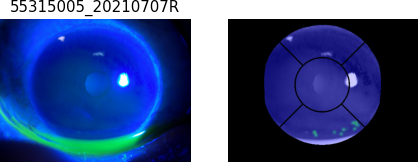

Supplement: S3 Dataset — (ZIP) [file pone.0299776.s004.zip › 55315005_20210707R/55315005_20210707R_whole.png]

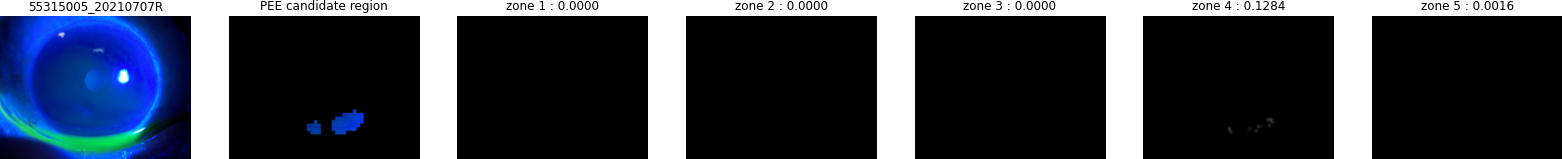

Supplement: S3 Dataset — (ZIP) [file pone.0299776.s004.zip › 55315005_20210707R/55315005_20210707R_zone.png]

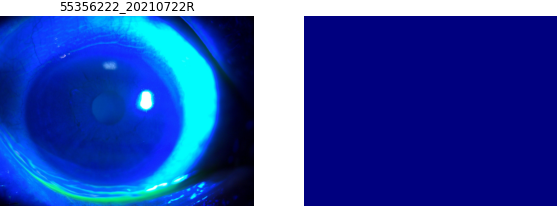

Supplement: S3 Dataset — (ZIP) [file pone.0299776.s004.zip › 55356222_20210722R/55356222_20210722R_densitymap.png]

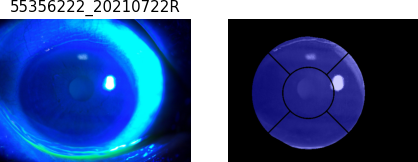

Supplement: S3 Dataset — (ZIP) [file pone.0299776.s004.zip › 55356222_20210722R/55356222_20210722R_whole.png]
