# Supplementary figures and images for: Deep learning-based fully automated grading system for dry eye disease severity (part 5 of 6)
Source: PLoS One. 2024 Mar 14;19(3):e0299776. doi: 10.1371/journal.pone.0299776 (PMC10939279; doi:10.1371/journal.pone.0299776)

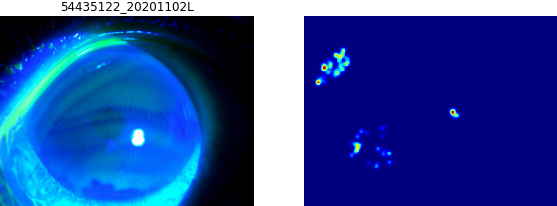

Supplement: S3 Dataset — (ZIP) [file pone.0299776.s004.zip › 54435122_20201102L/54435122_20201102L_densitymap.png]

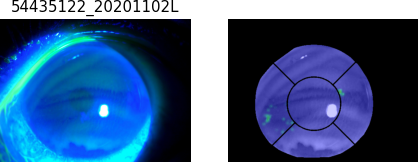

Supplement: S3 Dataset — (ZIP) [file pone.0299776.s004.zip › 54435122_20201102L/54435122_20201102L_whole.png]

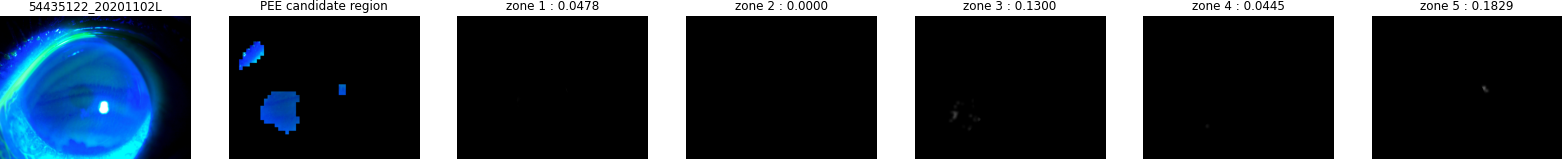

Supplement: S3 Dataset — (ZIP) [file pone.0299776.s004.zip › 54435122_20201102L/54435122_20201102L_zone.png]

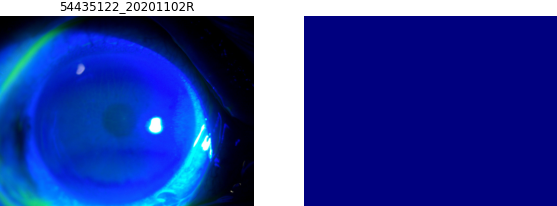

Supplement: S3 Dataset — (ZIP) [file pone.0299776.s004.zip › 54435122_20201102R/54435122_20201102R_densitymap.png]

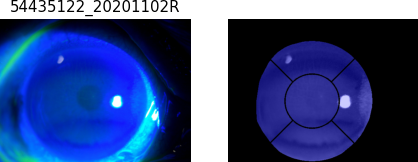

Supplement: S3 Dataset — (ZIP) [file pone.0299776.s004.zip › 54435122_20201102R/54435122_20201102R_whole.png]

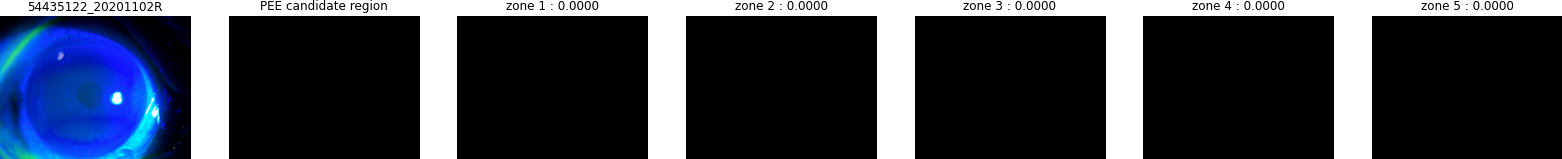

Supplement: S3 Dataset — (ZIP) [file pone.0299776.s004.zip › 54435122_20201102R/54435122_20201102R_zone.png]

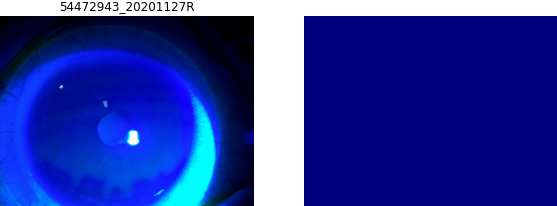

Supplement: S3 Dataset — (ZIP) [file pone.0299776.s004.zip › 54472943_20201127R/54472943_20201127R_densitymap.png]

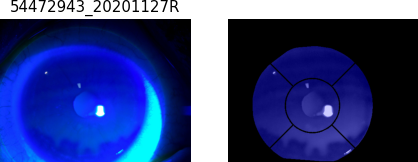

Supplement: S3 Dataset — (ZIP) [file pone.0299776.s004.zip › 54472943_20201127R/54472943_20201127R_whole.png]

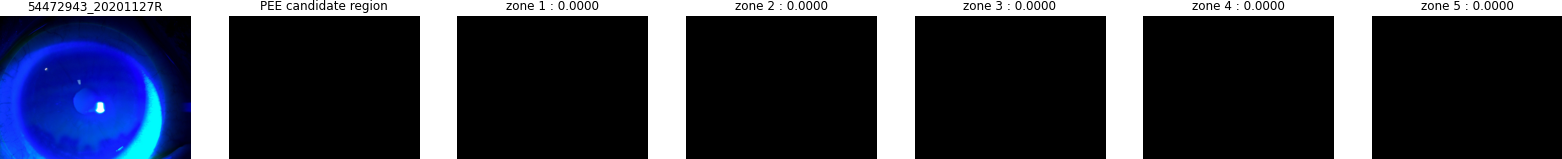

Supplement: S3 Dataset — (ZIP) [file pone.0299776.s004.zip › 54472943_20201127R/54472943_20201127R_zone.png]

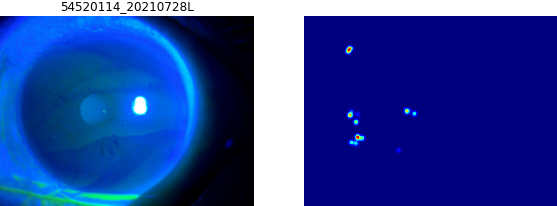

Supplement: S3 Dataset — (ZIP) [file pone.0299776.s004.zip › 54520114_20210728L/54520114_20210728L_densitymap.png]

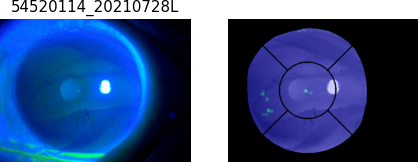

Supplement: S3 Dataset — (ZIP) [file pone.0299776.s004.zip › 54520114_20210728L/54520114_20210728L_whole.png]

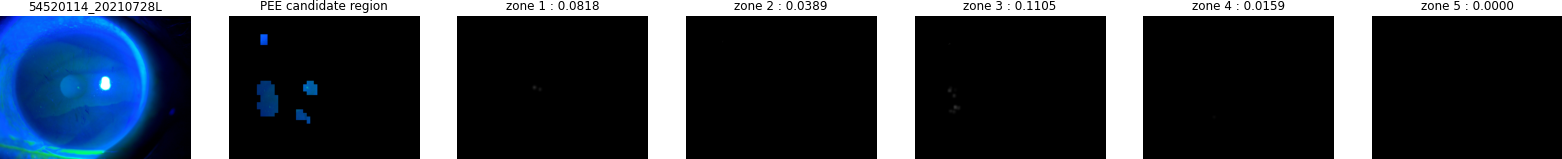

Supplement: S3 Dataset — (ZIP) [file pone.0299776.s004.zip › 54520114_20210728L/54520114_20210728L_zone.png]

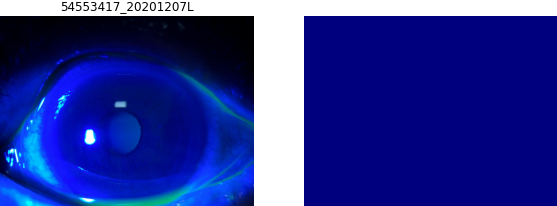

Supplement: S3 Dataset — (ZIP) [file pone.0299776.s004.zip › 54553417_20201207L/54553417_20201207L_densitymap.png]

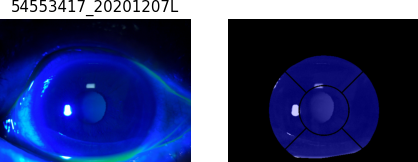

Supplement: S3 Dataset — (ZIP) [file pone.0299776.s004.zip › 54553417_20201207L/54553417_20201207L_whole.png]

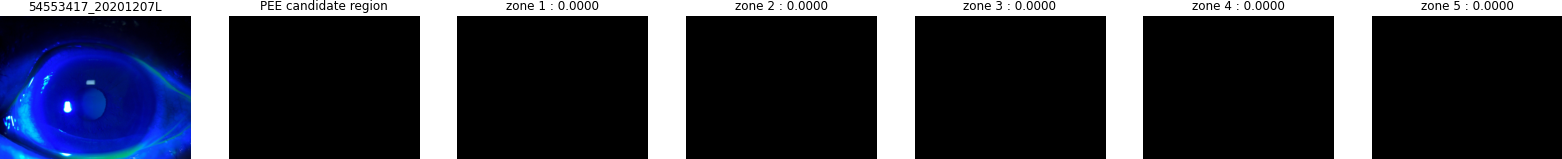

Supplement: S3 Dataset — (ZIP) [file pone.0299776.s004.zip › 54553417_20201207L/54553417_20201207L_zone.png]

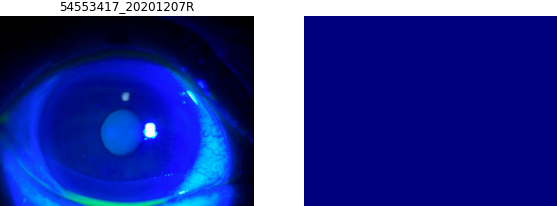

Supplement: S3 Dataset — (ZIP) [file pone.0299776.s004.zip › 54553417_20201207R/54553417_20201207R_densitymap.png]

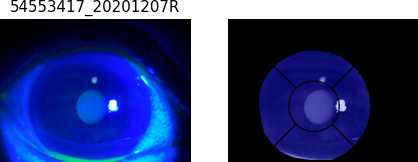

Supplement: S3 Dataset — (ZIP) [file pone.0299776.s004.zip › 54553417_20201207R/54553417_20201207R_whole.png]

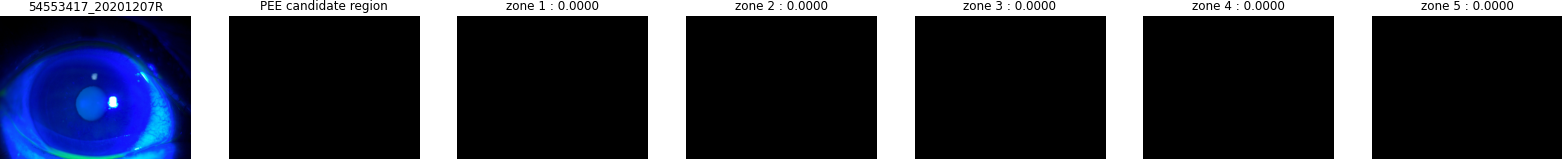

Supplement: S3 Dataset — (ZIP) [file pone.0299776.s004.zip › 54553417_20201207R/54553417_20201207R_zone.png]

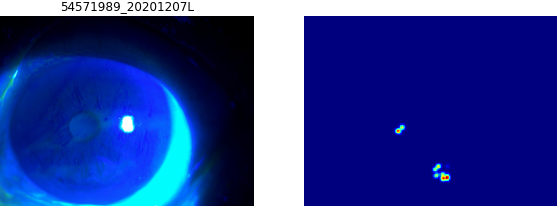

Supplement: S3 Dataset — (ZIP) [file pone.0299776.s004.zip › 54571989_20201207L/54571989_20201207L_densitymap.png]

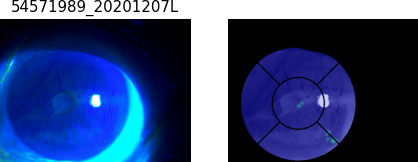

Supplement: S3 Dataset — (ZIP) [file pone.0299776.s004.zip › 54571989_20201207L/54571989_20201207L_whole.png]

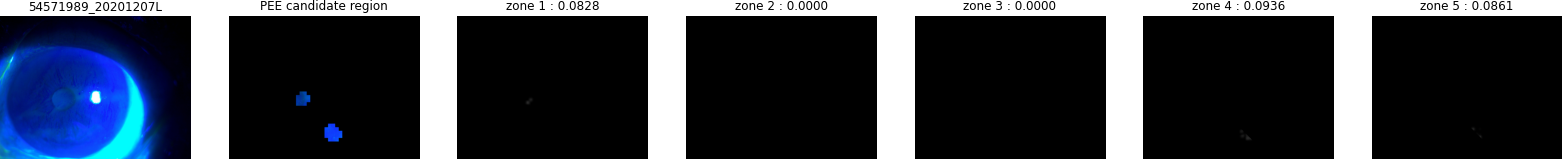

Supplement: S3 Dataset — (ZIP) [file pone.0299776.s004.zip › 54571989_20201207L/54571989_20201207L_zone.png]

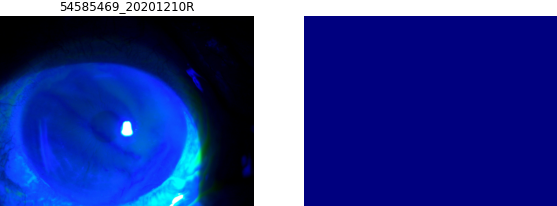

Supplement: S3 Dataset — (ZIP) [file pone.0299776.s004.zip › 54585469_20201210R/54585469_20201210R_densitymap.png]

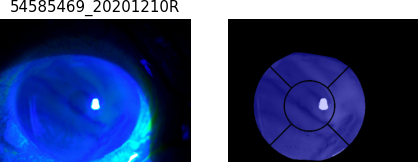

Supplement: S3 Dataset — (ZIP) [file pone.0299776.s004.zip › 54585469_20201210R/54585469_20201210R_whole.png]

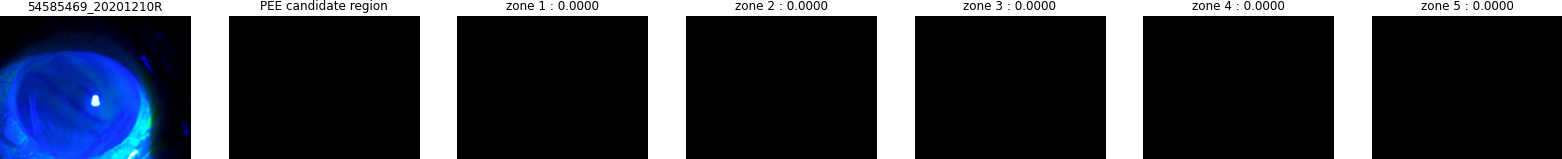

Supplement: S3 Dataset — (ZIP) [file pone.0299776.s004.zip › 54585469_20201210R/54585469_20201210R_zone.png]

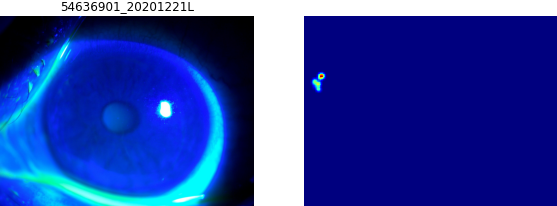

Supplement: S3 Dataset — (ZIP) [file pone.0299776.s004.zip › 54636901_20201221L/54636901_20201221L_densitymap.png]

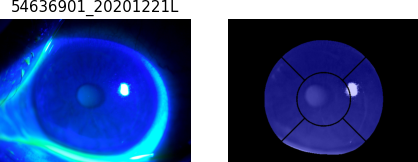

Supplement: S3 Dataset — (ZIP) [file pone.0299776.s004.zip › 54636901_20201221L/54636901_20201221L_whole.png]

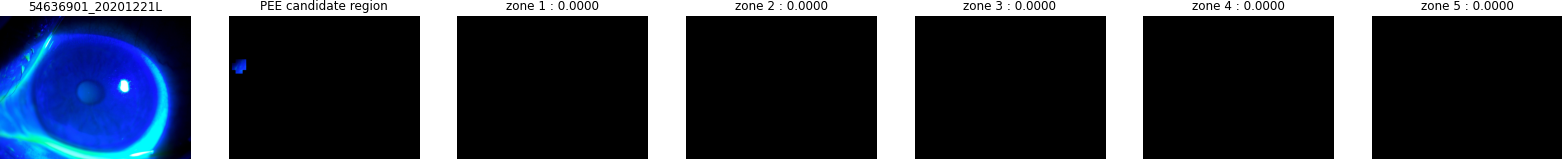

Supplement: S3 Dataset — (ZIP) [file pone.0299776.s004.zip › 54636901_20201221L/54636901_20201221L_zone.png]

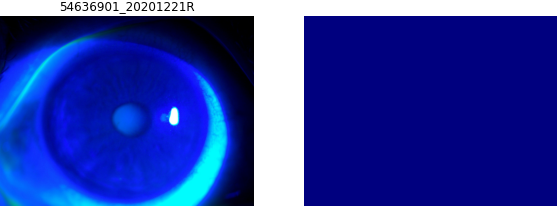

Supplement: S3 Dataset — (ZIP) [file pone.0299776.s004.zip › 54636901_20201221R/54636901_20201221R_densitymap.png]

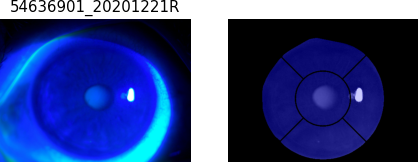

Supplement: S3 Dataset — (ZIP) [file pone.0299776.s004.zip › 54636901_20201221R/54636901_20201221R_whole.png]

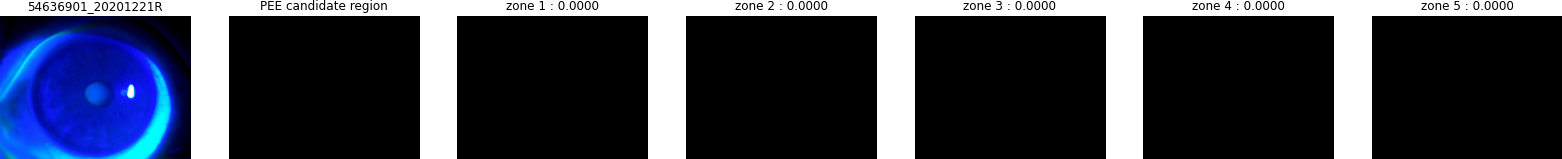

Supplement: S3 Dataset — (ZIP) [file pone.0299776.s004.zip › 54636901_20201221R/54636901_20201221R_zone.png]

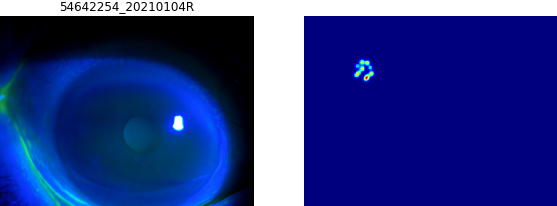

Supplement: S3 Dataset — (ZIP) [file pone.0299776.s004.zip › 54642254_20210104R/54642254_20210104R_densitymap.png]

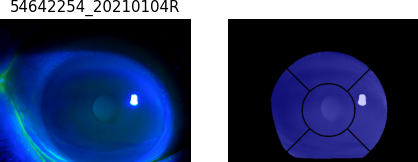

Supplement: S3 Dataset — (ZIP) [file pone.0299776.s004.zip › 54642254_20210104R/54642254_20210104R_whole.png]

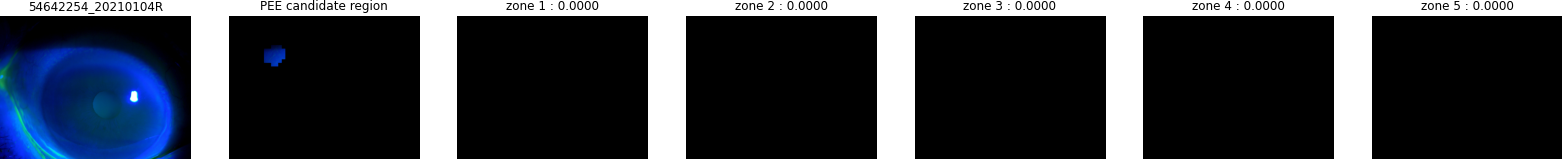

Supplement: S3 Dataset — (ZIP) [file pone.0299776.s004.zip › 54642254_20210104R/54642254_20210104R_zone.png]

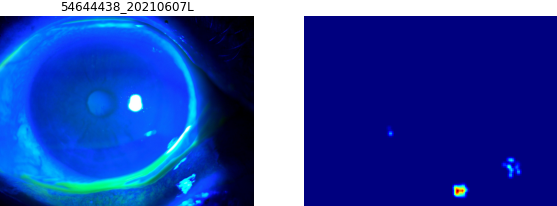

Supplement: S3 Dataset — (ZIP) [file pone.0299776.s004.zip › 54644438_20210607L/54644438_20210607L_densitymap.png]

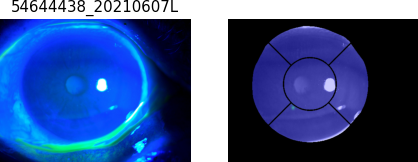

Supplement: S3 Dataset — (ZIP) [file pone.0299776.s004.zip › 54644438_20210607L/54644438_20210607L_whole.png]

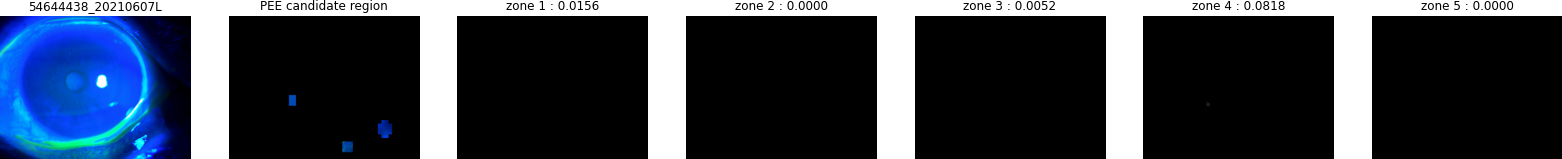

Supplement: S3 Dataset — (ZIP) [file pone.0299776.s004.zip › 54644438_20210607L/54644438_20210607L_zone.png]

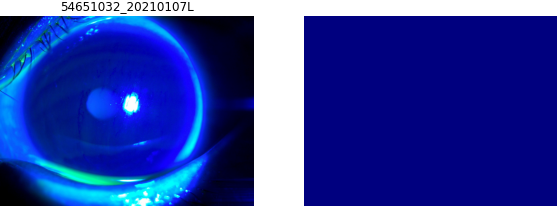

Supplement: S3 Dataset — (ZIP) [file pone.0299776.s004.zip › 54651032_20210107L/54651032_20210107L_densitymap.png]

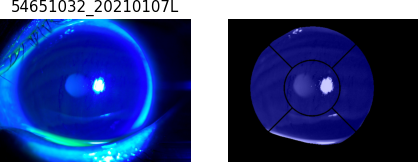

Supplement: S3 Dataset — (ZIP) [file pone.0299776.s004.zip › 54651032_20210107L/54651032_20210107L_whole.png]

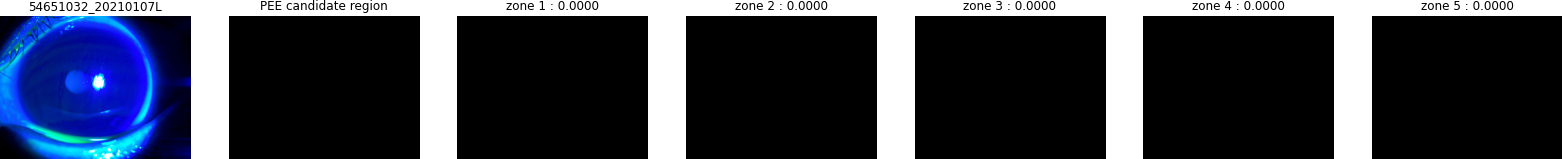

Supplement: S3 Dataset — (ZIP) [file pone.0299776.s004.zip › 54651032_20210107L/54651032_20210107L_zone.png]

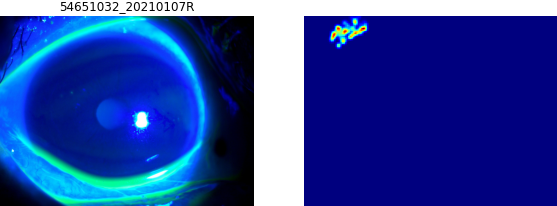

Supplement: S3 Dataset — (ZIP) [file pone.0299776.s004.zip › 54651032_20210107R/54651032_20210107R_densitymap.png]

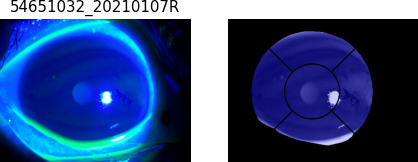

Supplement: S3 Dataset — (ZIP) [file pone.0299776.s004.zip › 54651032_20210107R/54651032_20210107R_whole.png]

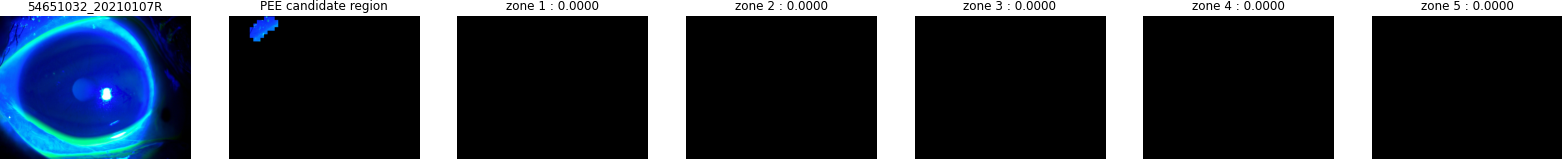

Supplement: S3 Dataset — (ZIP) [file pone.0299776.s004.zip › 54651032_20210107R/54651032_20210107R_zone.png]

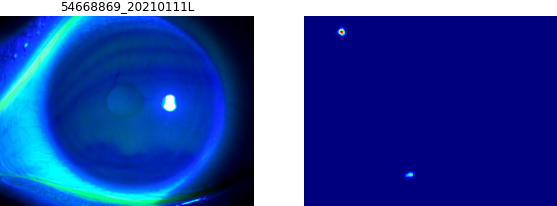

Supplement: S3 Dataset — (ZIP) [file pone.0299776.s004.zip › 54668869_20210111L/54668869_20210111L_densitymap.png]

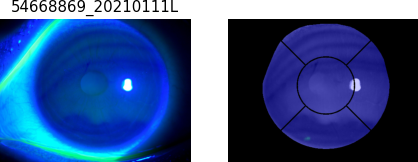

Supplement: S3 Dataset — (ZIP) [file pone.0299776.s004.zip › 54668869_20210111L/54668869_20210111L_whole.png]

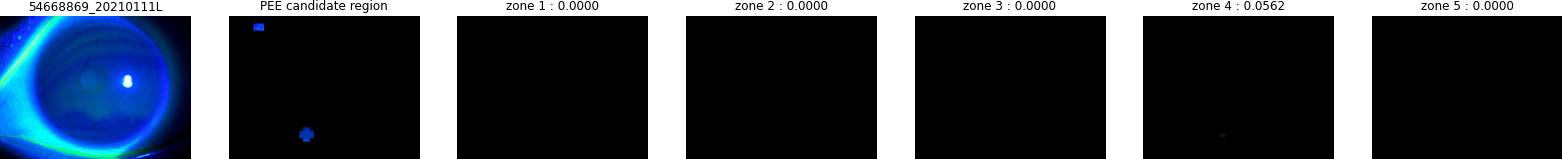

Supplement: S3 Dataset — (ZIP) [file pone.0299776.s004.zip › 54668869_20210111L/54668869_20210111L_zone.png]

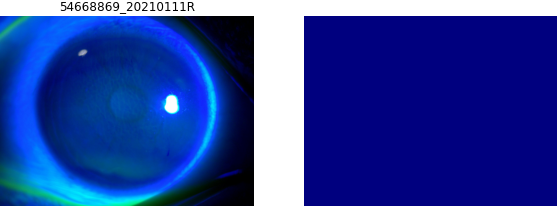

Supplement: S3 Dataset — (ZIP) [file pone.0299776.s004.zip › 54668869_20210111R/54668869_20210111R_densitymap.png]

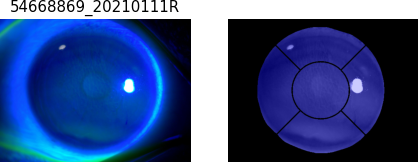

Supplement: S3 Dataset — (ZIP) [file pone.0299776.s004.zip › 54668869_20210111R/54668869_20210111R_whole.png]

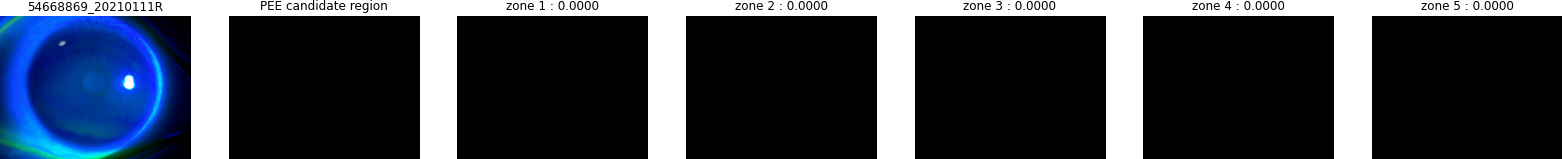

Supplement: S3 Dataset — (ZIP) [file pone.0299776.s004.zip › 54668869_20210111R/54668869_20210111R_zone.png]

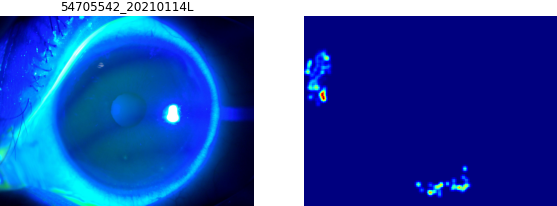

Supplement: S3 Dataset — (ZIP) [file pone.0299776.s004.zip › 54705542_20210114L/54705542_20210114L_densitymap.png]

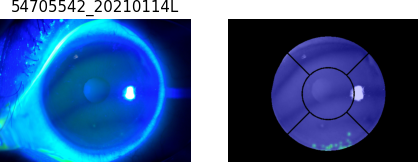

Supplement: S3 Dataset — (ZIP) [file pone.0299776.s004.zip › 54705542_20210114L/54705542_20210114L_whole.png]

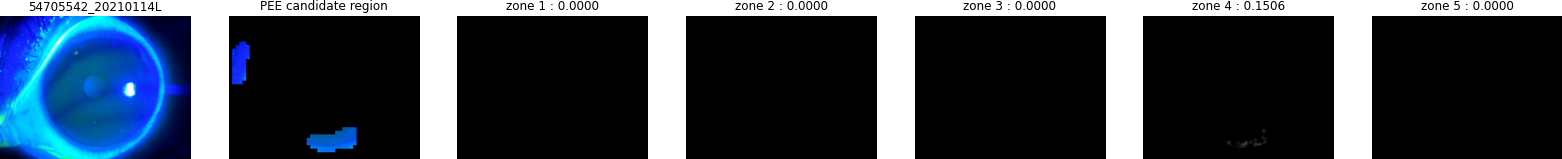

Supplement: S3 Dataset — (ZIP) [file pone.0299776.s004.zip › 54705542_20210114L/54705542_20210114L_zone.png]

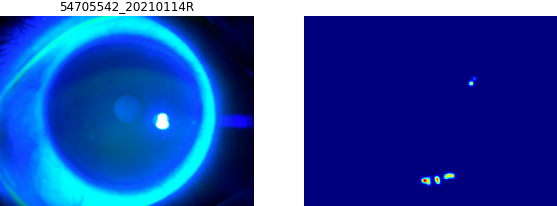

Supplement: S3 Dataset — (ZIP) [file pone.0299776.s004.zip › 54705542_20210114R/54705542_20210114R_densitymap.png]

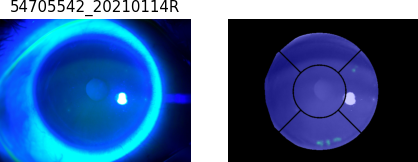

Supplement: S3 Dataset — (ZIP) [file pone.0299776.s004.zip › 54705542_20210114R/54705542_20210114R_whole.png]

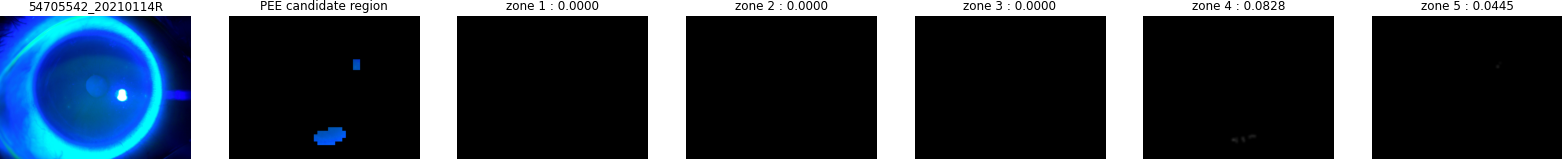

Supplement: S3 Dataset — (ZIP) [file pone.0299776.s004.zip › 54705542_20210114R/54705542_20210114R_zone.png]

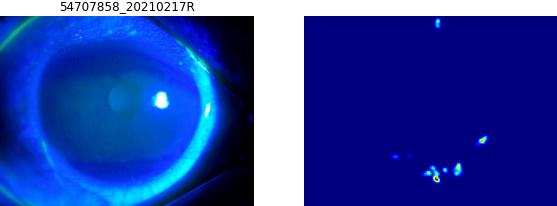

Supplement: S3 Dataset — (ZIP) [file pone.0299776.s004.zip › 54707858_20210217R/54707858_20210217R_densitymap.png]

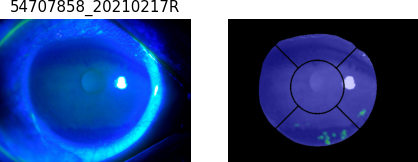

Supplement: S3 Dataset — (ZIP) [file pone.0299776.s004.zip › 54707858_20210217R/54707858_20210217R_whole.png]

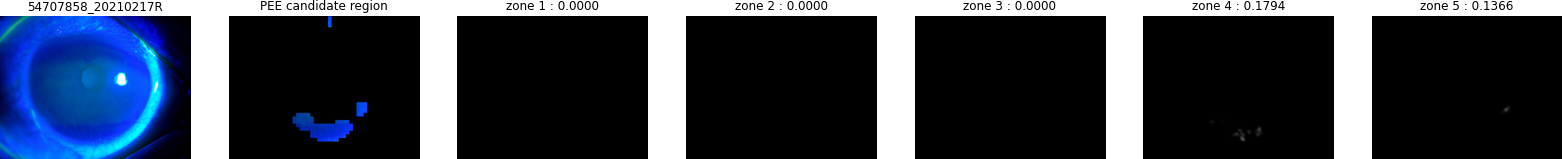

Supplement: S3 Dataset — (ZIP) [file pone.0299776.s004.zip › 54707858_20210217R/54707858_20210217R_zone.png]

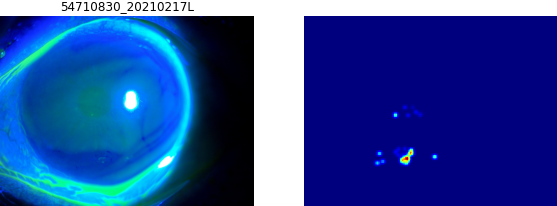

Supplement: S3 Dataset — (ZIP) [file pone.0299776.s004.zip › 54710830_20210217L/54710830_20210217L_densitymap.png]

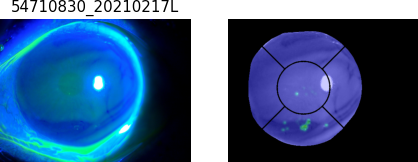

Supplement: S3 Dataset — (ZIP) [file pone.0299776.s004.zip › 54710830_20210217L/54710830_20210217L_whole.png]

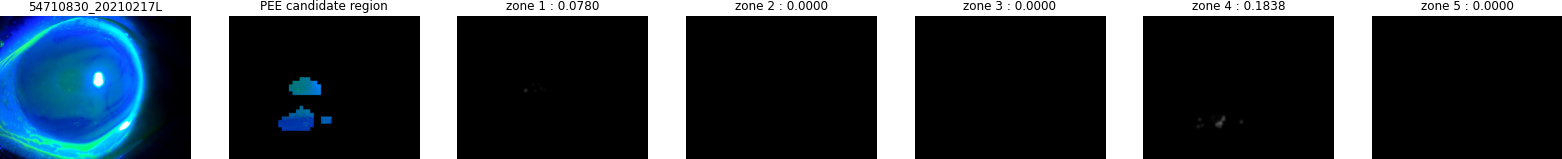

Supplement: S3 Dataset — (ZIP) [file pone.0299776.s004.zip › 54710830_20210217L/54710830_20210217L_zone.png]

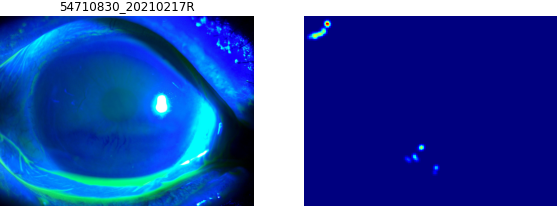

Supplement: S3 Dataset — (ZIP) [file pone.0299776.s004.zip › 54710830_20210217R/54710830_20210217R_densitymap.png]

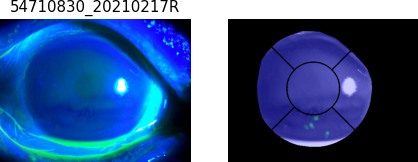

Supplement: S3 Dataset — (ZIP) [file pone.0299776.s004.zip › 54710830_20210217R/54710830_20210217R_whole.png]

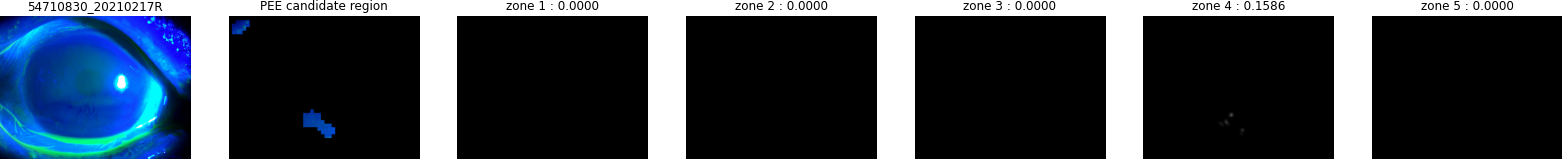

Supplement: S3 Dataset — (ZIP) [file pone.0299776.s004.zip › 54710830_20210217R/54710830_20210217R_zone.png]

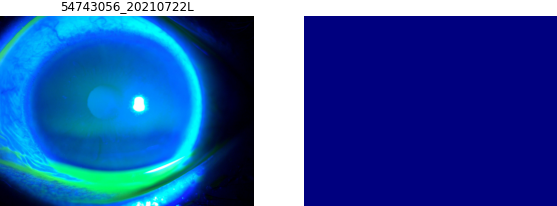

Supplement: S3 Dataset — (ZIP) [file pone.0299776.s004.zip › 54743056_20210722L/54743056_20210722L_densitymap.png]

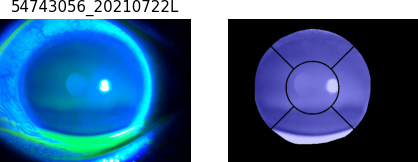

Supplement: S3 Dataset — (ZIP) [file pone.0299776.s004.zip › 54743056_20210722L/54743056_20210722L_whole.png]

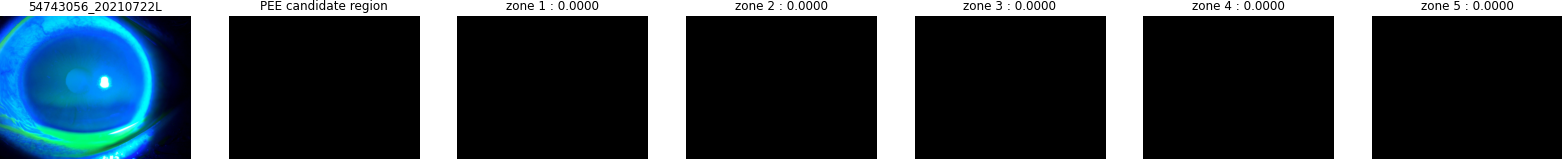

Supplement: S3 Dataset — (ZIP) [file pone.0299776.s004.zip › 54743056_20210722L/54743056_20210722L_zone.png]

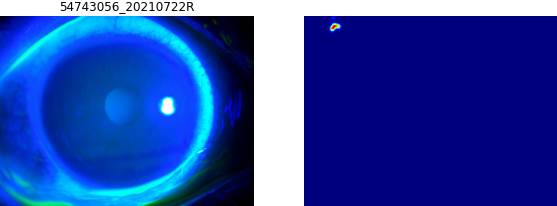

Supplement: S3 Dataset — (ZIP) [file pone.0299776.s004.zip › 54743056_20210722R/54743056_20210722R_densitymap.png]

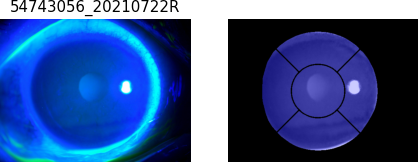

Supplement: S3 Dataset — (ZIP) [file pone.0299776.s004.zip › 54743056_20210722R/54743056_20210722R_whole.png]

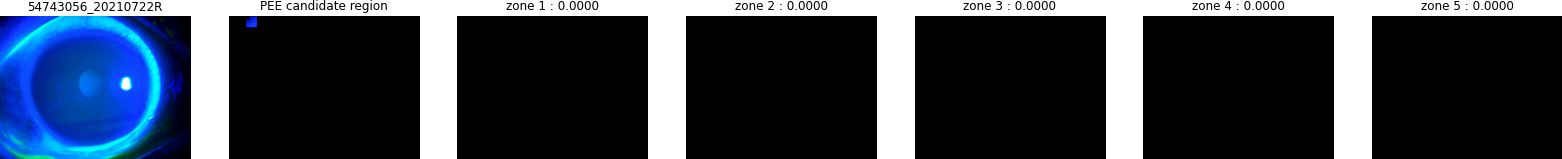

Supplement: S3 Dataset — (ZIP) [file pone.0299776.s004.zip › 54743056_20210722R/54743056_20210722R_zone.png]

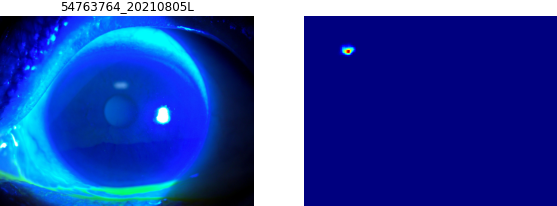

Supplement: S3 Dataset — (ZIP) [file pone.0299776.s004.zip › 54763764_20210805L/54763764_20210805L_densitymap.png]

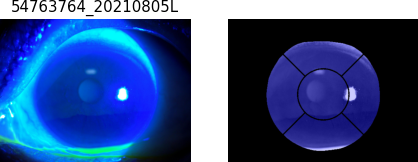

Supplement: S3 Dataset — (ZIP) [file pone.0299776.s004.zip › 54763764_20210805L/54763764_20210805L_whole.png]

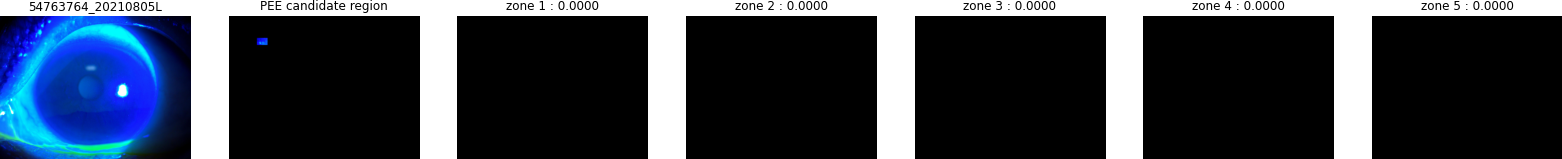

Supplement: S3 Dataset — (ZIP) [file pone.0299776.s004.zip › 54763764_20210805L/54763764_20210805L_zone.png]

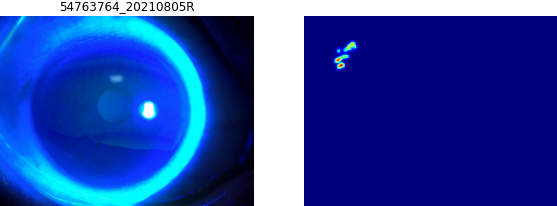

Supplement: S3 Dataset — (ZIP) [file pone.0299776.s004.zip › 54763764_20210805R/54763764_20210805R_densitymap.png]

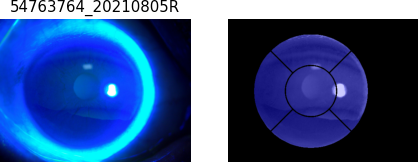

Supplement: S3 Dataset — (ZIP) [file pone.0299776.s004.zip › 54763764_20210805R/54763764_20210805R_whole.png]

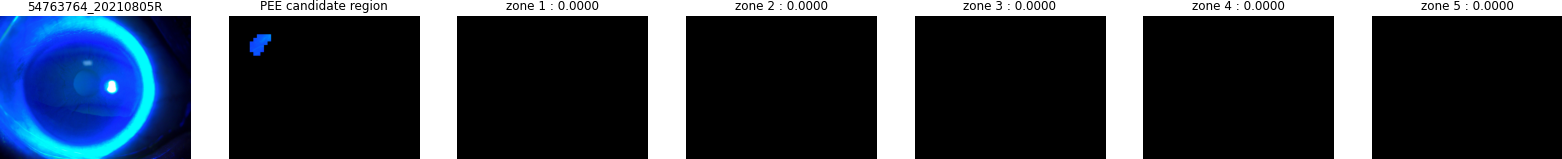

Supplement: S3 Dataset — (ZIP) [file pone.0299776.s004.zip › 54763764_20210805R/54763764_20210805R_zone.png]

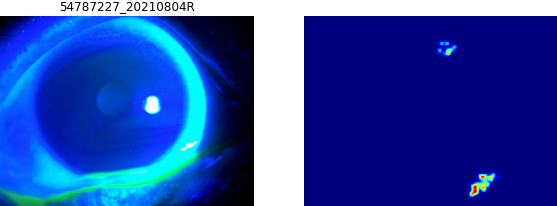

Supplement: S3 Dataset — (ZIP) [file pone.0299776.s004.zip › 54787227_20210804R/54787227_20210804R_densitymap.png]

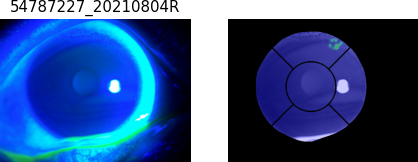

Supplement: S3 Dataset — (ZIP) [file pone.0299776.s004.zip › 54787227_20210804R/54787227_20210804R_whole.png]

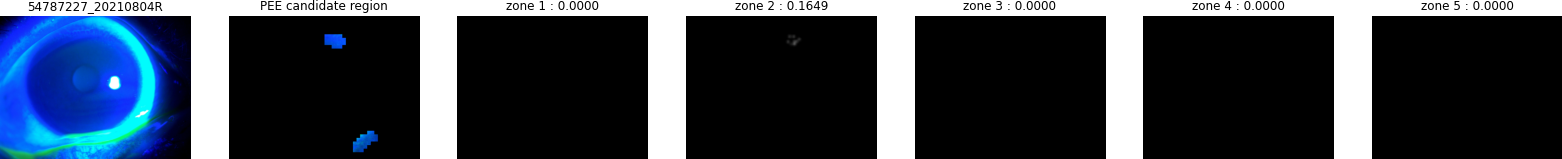

Supplement: S3 Dataset — (ZIP) [file pone.0299776.s004.zip › 54787227_20210804R/54787227_20210804R_zone.png]

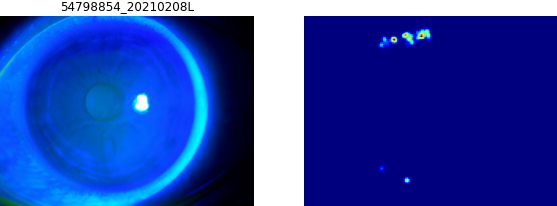

Supplement: S3 Dataset — (ZIP) [file pone.0299776.s004.zip › 54798854_20210208L/54798854_20210208L_densitymap.png]

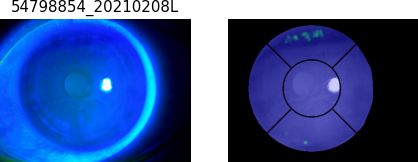

Supplement: S3 Dataset — (ZIP) [file pone.0299776.s004.zip › 54798854_20210208L/54798854_20210208L_whole.png]

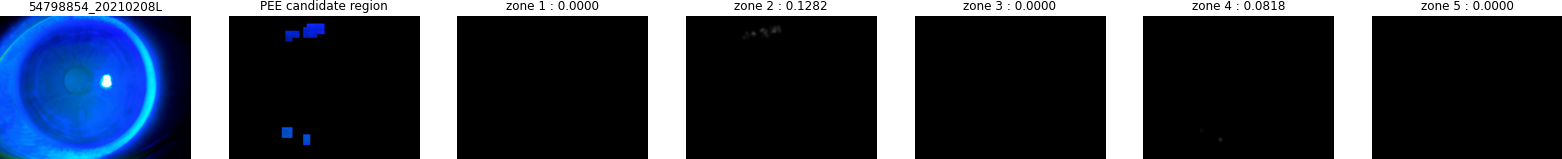

Supplement: S3 Dataset — (ZIP) [file pone.0299776.s004.zip › 54798854_20210208L/54798854_20210208L_zone.png]

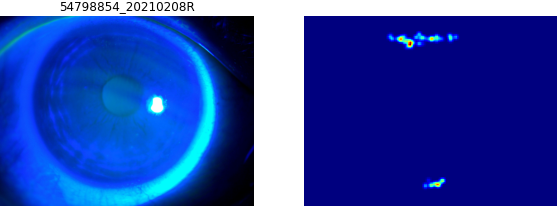

Supplement: S3 Dataset — (ZIP) [file pone.0299776.s004.zip › 54798854_20210208R/54798854_20210208R_densitymap.png]

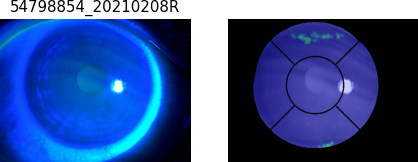

Supplement: S3 Dataset — (ZIP) [file pone.0299776.s004.zip › 54798854_20210208R/54798854_20210208R_whole.png]

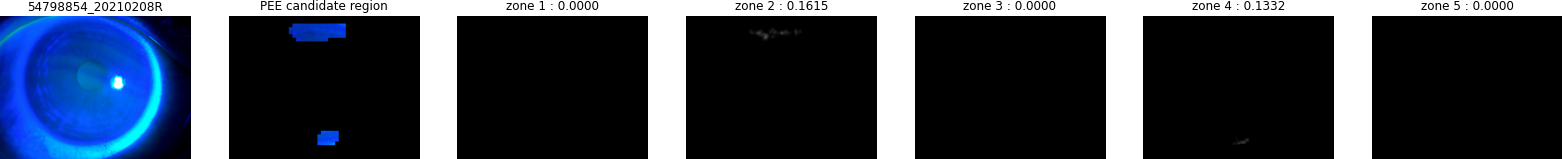

Supplement: S3 Dataset — (ZIP) [file pone.0299776.s004.zip › 54798854_20210208R/54798854_20210208R_zone.png]

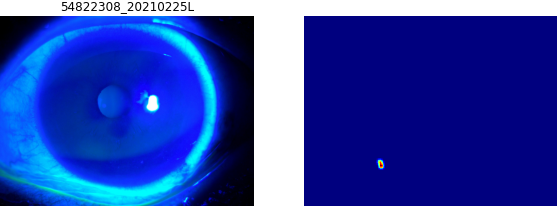

Supplement: S3 Dataset — (ZIP) [file pone.0299776.s004.zip › 54822308_20210225L/54822308_20210225L_densitymap.png]

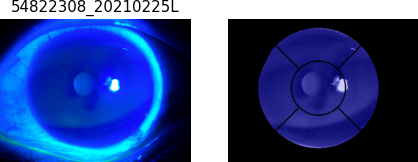

Supplement: S3 Dataset — (ZIP) [file pone.0299776.s004.zip › 54822308_20210225L/54822308_20210225L_whole.png]

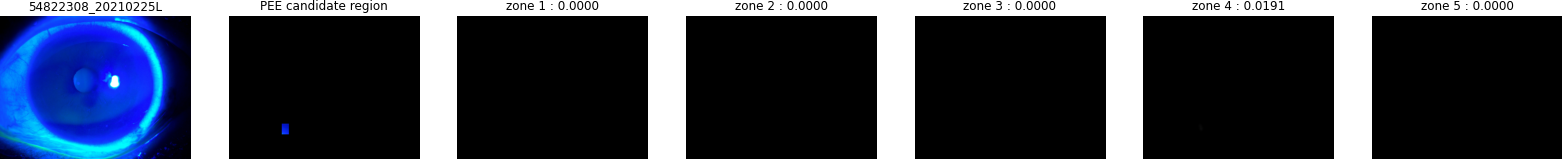

Supplement: S3 Dataset — (ZIP) [file pone.0299776.s004.zip › 54822308_20210225L/54822308_20210225L_zone.png]

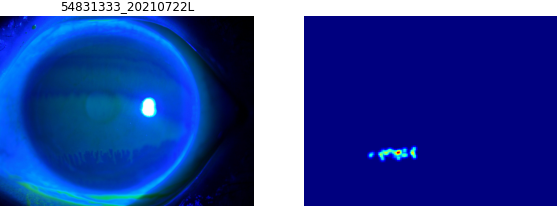

Supplement: S3 Dataset — (ZIP) [file pone.0299776.s004.zip › 54831333_20210722L/54831333_20210722L_densitymap.png]

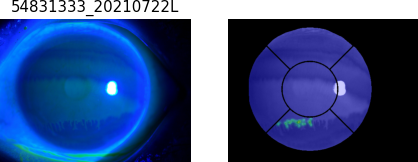

Supplement: S3 Dataset — (ZIP) [file pone.0299776.s004.zip › 54831333_20210722L/54831333_20210722L_whole.png]

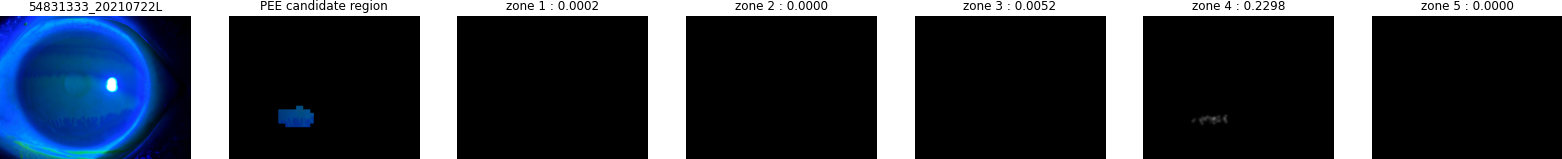

Supplement: S3 Dataset — (ZIP) [file pone.0299776.s004.zip › 54831333_20210722L/54831333_20210722L_zone.png]

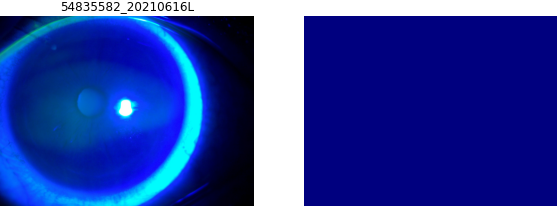

Supplement: S3 Dataset — (ZIP) [file pone.0299776.s004.zip › 54835582_20210616L/54835582_20210616L_densitymap.png]

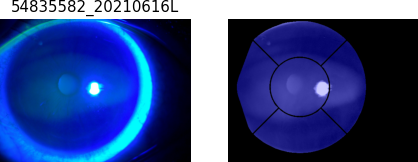

Supplement: S3 Dataset — (ZIP) [file pone.0299776.s004.zip › 54835582_20210616L/54835582_20210616L_whole.png]

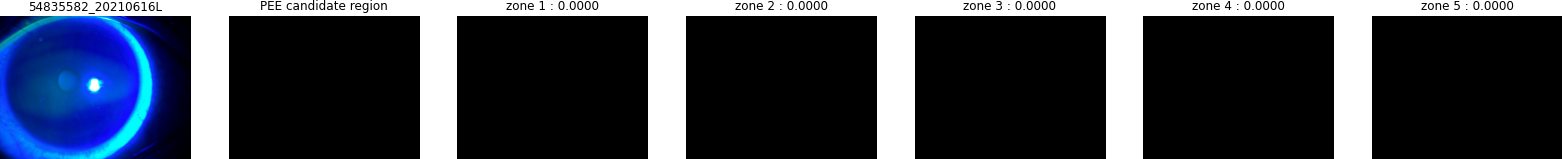

Supplement: S3 Dataset — (ZIP) [file pone.0299776.s004.zip › 54835582_20210616L/54835582_20210616L_zone.png]

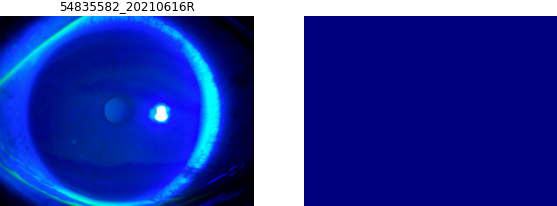

Supplement: S3 Dataset — (ZIP) [file pone.0299776.s004.zip › 54835582_20210616R/54835582_20210616R_densitymap.png]

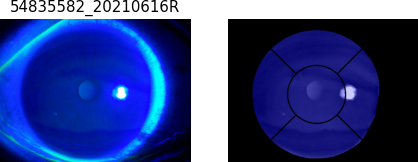

Supplement: S3 Dataset — (ZIP) [file pone.0299776.s004.zip › 54835582_20210616R/54835582_20210616R_whole.png]

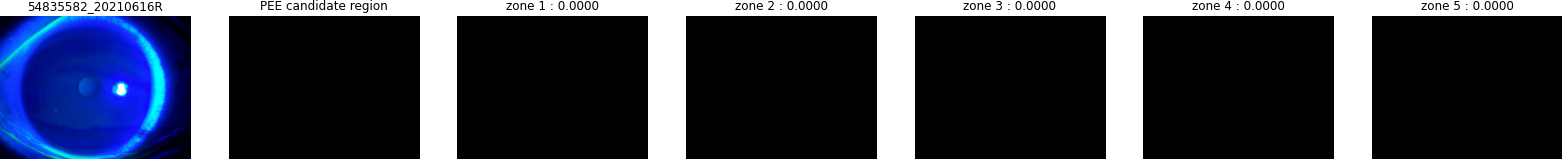

Supplement: S3 Dataset — (ZIP) [file pone.0299776.s004.zip › 54835582_20210616R/54835582_20210616R_zone.png]

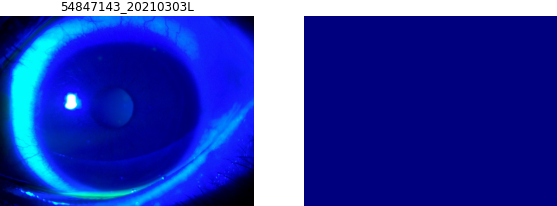

Supplement: S3 Dataset — (ZIP) [file pone.0299776.s004.zip › 54847143_20210303L/54847143_20210303L_densitymap.png]

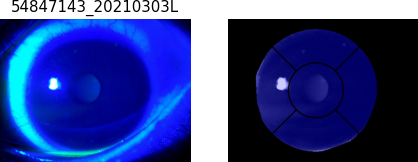

Supplement: S3 Dataset — (ZIP) [file pone.0299776.s004.zip › 54847143_20210303L/54847143_20210303L_whole.png]

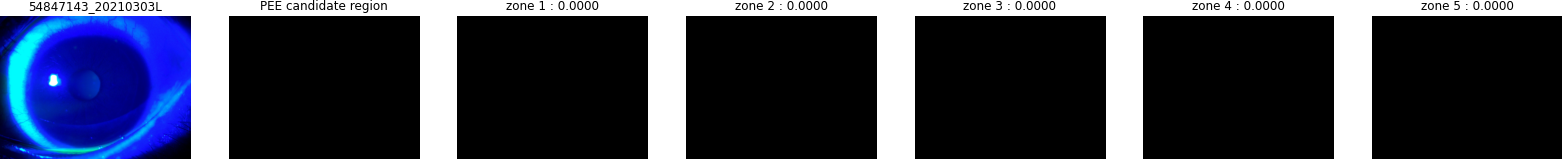

Supplement: S3 Dataset — (ZIP) [file pone.0299776.s004.zip › 54847143_20210303L/54847143_20210303L_zone.png]

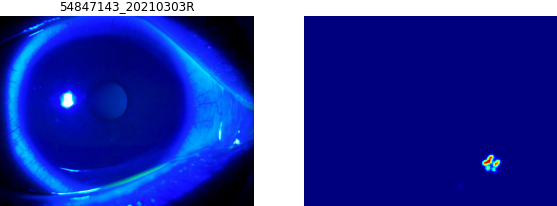

Supplement: S3 Dataset — (ZIP) [file pone.0299776.s004.zip › 54847143_20210303R/54847143_20210303R_densitymap.png]
